# Clarifying Values: An Updated and Expanded Systematic Review and Meta-Analysis

**DOI:** 10.1177/0272989X211037946

**Published:** 2021-09-25

**Authors:** Holly O. Witteman, Ruth Ndjaboue, Gratianne Vaisson, Selma Chipenda Dansokho, Bob Arnold, John F. P. Bridges, Sandrine Comeau, Angela Fagerlin, Teresa Gavaruzzi, Melina Marcoux, Arwen Pieterse, Michael Pignone, Thierry Provencher, Charles Racine, Dean Regier, Charlotte Rochefort-Brihay, Praveen Thokala, Marieke Weernink, Douglas B. White, Celia E. Wills, Jesse Jansen

**Affiliations:** Department of Family and Emergency Medicine, Faculty of Medicine, Laval University, Quebec City, Quebec, Canada; VITAM Research Centre, Quebec City, Quebec, Canada; CHU de Québec Research Centre, Quebec City, Quebec, Canada; Department of Family and Emergency Medicine, Faculty of Medicine, Laval University, Quebec City, Quebec, Canada; VITAM Research Centre, Quebec City, Quebec, Canada; Department of Family and Emergency Medicine, Faculty of Medicine, Laval University, Quebec City, Quebec, Canada; CHU de Québec Research Centre, Quebec City, Quebec, Canada; Department of Family and Emergency Medicine, Faculty of Medicine, Laval University, Quebec City, Quebec, Canada; UPMC Palliative and Supportive Institute, Division of General Internal Medicine, Section of Palliative Care and Medical Ethics, University of Pittsburgh, Pittsburgh, PA, USA; Department of Biomedical Informatics, The Ohio State University College of Medicine, Columbus, OH, USA; Department of Family and Emergency Medicine, Faculty of Medicine, Laval University, Quebec City, Quebec, Canada; Department of Population Health Sciences, University of Utah School of Medicine, Salt Lake City, UT, USA; Department of Developmental Psychology and Socialization, University of Padova, Padova, Italy; Department of Family and Emergency Medicine, Faculty of Medicine, Laval University, Quebec City, Quebec, Canada; Leiden University Medical Center, Leiden, The Netherlands; Departments of Internal Medicine and Population Health, Dell Medical School, University of Texas, Austin, TX, USA; Department of Family and Emergency Medicine, Faculty of Medicine, Laval University, Quebec City, Quebec, Canada; Department of Family and Emergency Medicine, Faculty of Medicine, Laval University, Quebec City, Quebec, Canada; School of Population and Public Health, University of British Columbia, Vancouver, British Columbia, Canada; Department of Family and Emergency Medicine, Faculty of Medicine, Laval University, Quebec City, Quebec, Canada; School of Health and Related Research, University of Sheffield, Sheffield, UK; Municipal Health Services (GGD), Enschede, The Netherlands; Program on Ethics and Decision Making in Critical Illness, Department of Critical Care Medicine, University of Pittsburgh School of Medicine, Pittsburgh, PA, USA; College of Nursing, Center on Healthy Aging, Self-Management and Complex Care, The Ohio State University, Columbus, OH, USA; Department of Family Medicine/CAPHRI, Maastricht University, Maastricht, The Netherlands

**Keywords:** decision making, values clarification, shared decision making, preference elicitation

## Abstract

**Background:**

Patient decision aids should help people make evidence-informed decisions aligned with their values. There is limited guidance about how to achieve such alignment.

**Purpose:**

To describe the range of values clarification methods available to patient decision aid developers, synthesize evidence regarding their relative merits, and foster collection of evidence by offering researchers a proposed set of outcomes to report when evaluating the effects of values clarification methods.

**Data Sources:**

MEDLINE, EMBASE, PubMed, Web of Science, the Cochrane Library, and CINAHL.

**Study Selection:**

We included articles that described randomized trials of 1 or more explicit values clarification methods. From 30,648 records screened, we identified 33 articles describing trials of 43 values clarification methods.

**Data Extraction:**

Two independent reviewers extracted details about each values clarification method and its evaluation.

**Data Synthesis:**

Compared to control conditions or to implicit values clarification methods, explicit values clarification methods decreased the frequency of values-incongruent choices (risk difference, –0.04; 95% confidence interval [CI], –0.06 to –0.02; *P* < 0.001) and decisional conflict (standardized mean difference, –0.20; 95% CI, –0.29 to –0.11; *P* < 0.001). Multicriteria decision analysis led to more values-congruent decisions than other values clarification methods (χ^2^ = 9.25, *P* = 0.01). There were no differences between different values clarification methods regarding decisional conflict (χ^2^ = 6.08, *P* = 0.05).

**Limitations:**

Some meta-analyses had high heterogeneity. We grouped values clarification methods into broad categories.

**Conclusions:**

Current evidence suggests patient decision aids should include an explicit values clarification method. Developers may wish to specifically consider multicriteria decision analysis. Future evaluations of values clarification methods should report their effects on decisional conflict, decisions made, values congruence, and decisional regret.

**Highlights**

- Current evidence suggests patient decision aids should include an explicit values clarification method.
- To support health decisions that align with values, patient decision aid developers may wish to specifically consider multicriteria decision analysis.

## Introduction

Shared decision making is appropriate in many situations and is particularly indicated in clinical situations where the “best” option may differ between people, depending on what matters to them.^1–3^ What is important to one person might be different from what is important to others, and determining what is important can be difficult even with the appropriate information and evidence at hand. The process of shared decision making therefore aims to help people make health-related decisions that are informed by high-quality, well-presented, and well-understood evidence,^4–8^ aligned with what matters to the person or people affected by the decision and acted upon.^9–12^ It follows that the process of clarifying and expressing values is an important aspect of shared decision making and thus of patient decision aids. Within patient decision aids, this process is supported by explicit values clarification methods.

Explicit values clarification methods require users to interact with something such as a worksheet or an interactive website to clarify what matters to them relevant to a health decision. Such methods have been shown to encourage desirable outcomes such as better alignment with patients’ values^13,14^ and reduced decisional regret, the latter particularly among people with lower health literacy.^15^ However, explicit values clarification methods are extremely diverse,^16^ and there has been little guidance regarding their comparative effects on users’ decision making processes or outcomes,^17^ making it difficult for patient decision aid developers to know which explicit method to use. Patient decision aid developers might look toward the preference elicitation literature for guidance, but the guidance available^18^ is often tailored toward aggregate-level decision making, such as regulatory decisions^19^ or health technology assessment,^20^ not for supporting individual-level decision making.

This updated review sought to build upon previous versions of the International Patient Decision Aids Standards’ chapter on values clarification^21,22^ as well as previous evidence syntheses that have established the advantages of explicit values clarification methods over implicit methods or no values clarification.^13,14^ We sought to advance the science and practice of values clarification methods in 3 ways. First, we aimed to offer clear definitions and an annotated summary of existing approaches that have been or could be used as values clarification methods. Second, we aimed to synthesize evidence of different techniques’ effects on health decision outcomes. Third, we aimed to foster future evidence by offering researchers a proposed set of outcomes to consider when evaluating the effects of values clarification methods.

### Definitions

Part of the challenge in studying or using values clarification methods is that definitions vary and terms like *values* are used imprecisely in the patient decision support literature.^23,24^ Another challenge is that there is substantial overlap between values clarification methods used in patient decision support and preference elicitation methods used in health economics. To bring clarity to this imprecision and overlap, we adopt working definitions in Table 1 for use in this article.

**Table 1:** Definitions of Terms

Term	Definition Adopted in This Article
Values	An umbrella term referring to what matters to an individual relevant to a health decision. Values may be directly relevant to decisions (e.g., “beliefs, feelings, or perceptions regarding attributes of a treatment option”) or indirectly relevant (e.g., goals; worldviews; family, religious, or cultural values).^25^ Values may be represented qualitatively or, in some cases, quantitatively. This definition is deliberately broad.
Values clarification	“The process of sorting out what matters to an individual relevant to a given health decision.”^16^ This definition emphasizes that what matters to an individual may be broader than attribute-specific values. What matters may also include preferences, concerns (e.g., concerns about changes in health status), and issues to do with the context of a person’s life within which they would need to implement a decision (e.g., fitting a treatment plan into one’s work schedule).^16^
Values clarification methods	“Strategies that are intended to help patients evaluate the desirability of options or attributes of options within a specific decision context, in order to identify which option [they] prefer.”^22^
Implicit values clarification methods	Strategies for facilitating values clarification that do not require people to interact with anything or anyone—for example, describing “options in enough detail that clients can imagine what it is like to experience the physical, emotional, and social effects,”^14^ or simply encouraging people to think about what matters to them.
Explicit values clarification methods	Strategies for facilitating values clarification that require people to interact with something or someone (e.g., filling out a worksheet, using an interactive website, having a semistructured conversation with another person with the explicit purpose of clarifying values, or engaging in another structured exercise).
Preferences	The extent to which a decision option or health state is desirable or acceptable, either in the abstract or in comparison to other options or health states. Preferences may be represented qualitatively or, more commonly, quantitatively.^26^
Preference elicitation methods	Processes by which preferences are drawn out.^16^ Preference elicitation methods may vary according to the theory informing them. They are highly related to values clarification methods. Although older terms *revealed* and *stated* preference elicitation methods are no longer recommended, readers who encounter these terms in previous preference elicitation literature should note that these may overlap with implicit and explicit values clarification methods, respectively.
Tradeoffs	When multiple desirable outcomes cannot all be achieved, one must forgo (or trade off) some potential benefits or options to avail oneself of others. When users are explicitly required to engage with tradeoffs, this means they must consider and indicate what they are willing to give up to get something else or, in other words, which potential harms are acceptable in exchange for their associated potential benefits.^16^ For example, a user might indicate in a ranking exercise that they would prioritize greater comfort at the end of life over additional months of life.

As noted above, we continue to use the term *values clarification* even though this is sometimes misinterpreted as implying a narrow definition of values. Changing terms makes it difficult for people who are new to a field to connect the dots across decades of previous research. It is clear that previous research in values clarification addressed issues that were broader than valuation of treatment-specific attributes.^21^ In this update, we therefore move forward with the older terms, now with more clarity about what they mean in our presentation of the evidence.

### Theoretical Rationale

Our interdisciplinary team determined that the theoretical rationale for values clarification required only a small edit, shown in square brackets, to reflect the focus on explicit methods. Like Fagerlin et al.,^22^ we assert the theoretical rationale for explicit values clarification methods as being that they “should aim to [explicitly] facilitate at least one or more of the following six decision making processes: 1) Identifying options, which can include either the narrowing down of options, or the generation of options that were not offered at the outset, 2) Identifying attributes of the situation and/or the options which ultimately affect the patient’s preference in a specific decision context, 3) Reasoning about options or attributes of options, 4) Integrating attributes of options using either compensatory or both compensatory and noncompensatory decision rules, 5) Making holistic comparisons, and 6) Helping decision makers retrieve relevant values from long-term memory.” Pieterse et al.^27^ provided theory-based recommendations on processes that values clarification methods could aim to facilitate.

Although reasoning is one of the potential processes supported by values clarification, neither the definition nor the theoretical rationale of values clarification methods requires that people who are being supported in making a personal health decision must rationally deliberate about each option, or that the goal must always be a fully rational choice. In some decision making situations, rational deliberation and rational choice may be desired, while in others, they may not.^28,29^

### Explicit Values Clarification Methods

Table 2 organizes strategies that can be used as explicit values clarification methods in patient decision aids, building upon previously developed lists of types of values clarification methods^3,16^ and reviews of preference elicitation methods.^30,31^ Methods range from highly structured strategies that can also be used for preference elicitation in the context of health policy decision making to substantially less structured strategies. While not every use of a given method will be exactly the same, we deemed them functionally similar in terms of how they might be used and what the user experience might be in a patient decision aid. Patient decision aids may use multiple strategies. For example, a user may be asked to use a rating scale or visual analog scale whose values are then used in a decision-analytic model.

**Table 2:** Explicit Values Clarification Methods

Method	Description
Adaptive conjoint analysis (example^32^)	The user rates a series of sets of attributes and their levels, where choices presented are tailored to earlier answers.
Allocation of points (example^33^)	The user has a “budget” to “spend” on decision attributes, according to their importance.
Analytical hierarchy process (example^34^)	The user is asked to compare sets of options relative to predefined decision criteria.
Best–worst scaling (example^35^)	The user is asked to indicate the best and the worst object in repeated subsets of a finite number of objects (case 1, also known as object scaling or MaxDiff), the best and worst attributes within each of a number of profiles that systematically vary across a multiple attributes and levels (case 2), or the best and worst profiles from among 3 or more profiles (case 3).
Decision analysis or multicriteria decision analysis (umbrella term^a^) (resource^36,37^)	The user is asked to directly indicate the extent to which a decision attribute or outcome matters to them or how good or bad they deem it to be. These values are then used in a model that calculates alignment between what matters to the user and the available decision options.
Discrete-choice experiments (example^38^)	The user is asked to make a series of choices between 2 (or more) alternatives, where each alternative is characterized by attributes and their associated levels.
Open discussion (example^39^)	The user discusses what matters to them in an unstructured or semistructured discussion, possibly aided by a preset or user-created list of topics.
Pros and cons (resource^40^)	The user lists advantages (pros) and disadvantages (cons) of options and/or indicates the relevance (“this matters to me”) or importance (e.g., on a Likert scale) of each advantage or disadvantage.
Ranking (example^41^)	The user is asked to place attributes in order of importance, relative to each other.
Rating scales (example^42^)	The user indicates the importance of an attribute on a visual analog scale (e.g., paper-based visual analog scale, online slider) or Likert scale approximating a visual analog scale. If the rating is then used to calculate and show which option fits best, the method is classified as (multicriteria) decision analysis.
Social matching (example^43^)	The user “observes different characters’ decisions and/or decision-making processes and identifies 1 or more characters” with whom they identify.^16^
Standard gamble (example^44^)	The user indicates their choice between a) living the rest of their life in a particular health state (in the current context, a health state relevant to the health decision they are making) and b) taking a gamble between 2 possible outcomes: the probability *p* of living the remainder of their life in a state of optimal health and the probability 1 –*p* of immediate death.
Time tradeoff (example^44^)	The user indicates how many remaining lifetime years in full health they would be willing to give up (i.e., “trade off”) to avoid living for the rest of their life in the health state representing the decision making option of interest.

a *Multicriteria decision analysis* or *decision analysis* is an umbrella term. It encompasses some of the other, more specific categories (e.g., discrete-choice experiments, best–worst scaling.) When applicable, we use the more specific, narrower categories. Otherwise, we use the umbrella term *multicriteria decision analysis* or, for brevity in figures, *decision analysis*. In addition, although within multicriteria decision analysis, the user may be asked to rate attributes on rating scales, what distinguishes multicriteria decision analysis from other methods such as rating scales is that the model calculates how well or poorly the options align with what matters to a user.

## Methods

Our overall methods were guided by the Cochrane handbook. We report according to the Preferred Reporting Items for Systematic Reviews and Meta-Analyses (PRISMA)^45^ guidelines.

### Eligibility Criteria

We included published reports of comparative evaluations of explicit values clarification methods, whether they were called “values clarification methods” in the publications or not. This meant that we included trials of preference elicitation methods that had been trialed as values clarification methods (e.g., multicriteria decision analysis or discrete-choice experiments). We included evaluations using comparative methods (i.e., randomized controlled trials or randomized experiments of 1 or more values clarification methods). The comparisons could be 1 or more values clarification methods compared to a control method or compared to each other. Because we sought to understand the effects of values clarification methods, we excluded evaluations using descriptive study designs (e.g., acceptability and feasibility study, development study), observational study designs (e.g., reporting outcomes before and after use of a values clarification method), and reports of values clarification methods that did not evaluate the method independently of the patient decision aid in which it was used. Randomized experiments comparing 1 or more values clarification methods had to use distinctly different methods, meaning that more than the content or presentation of information in the values clarification method varied.

We did not apply language restrictions. We applied date restrictions to the portion of the review for which we had already conducted a systematic review (i.e., evaluations of values clarification methods that used the term *values clarification*).^17,22^ Specifically, for this subgroup, we added articles indexed or published starting in 2014 to the existing set of articles indexed or published prior to 2014 that we had already identified using the same search strategy. We applied no date restrictions to the new, expanded portion of the review (i.e., evaluations of values clarification methods that did not use the term *values clarification*).

### Information Sources

We performed a systematic literature search in MEDLINE, EMBASE, Web of Science, the Cochrane Library, and CINAHL.

### Search Strategy

We developed a draft search strategy in collaboration with an information specialist (FB; see Acknowledgments). Search strategies for each database are shown in online Appendix 1. We reviewed search strategies with all authors to ensure they were inclusive of relevant preference elicitation methods that might be used for values clarification. We conducted hand searches by reviewing articles that cited the previous version of these standards (values clarification chapter) or a previous systematic review of values clarification methods.

### Study Records: Data Management

We managed data with Covidence (Melbourne, Australia), reviewing data records at regular team meetings.

### Study Records: Selection Process

Two independent reviewers (SC, MM, TP, CR, CR-B) screened titles and abstracts to assess potential relevance, with a third reviewer adjudicating discrepancies and discussions of questions and points of disagreement at regular team meetings. Two independent reviewers then reviewed the full text of all articles deemed potentially relevant based on their title and abstract. Discrepancies in inclusion and exclusion at full text were adjudicated through team discussions at regular meetings until we reached consensus.

### Study Records: Data Collection Process

Two independent, trained research team members (SC, MM, TP, CR, CR-B) extracted data from each article using a standardized and pilot-tested data extraction form based on a previous form^17^ and adapted to this review. We resolved disagreements through discussion until consensus was reached. We contacted authors to collect any needed data that they did not report or were unable to report in their publication.

### Data Items

Regarding study participants, we recorded the sample size for control and intervention groups along with basic inclusion and exclusion criteria and whether or not they were making the actual decision or if the study was hypothetical. We defined a hypothetical scenario as one in which people are asked (explicitly or implicitly) to imagine that they are in a certain situation or facing a certain decision. We defined a real scenario as one in which people are facing a decision (e.g., because they have received a diagnosis) or are members of a population likely to face the decision in the near term (e.g., parents of children eligible to receive vaccines within the coming months).

Regarding interventions, we recorded the type of explicit values clarification method as listed in Table 2. We also recorded specific characteristics of each values clarification method, namely, whether it explicitly requires the user to engage with tradeoffs, whether it explicitly shows the user the correspondence between their options and what they value, and which, if any, theoretical or conceptual framework underpins it. Where relevant, we recorded whether a variable was collected via self-report, meaning whether responses were completed by participants themselves or by independent researchers based on direct observation, including coded qualitative data.

For comparators (controls), we recorded whether the comparator was no values clarification method or an implicit method and treated both as equivalent controls. The Cochrane review of patient decision aids specifies that all patient decision aids must contain implicit values clarification methods at minimum,^14^ and it is accordingly rare to have patient decision aids that do not present potential benefits and harms of options in organized ways. In other words, in the context of patient decision aids, there is no meaningful distinction between implicit methods and no values clarification. The different terminology is simply a function of how authors choose to name their control. We also recorded studies that compared different types of explicit values clarification methods to each other.

### Outcomes

Whenever such data were available, we extracted data regarding values congruence (i.e., the extent to which choices aligned with stated values) as our primary outcome, as well as secondary outcomes: decision readiness (worry, decision uncertainty, decision making preparation, knowledge), decisional conflict (measured with a version of the Decisional Conflict Scale and/or its subscales^46^), decision made, and postdecision and postimplementation health and well-being (decisional regret, longer-term health outcomes). Following data extraction by pairs of trained reviewers (SC, MM, TP, CR, CR-B), 3 authors (HOW, SCD, JJ) mapped all outcomes into broad outcome groups: worry (including perceived risk), decision uncertainty (not including decisional conflict), decisional conflict (decisional conflict scale or any subscales), decision making preparation (including self-efficacy for decision making), beliefs (including beliefs about the condition or underlying decision structure), knowledge, values (including reported utilities), shared decision making (i.e., the extent to which shared decision making occurred or not), effects on communication (including quality, length, or existence of communication), satisfaction with care, preferences (i.e., preferences expressed), decision (choice made and implemented) or decisional intent (choice intended or made and not yet implemented), values congruence, informed decision making (i.e., the extent to which someone made an evidence-informed, values-congruent, behaviorally implemented decision^9,47^), postdecision feelings (including satisfaction, regret), postdecision health, and user assessment of the intervention (including acceptability, satisfaction, perceived balance). We conducted meta-analyses on primary outcome values congruence and secondary outcome decisional conflict, as these outcomes had sufficient studies to do so.

### Risk of Bias in Individual Studies

Independent, trained research team members assessed risk of bias for each study using methods as defined in the Cochrane handbook, section 8.5.^48^ We conducted quantitative data syntheses with and without studies identified as being at high risk of bias to determine the sensitivity of overall findings to these studies.

### Data Synthesis

We synthesized frequency-based results (e.g., how many values clarification methods reflect a given design) descriptively. To synthesize effects on outcomes, we pooled all experiments that evaluated a values clarification method against no values clarification method or an implicit method. For multiarmed studies in which the comparison of a decision aid with and without a values clarification method included an arm that was not relevant to our comparison of interest (e.g., an information booklet serving as a control condition in an evaluation of the decision aid), we ignored the third arm. For multiarmed studies containing 2 or more different values clarification methods and 1 arm of implicit values clarification or control, we considered each comparison of a values clarification method against implicit values clarification, meaning that each of the multiarmed studies included in this review contributed multiple comparisons to the pooled set.

To meta-analyze results for values congruence, we pooled results from 11 studies using risk differences and applying a random-effects model. Here, *risk differences* refers to differences between treatment and control arms regarding the risk of making a value-incongruent decision. We extracted dichotomous data indicating the frequency (i.e., number of events and sample size) of values-incongruent decisions. To meta-analyze results for decisional conflict, we pooled results using standardized mean differences applying a random-effects model. We extracted data on total scores on the Decisional Conflict Scale. We explored and reported consistency using Higgins *I*^2^, which offers a measure of statistical heterogeneity across pooled studies. Specifically, this statistic describes the percentage of total variation across studies that is due to heterogeneity rather than chance.^49^ When we included multiple comparisons from a single study in a meta-analysis, we conducted sensitivity analyses by restricting meta-analyses to 1 comparison per contributing study and meta-analyzing all possible combinations using a random-effects model. This allowed us to ascertain whether the overall Higgins *I*^2^ estimate might be influenced by the inclusion of multiple study arms from similar populations. We used the Cochrane risk-of-bias tool to assess study bias along 7 domains as well as to assess an overall risk of bias. Where data permitted, we conducted subgroup meta-analyses of different types of explicit values clarification methods and of explicit values clarification methods that do and do not contain specific design features already identified in previous work,^16^ namely, whether the method explicitly requires the user to engage with tradeoffs in any way, whether it explicitly provides the user with the implications of what they value, and which, if any, theoretical or conceptual framework underpins it. We used *P* = 0.05 as a threshold for statistical significance and conducted analyses in RevMan, version 5.4.

## Results

### Articles Identified

**Figure 1:**
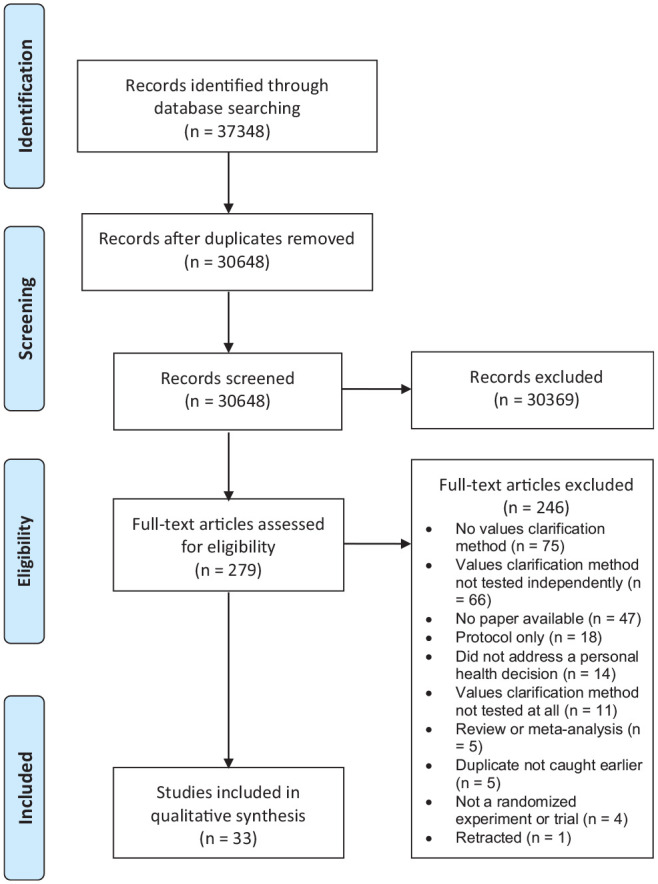
Preferred Reporting Items for Systematic Reviews and Meta-Analyses (PRISMA) diagram.

Out of 30,648 records screened at the title and abstract stage and 279 screened at the full-text stage, we identified 33 articles that met our inclusion criteria describing trials of 43 values clarification methods. Twenty-four of the articles were new articles identified in this update of the International Patient Decision Aids Standards (IPDAS). We excluded 2 of the articles previously included in the IPDAS values clarification chapter because they did not meet our revised inclusion criteria requiring randomized controlled trials and instead reported, for example, pre–post study designs. The PRISMA diagram of included articles is shown in Figure 1.

The decision context varied across studies. Out of the 43 included trials, 25 (58%) addressed treatment decisions, 9 (21%) screening decisions, 4 (9%) prevention, 3 (7%) genetic testing, and 2 (5%) diagnostic testing. Thirteen of the 43 trials (30%) centered on a yes/no decision to take an option or not, 18 (42%) a choice between 2 or more options, and 12 (28%) both a yes/no and a choice between 2 or more options. Most decisions (22/43, 51%) were real decisions, meaning that the person was making this decision in their actual life. The rest were hypothetical (18/43, 42%), or it was not entirely clear whether the decision was real or hypothetical (3/43, 3%). The most commonly reported outcomes were decisional conflict and/or its subscales (29/43, 67%), decision and/or decisional intentions (22/43, 51%), knowledge (13/43, 30%), and values congruence (12/43, 28%).

As shown in the overview of included studies in Table 3, there was substantial diversity in the types of values clarification methods used. Decision analysis or multicriteria decision analysis was the most commonly trialed method. Full study details are available in online Appendix 2.

**Table 3:** Study Details

Type(s) of Values Clarification Method(s)	Study	Population^a^	Decision	Summary of Findings^b^
Adaptive conjoint analysis	de Achaval et al., 2012^50^	*n* = 208 people with knee osteoarthritis	Whether to receive medication and therapy or total knee arthroplasty	Values clarification method decreased **decisional conflict** and required more intense **cognitive involvement**.
Adaptive conjoint analysis	Fraenkel et al., 2007^51^	*n* = 87, age at least 60 years old, self-report of pain involving 1 or both knees on most days of the month	Choice between 5 treatments for knee pain	Values clarification method increased **self-confidence in** and **preparation for shared decision making** and increased arthritis **self-efficacy**.
Adaptive conjoint analysis	Hess et al., 2015^52^	*n* = 374 women aged 18 years or older with abnormal uterine bleeding and potential candidates for either surgical or medical treatment	Whether or not to be treated for abnormal uterine bleeding and, if yes, which treatment to undertake	Values clarification method did not reduce **decision regret** or improve **treatment satisfaction**.
Adaptive conjoint analysis	Hutyra et al., 2019^53^	*n* = 200 people between 18 and 35 years of age at risk for experiencing a first-time anterior shoulder dislocation	Operative or nonoperative treatment for first-time anterior shoulder dislocation	Values clarification method increased **alignment between patients’ treatment decisions and evidence-based recommendations**.
Adaptive conjoint analysis	Jayadevappa et al., 2015^54^	*n* = 743 people with newly diagnosed localized prostate cancer	Choice between 6 options for early stage prostate cancer	Values clarification method improved **satisfaction with care**, **satisfaction with decision**, reduced **regrets**, and **aligned treatment choice with risk category**.
Allocation of points	Witteman et al., 2020^55^	*n* = 817 adults asked to imagine they had been diagnosed with colon cancer	Choice between 2 hypothetical surgeries for colon cancer	Values clarification method (strategy 6b in article) increased **values congruence** and reduced **decisional conflict**.
Analytical hierarchy process	Myers, 2003^56^	*n* = 199 men aged 50–69 years with no personal history of prostate cancer/benign prostate hyperplasia	Whether or not to be screened for prostate cancer	Values clarification method decreased **rates of prostate cancer screening**. Race/ethnicity analyses showed African American men increased **screening** while white men decreased **screening**.
Analytical hierarchy process	Myers et al., 2005^57^	*n* = 242 African American men, 40–69 years of age and no history of prostate cancer	Whether or not to be screened for prostate cancer and, if yes, choice of method/extent of screening	Values clarification method increased **prostate cancer screening**.
Best–worst scaling	Shirk et al., 2017^58^	*n* = 122 men with incident localized prostate cancer	Choice between 3 options for incident localized prostate cancer	Values clarification method decreased **decisional conflict**.
Decision analysis^c^	Bekker et al., 2004^59^	*n* = 106 pregnant women receiving a screen-positive maternal serum screening result	Whether or not to have a prenatal diagnosis for Down syndrome	Values clarification method helped women make more **informed prenatal diagnosis decisions**.
Decision analysis^c^	Clancy et al., 1988^60^	*n* = 1,280 resident and faculty physicians unvaccinated against hepatitis B	Choice between 3 options to manage risk of hepatitis B	Values clarification method resulted in greater **action taking** (screening or vaccination).
Decision analysis^c^	Feldman-Stewart et al., 2012^61^	*n* = 156 people with newly diagnosed prostate cancer	Choice between more than 5 main options for early stage prostate cancer	Values clarification method increased **preparation for decision making** and decreased **decision regret**. **Decisional conflict** decreased with and without values clarification method.
Decision analysis^c^	Hopkin et al., 2019^62^	*n* = 349 adults asked to imagine that they had to choose a statin	Choice between 5 commonly used statins	Values clarification method reduced **decisional conflict** and increased levels of **preparation for decision making**.
Decision analysis^c^	Montgomery et al., 2003^63^	*n* = 217 adults aged 30–80 years with newly diagnosed hypertension	Whether or not to start drug therapy for hypertension	Values clarification method increased **knowledge** and reduced total **decisional conflict** by significantly reducing scores on **uninformed, unclear values and unsupported subscales** and somewhat reducing scores on **uncertainty subscale**. Values clarification method did not influence scores on **decision quality subscale**, nor did it change **state anxiety**, **decision intention**, or **ultimate decision**.
Decision analysis^c^	Montgomery et al., 2007^64^	*n* = 742 pregnant women with 1 previous lower-segment caesarean section	Choice of planned mode of delivery	Values clarification method reduced **decisional conflict** and increased **frequency of having a vaginal birth**.
Decision analysis^c^	Witteman et al., 2015^65^	*n* = 407 parents who make medical decisions for at least 1 child aged 6 months to 18 years and whose child had not yet received the flu vaccine	Whether their child would receive a vaccine against influenza this flu season	Values clarification method had no effect on **values congruence**. Values clarification method combined with best practices in risk communication increased **intentions to vaccinate**, particularly among participants who had not had their children vaccinated against influenza in the past 5 years.
Decision analysis^c^	Witteman et al., 2020^55,d^	*n* = 1,731 adults asked to imagine they had been diagnosed with colon cancer deciding between 2 treatment options	Choice between 2 hypothetical surgeries for colon cancer	Values clarification method (strategies 2a, 2a + 2b, 6c, 6b + 6c in article) increased **values congruence** and reduced **decisional conflict** when this was measured (strategies 6c, 6b + 6c in article).
Discrete choice experiment	Brenner et al., 2014^66^	*n* = 615 people between the ages of 50 and 75 years at average risk for colorectal cancer	Whether or not to be screened for colorectal cancer, and, if yes, which screening test to use	Values clarification method influenced **choice of most important screening test attribute** but did not affect **unlabeled test preference**, **values clarity**, or **intent to be screened**.
Discrete choice experiment	Pignone et al., 2012^67^	*n* = 104 adults aged 48–75 years at average risk for colon cancer	Whether or not to be screened for colorectal cancer and, if yes, which screening test to use	Values clarification method influenced **choice of most important attribute** but did not affect **values clarity**, **intent to be screened**, or **choice of unlabeled screening test**.
Discrete choice experiment	Pignone et al., 2013^68^	*n* = 604 men aged 50–70 years at average risk of prostate cancer	Whether or not to be screened for prostate cancer	Values clarification method slightly reduced choice of dying as the **most important attribute** and increased **unlabeled PSA-like screening option** but did not influence **intent to be screened**.
Open discussion	Au et al., 2012^69^	*n* = 306 people with chronic obstructive pulmonary disease	Preferences for end-of-life care	Values clarification method helped identify what mattered to patients regarding **end-of-life care** and **communication**. **Quality of communication** improved.
Open discussion	Epstein et al., 2018^70^	*n* = 99 people with advanced gastrointestinal cancer	Choice between options for end-of-life care	Values clarification method improved **communication** about future medical cancer care but had no effect on **decisional conflict** or **well-being** and increased **distress**.
Open discussion	Kennedy et al., 2002^71^	*n* = 894 women with uncomplicated menorrhagia	Choice between treatment options for menorrhagia	Values clarification method resulted in minimal improvements in **self-reported health status**, lower **use of a more invasive treatment**, higher **patient satisfaction**, more frequent **clinician perceptions of “longer than usual” consultations**, and lower overall **costs**. Providing information alone did not affect treatment choices.
Open discussion	Lerman et al., 1997^72^	*n* = 700 women aged 18–75 years who had had at least 1 first-degree relative with breast and/or ovarian cancer	Whether or not to provide a blood sample for *BRCA1* testing in the future	Values clarification method increased the **perceived importance of the limitations and risk of *BRCA1* testing** and decreased the **perceived importance of the benefits of *BRCA1* testing**. No effect of values clarification method on **intent to test**.
Open discussion	Matheis-Kraft et al., 1997^73^	*n* = 60 women over age 70 years with at least 1 family member or friend who might act as their proxy to make decisions about life-sustaining treatment	Preferences for care in case of decisional incapacity	Values clarification of method’s effectiveness or lack thereof depended on which statistic (κ or percent agreement) was used to measure **concordance between women and proxies**.
Pros and cons	Abhyankar et al., 2010^74^	*n* = 30 healthy women asked to imagine having been diagnosed with breast cancer, undergoing lumpectomy, and getting a suggestion for chemotherapy by their doctor	Choice between having standard adjuvant chemotherapy or taking part in a clinical trial testing a new chemotherapy for early stage breast cancer	Values clarification method resulted in more **use of personal values** when evaluating attributes of options, somewhat less **ambivalence**, and less **uncertainty** and did not change **preferred option**.
Pros and cons	O’Connor et al., 1999^75^	*n* = 201 women aged 50–69 years who had never used hormone therapy	Whether or not to take hormone replacement therapy after menopause	Values clarification method had no effect on **clarity of values**, **values congruence**, total **decisional conflict**, **other subscales of the Decisional Conflict Scale**, or **acceptability of intervention**.
Pros and cons	Paquin et al., 2021^76^	*n* = 1,000 people aged 18–44 years who were pregnant or whose partner was pregnant or planning to become pregnant in the next 2 years	Whether or not to use genomic sequencing to identify genetic variants in one’s child	Values clarification method decreased **parental beliefs against genomic sequencing**.
Pros and cons	Peinado et al., 2020^15^	*n* = 1,000 people aged 18–44 years who were pregnant or whose partner was pregnant or planning to become pregnant in the next 2 years	Whether or not to enroll their newborn child in a medical research study that would involve screening for genetic conditions	Values clarification method decreased **decisional regret** and increased **clarity of personal values** but had no effect on overall **decisional conflict** or on **intent to have one’s child tested**.
Pros and cons	Witteman et al., 2020^55^	*n* = 772 adults asked to imagine they had been diagnosed with colon cancer	Choice between 2 hypothetical surgeries for colon cancer	Values clarification method (strategy 4b in article) reduced **decisional conflict** but did not change **values congruence**.
Rating scales	Garvelink et al., 2014^77,d^	*n* = 271 healthy women	Whether or not to undergo fertility-preserving procedures prior to cancer treatment	Values clarification method had no effect on **knowledge** or **decisional conflict**.
Rating scales	Kuppermann et al., 2014^78^	*n* = 710 pregnant women who had not yet undergone screening or diagnostic testing for fetal aneuploidy in the current pregnancy	Whether or not to have any screening or diagnostic testing for fetal aneuploidy; if screening or testing is desired, whether to start with screening or with invasive diagnostic testing; and which specific screening and/or diagnostic test(s) to undergo	Values clarification method increased patient **knowledge** and resulted in **less invasive prenatal test use** and more **informed choices**. Values clarification method did not change **decisional conflict** or **decisional regret**.
Rating scales (with and without decision-analytic summary)	Feldman-Stewart et al., 2006^79^	*n* = 90 male volunteers asked to imagine that they had just been diagnosed with early stage prostate cancer	Choice between 4 options for early stage prostate cancer	**Participants preferred** values clarification method with decision-analytic summary over values clarification method without summary and no values clarification method.
Rating scales	Witteman et al., 2020^55^	*n* = 785 adults asked to imagine they had been diagnosed with colon cancer	Choice between 2 hypothetical surgeries for colon cancer	Values clarification method (strategy 6a in article) reduced **decisional conflict** but did not change **values congruence**.
Rating scales + ranking	Brenner et al., 2014^66^	*n* = 614 people between the ages of 50 and 75 years at average risk for colorectal cancer	Whether or not to be screened for colorectal cancer, and, if yes, what screening test to use	Values clarification method increased the importance placed on risk reduction as an **important attribute** but did not affect **unlabeled test preference**, **values clarity**, or **intent to be screened**.
Rating scales + ranking	Pignone et al., 2012^67^	*n* = 104 adults aged 48–75 years at average risk for colon cancer	Whether or not to be screened for colorectal cancer, and, if yes, what screening test to use	Values clarification method influenced choice of **most important attribute** but did not affect **values clarity**, **intent to be screened**, or **choice of screening test**.
Rating scales + ranking	Pignone et al., 2013^68^	*n* = 609 men aged 50–70 years at average risk of prostate cancer	Whether or not to be screened for prostate cancer	Values clarification method increased the importance of dying (**attribute importance**) but did not influence **intent to be screened**.
Rating scales + ranking	Sheridan et al., 2010^80^	*n* = 137 men aged 45–80 years with no history of cardiovascular disease	Whether or not to initiate behaviors to prevent coronary heart disease and, if so, which behaviors	Values clarification method had no effect. **Decisional conflict**, **perceived values congruence**, and **self-efficacy** for health behaviors improved with and without values clarification. **Behavioral intentions** did not change.
Time tradeoff + rating scales	Frosch et al., 2008^81^	*n* = 611 men older than 50 years	Whether or not to be screened for prostate cancer	Values clarification method increased cancer **knowledge** scores and decreased **decisional conflict**.

PSA, prostate-specific antigen.

a *n* is given for the study as a whole. See supplementary appendix for further details about each study.

b Outcomes are in bold.

c *Decision analysis* or *multicriteria decision analysis* is an umbrella term. It encompasses some of the other, more specific categories (e.g., discrete-choice experiments, best–worst scaling). Throughout the article, when applicable, we use the more specific, narrower categories. Otherwise, we use the umbrella term *multicriteria decision analysis* or, for brevity in figures, *decision analysis*.

d Garvelink et al.^77^ and Witteman et al.^55^ each reported multiple experiments testing values clarification methods that did not differ in type or in outcomes. Pooled results are therefore presented here.

### Quality Assessment

Overall study quality was acceptable, with the majority of studies at low risk of bias on most elements. Eight studies were deemed to be at high risk of bias on 1 element, with the majority in Blinding of Participants and Personnel (Performance Bias). Eighteen additional studies were deemed unclear on this element. Blinding of Outcome Assessment (Detection Bias) was the next most common source of potential bias, with 1 study at high risk of bias and 20 more unclear. Full details of risk of bias assessments are available in online Appendix 3.

### Values Congruence

**Figure 2:**
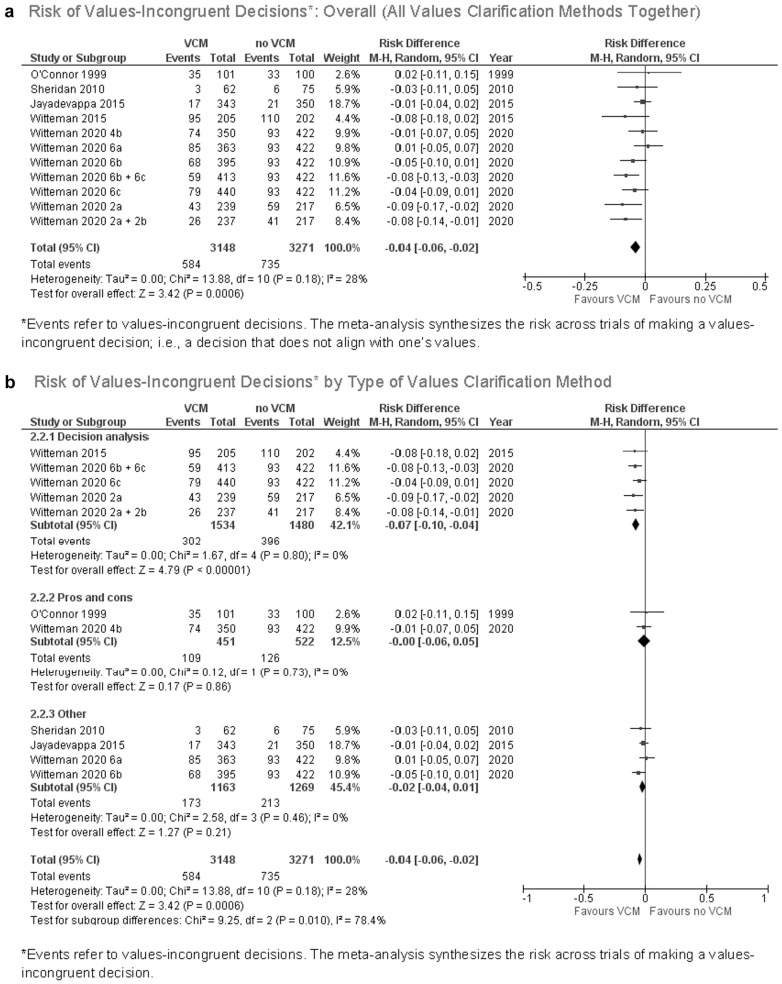
(a) Risk of values-incongruent decisions: overall (all values clarification methods together). (b) Risk of values-incongruent decisions by type of values clarification method.

As shown in Figure 2a, included explicit values clarification methods, as a group, increased values congruence, meaning people making decisions that aligned with their stated values. Eleven out of 43 trials (26%) reported the number of people who made values-congruent or values-incongruent decisions. The pooled risk difference of making a values-incongruent decision when using one of the trialed values clarification methods was –0.04 (95% confidence interval [CI], –0.06 to –0.02; *P* < 0.001). The *I* ^2^ of 28% indicates a low level of statistical heterogeneity.^82^ This estimate was robust to the inclusion and exclusion of multiple comparisons from a single study (see online Appendix 3, Suppl. Figures S10–S20).

Figure 2b shows a statistically significant subgroup difference by type of values clarification method. The results suggest that decision analysis is more likely to encourage values-congruent decisions compared to other explicit values clarification methods within this set of trials (χ^2^ = 9.25, *P* = 0.01). The results show no significant subgroup differences by whether the method explicitly requires the user to engage with tradeoffs in any way, whether it explicitly provides the user with the implications of what they value, or whether the method is underpinned by a formal theoretical or conceptual framework (see online Appendix 3). There were no studies in this analysis with a high risk of bias.

### Decisional Conflict

**Figure 3:**
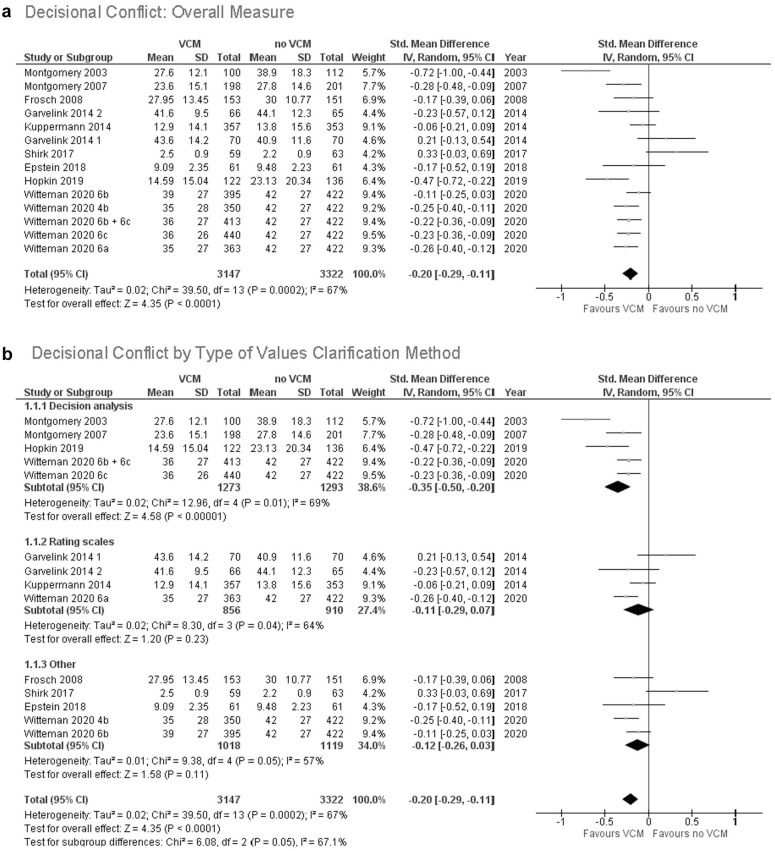
(a) Decisional conflict: overall measure. (b) Decisional conflict by type of values clarification method.

As shown in Figure 3a, explicit values clarification methods decrease decisional conflict. For the 14 of 43 (33%) trials for which we had complete data, the pooled standardized mean difference for decisional conflict was –0.20 (95% CI, –0.29 to –0.11; *P* < 0.001). The *I* ^2^ of 67% represents moderate to high statistical heterogeneity. This estimate was similar with inclusion and exclusion of multiple comparisons from a single study (see online Appendix 3, Suppl. Figures S22–S26). Figure 3b shows there was no significant subgroup difference by type of values clarification method (χ^2^ = 6.08, *P* = 0.05). We found no significant subgroup differences by tradeoffs, implications, theory, or risk of bias (see online Appendix 3).

### Head-to-Head Evaluations of Values Clarification Methods

The 5 studies that compared values clarification methods to each other reported findings that align with the findings of our meta-analyses. Methods that provided users with explicit feedback regarding how the decision options align with their stated values led to somewhat better outcomes, including greater values congruence.^55^ When asked to compare methods to each other, study participants also preferred a values clarification method that explicitly showed them how the decision options align with their stated values.^79^ Different values clarification methods yielded different patterns of attribute importance.^66–68^ Brief summaries of each study are available in online Appendix 3.

## Discussion

Overall, our systematic review and meta-analyses confirm that explicit values clarification methods improve decision outcomes, notably by increasing values congruence and decreasing decisional conflict. Patient decision aids should include an explicit values clarification method.

While the best explicit values clarification method may depend on context—for example, urgent v. routine care or the extent to which a decision has a clear set of decision attributes—our analyses suggest that patient decision aid developers may wish to consider methods that draw on multicriteria decision analysis. The apparent advantages of such methods shown in our analyses may reflect similarities between the process and the outcome. In other words, increased values congruence yielded by decision-analytic methods may be a function of the ways in which such methods transparently show people how their options align with their stated values. We also caution that when these methods use prespecified attributes, there might not be the flexibility for users to add new attributes, highlighting the importance of research to inform attribute selection. We acknowledge that some researchers have argued that health professionals having an unhurried, high-quality conversation with patients may be a preferred approach for at least some patients, especially when decision attributes are many and varied. However, in this systematic review, trials of Open Discussion values clarification methods did not demonstrate strong results, suggesting that such an ideal may be difficult to achieve.

To advance further knowledge on the merits and pitfalls of different values clarification methods, we recommend that authors of future trials of values clarification methods report 4 outcomes: decisional conflict, decision or decision intention, values congruence, and decisional regret. When possible, authors should make use of validated scales that have good psychometric properties and are commonly reported, as this facilitates evidence synthesis.

Decisional conflict should be assessed before people make the decision, using a version of the Decisional Conflict Scale.^46,83^ Decisions or decision intentions should be assessed when the decision is made.

Values congruence should be assessed once the decision is made. We acknowledge that including values congruence as an outcome brings both measurement and conceptual issues. Measurement issues exist because there are disagreements about how to measure what matters to people (or, indeed, whether it is conceptually possible to do so) and compare such measures to what people choose.^84^ Values congruence should not be measured using the values clarity subscale of the Decisional Conflict Scale, as this subscale measures perceived values clarity, not values congruence.^55^ Further research is required to determine whether measuring values congruence might introduce bias or otherwise negatively influence decision making.

Decisional regret should be assessed with a version of the Decisional Regret Scale^85,86^ after people make the decision, ideally with a sufficiently long delay so that longer-term effects can be captured. This scale, whose items include, “I would go for the same choice if I had to do it over again,” assesses how people feel about the decision itself, not the decision process. An included study in this review showed that a values clarification method reduced decisional regret but only after a year had passed following implementation of the decision.^61^

For all 4 measures, authors should clearly report sample mean and sample standard deviation for continuous measures, numbers in each category for categorical measures, and sample size per study arm in all cases. Finally, we recommend that patient decision aid developers explain the rationale for their choice of values clarification method.

Our study has 4 main limitations. First, the included data were of moderate quality. Although this review includes many robust trials, the included studies often measured different outcomes or the same outcomes in different ways, there were missing data in some studies, some studies had high risk of bias (often because it was not possible to prevent study participants from ascertaining the study arm to which they were assigned), and some of our meta-analyses had high heterogeneity. Together, these issues suggest a degree of caution in our conclusions. Second, we did not distinguish between subtypes of values clarification methods. For example, different adaptive conjoint analysis exercises may be very different from each other, as might open discussions or many other values clarification methods we grouped together, particularly those we grouped under the broad umbrella term of *multicriteria decision analysis*. Indeed, the values clarification methods used and trialed may simply reflect authors’ interests and expertise. The selection may also reflect views about whether it is preferable to invite users to explicitly consider individual attributes (e.g., rating scales or multicriteria decision analysis) or to consider options more holistically (e.g., discrete-choice experiments or adaptive conjoint analysis). Given the breadth of methods available, further comparative effectiveness research is needed to conclusively determine the superiority of any given method. Third, although assessment of values generally occurred following provision of information about options and attributes, we were unable to determine whether all instances of improved values congruence reflected informed values, as not all trials measured knowledge. Fourth and finally, our primary findings were heavily influenced by studies conducted with relatively homogeneous populations making hypothetical decisions. Although our sensitivity analyses suggested no differences between studies in real and hypothetical contexts, we nonetheless believe further study is needed in more diverse populations making real decisions before drawing firmer conclusions.

Our study also has 3 main strengths. First, we catalog definitions and resources regarding values clarification methods, as well as recommended outcomes to report in studies. In doing so, we hope to offer more clarity and structure to a literature that can be confusing to navigate, particularly for those who are newer to developing patient decision aids. Second, we begin to answer a core question that commonly arises when developing a patient decision aid: when including a values clarification method, which type of method should one use? Third and finally, we used rigorous methods and an expansive, systematic search. By conducting a systematic review, we reduced our likelihood of missing relevant studies. By including meta-analyses, we offer stronger findings and recommendations than would be possible without pooling data across multiple studies.

In conclusion, particularly in contexts in which people may make health decisions unaligned with what matters to them, patient decision aids should include an explicit values clarification method. Patient decision aid developers may wish to consider the potential advantages of multicriteria decision analysis. Future research should further investigate which methods lead to the best outcomes across or within particular decisions, populations, and settings. Authors of randomized controlled trials of explicit values clarification methods should report decisional conflict, decision made, values congruence, and decisional regret.

## Supplemental Material

sj-doc-1-mdm-10.1177_0272989X211037946 – Supplemental material for Clarifying Values: An Updated and Expanded Systematic Review and Meta-Analysis Click here for additional data file. Supplemental material, sj-doc-1-mdm-10.1177_0272989X211037946 for Clarifying Values: An Updated and Expanded Systematic Review and Meta-Analysis by Holly O. Witteman, Ruth Ndjaboue, Gratianne Vaisson, Selma Chipenda Dansokho, Bob Arnold, John F. P. Bridges, Sandrine Comeau, Angela Fagerlin, Teresa Gavaruzzi, Melina Marcoux, Arwen Pieterse, Michael Pignone, Thierry Provencher, Charles Racine, Dean Regier, Charlotte Rochefort-Brihay, Praveen Thokala, Marieke Weernink, Douglas B. White, Celia E. Wills and Jesse Jansen in Medical Decision Making

sj-pdf-1-mdm-10.1177_0272989X211037946 – Supplemental material for Clarifying Values: An Updated and Expanded Systematic Review and Meta-Analysis Click here for additional data file. Supplemental material, sj-pdf-1-mdm-10.1177_0272989X211037946 for Clarifying Values: An Updated and Expanded Systematic Review and Meta-Analysis by Holly O. Witteman, Ruth Ndjaboue, Gratianne Vaisson, Selma Chipenda Dansokho, Bob Arnold, John F. P. Bridges, Sandrine Comeau, Angela Fagerlin, Teresa Gavaruzzi, Melina Marcoux, Arwen Pieterse, Michael Pignone, Thierry Provencher, Charles Racine, Dean Regier, Charlotte Rochefort-Brihay, Praveen Thokala, Marieke Weernink, Douglas B. White, Celia E. Wills and Jesse Jansen in Medical Decision Making

sj-pdf-2-mdm-10.1177_0272989X211037946 – Supplemental material for Clarifying Values: An Updated and Expanded Systematic Review and Meta-Analysis Click here for additional data file. Supplemental material, sj-pdf-2-mdm-10.1177_0272989X211037946 for Clarifying Values: An Updated and Expanded Systematic Review and Meta-Analysis by Holly O. Witteman, Ruth Ndjaboue, Gratianne Vaisson, Selma Chipenda Dansokho, Bob Arnold, John F. P. Bridges, Sandrine Comeau, Angela Fagerlin, Teresa Gavaruzzi, Melina Marcoux, Arwen Pieterse, Michael Pignone, Thierry Provencher, Charles Racine, Dean Regier, Charlotte Rochefort-Brihay, Praveen Thokala, Marieke Weernink, Douglas B. White, Celia E. Wills and Jesse Jansen in Medical Decision Making

sj-xlsx-1-mdm-10.1177_0272989X211037946 – Supplemental material for Clarifying Values: An Updated and Expanded Systematic Review and Meta-Analysis Click here for additional data file. Supplemental material, sj-xlsx-1-mdm-10.1177_0272989X211037946 for Clarifying Values: An Updated and Expanded Systematic Review and Meta-Analysis by Holly O. Witteman, Ruth Ndjaboue, Gratianne Vaisson, Selma Chipenda Dansokho, Bob Arnold, John F. P. Bridges, Sandrine Comeau, Angela Fagerlin, Teresa Gavaruzzi, Melina Marcoux, Arwen Pieterse, Michael Pignone, Thierry Provencher, Charles Racine, Dean Regier, Charlotte Rochefort-Brihay, Praveen Thokala, Marieke Weernink, Douglas B. White, Celia E. Wills and Jesse Jansen in Medical Decision Making
