# Supplementary material for: Clarifying Values: An Updated and Expanded Systematic Review and Meta-Analysis
Source: Med Decis Making. 2021 Sep 25;41(7):801–20. doi: 10.1177/0272989X211037946 (PMC8482297; doi:10.1177/0272989X211037946)
Supplement: sj-pdf-1-mdm-10.1177_0272989X211037946 – Supplemental material for Clarifying Values: An Updated and Expanded Systematic Review and Meta-Analysis [file sj-pdf-1-mdm-10.1177_0272989X211037946.pdf]

Online Appendix 1: Search Strategies

**#1**	((value? OR patient preference? OR treatment preference?) adj5 (clarif* OR elicit*)).ti,ab,kw
**#2**	(Decision Making/ AND Social Values/)
**#3**	MCDA.ti,ab
**#4**	analytical hierarchy process.ti,ab,kw
**#5**	best-worst scaling.ti,ab,kw
**#6**	((conjoint OR decision) adj3 analysis):ti,ab,kw
**#7**	data envelopment analysis.ti,ab,kw
**#8**	Decision conferencing.ti,ab,kw
**#9**	Decision models.ti,ab,kw
**#10**	direct rating.ti,ab,kw
**#11**	points allocation.ti,ab,kw
**#12**	discrete choice experiment.ti,ab ,kw
**#13**	("dominance-based" adj3 approach*).ti,ab,kw
**#14**	EVIDEM framework.ti,ab,kw
**#15**	(geometrical analysis for interactive aid OR GAIA).ti,ab,kw
**#16**	MACBETH.ti,ab,kw
**#17**	("Measuring Attractiveness" adj4 "Categorical Based Evaluation TecHnique").ti,ab,kw
**#18**	Multi-Attribute Global Inference of Quality.ti,ab,kw
**#19**	(("Multiple attribute" OR "multiple criteria" OR multiattribute) adj2 (utility OR theory OR analysis)).ti,ab,kw
**#20**	(MAUT OR MAVT OR MCUA OR MCA).ti,ab,kw
**#21**	("Novel approach to imprecise assessment and decision environments" OR NAIADE).ti,ab,kw
**#22**	ORESTE.ti,ab,kw
**#23**	Pairwise comparisons.ti,ab,kw
**#24**	PAPRIKA.ti,ab,kw
**#25**	Pairwise RanKings.ti,ab,kw
**#26**	PROMETHEE.ti,ab,kw
**#27**	Preference Ranking Organization Method for Enrichment of Evaluations.ti,ab,kw
**#28**	QUALItative FLEXible.ti,ab,kw
**#29**	Simple Multi Attribute Rating Technique.ti,ab,kw
**#30**	SMART.ti,ab,kw
**#31**	Standard gamble.ti,ab,kw
**#32**	Swing weighting.ti,ab,kw
**#33**	TOPSIS.ti,ab,kw

#34 Technique for Order Preference by Similarity to the Ideal Solution.ti,ab,kw
#35 (Time tradeoff OR time tradeoff).ti,ab ,kw
#36 Value function methods.ti,ab,kw
#37 Valutazione delle Tecnologie Sanitarie.ti,ab,kw
#38 VDA.ti,ab,kw
#39 VTS.ti,ab,kw
#40 verbal decision analysis.ti,ab,kw
#41 visual analog scale.ti,ab,kw
#42 willingness-to-pay.ti,ab,kw
#43 ((Scoring OR weighting) adj1 methods).ti,ab,kw
#44 REGIME.ti,ab,kw
#45 (scal* adj2 (methods OR Natural OR Constructed OR Objective)).ti,ab,kw
#46 OR/3-45
#47 Randomized Controlled Trials as Topic/
#48 randomized controlled trial/
#49 Random Allocation/
#50 Double Blind Method/
#51 Single Blind Method/
#52 clinical trial/
#53 clinical trial, phase i.pt
#54 clinical trial, phase ii.pt
#55 clinical trial, phase iii.pt
#56 clinical trial, phase iv.pt
#57 controlled clinical trial.pt
#58 randomized controlled trial.pt
#59 multicenter study.pt
#60 clinical trial.pt
#61 exp Clinical Trials as topic/
#62 (clinical adj trial$).tw
#63 ((singl$ or doubl$ or treb$ or tripl$) adj (blind$3 or mask$3)).tw
#64 PLACEBOS/
#65 placebo$.tw
#66 randomly allocated.tw

#67 (allocated adj2 random$).tw
#68 OR/47-67
#69 (#1 OR #2 OR #46) AND #68
#70 #70 NOT (animals/ NOT humans/)

**#1**	((value* OR "patient preference" OR "treatment preferences") NEAR/5 (clarif* OR elicit*)):ti,ab,kw
**#2**	'decision support system'/de OR 'patient decision making'/de
**#3**	MCDA:ti,ab,kw
**#4**	"analytical hierarchy process":ti,ab,kw
**#5**	"best-worst scaling":ti,ab,kw
**#6**	((conjoint OR decision) NEAR/3 analysis):ti,ab,kw
**#7**	"data envelopment analysis":ti,ab,kw
**#8**	"Decision conferencing":ti,ab,kw
**#9**	"Decision models":ti,ab,kw
**#10**	"direct rating":ti,ab,kw
**#11**	"points allocation":ti,ab,kw
**#12**	"discrete choice experiment":ti,ab,kw
**#13**	("dominance-based" NEAR/3 approach*):ti,ab,kw
**#14**	"EVIDEM framework":ti,ab,kw
**#15**	"geometrical analysis for interactive aid OR GAIA":ti,ab,kw
**#16**	MACBETH:ti,ab,kw
**#17**	("Measuring Attractiveness" NEAR/4 "Categorical Based Evaluation TecHnique"):ti,ab,kw
**#18**	"Multi-Attribute Global Inference of Quality":ti,ab,kw
**#19**	(("Multiple attribute" OR "multiple criteria" OR multiattribute) NEAR/2 (utility OR theory OR analysis)):ti,ab,kw
**#20**	(MAUT OR MAVT OR MCUA OR MCA):ti,ab,kw
**#21**	("Novel approach to imprecise assessment and decision environments" OR NAIADE):ti,ab,kw
**#22**	ORESTE:ti,ab,kw
**#23**	"Pairwise comparisons":ti,ab,kw
**#24**	PAPRIKA:ti,ab,kw
**#25**	"Pairwise RanKings":ti,ab,kw
**#26**	PROMETHEE:ti,ab,kw
**#27**	"Preference Ranking Organization Method for Enrichment of Evaluations":ti,ab,kw
**#28**	"QUALItative FLEXible":ti,ab,kw
**#29**	"Simple Multi Attribute Rating Technique":ti,ab,kw
**#30**	SMART:ti,ab,kw
**#31**	"Standard gamble":ti,ab,kw
**#32**	"Swing weighting":ti,ab,kw
**#33**	TOPSIS:ti,ab,kw

**#34**	"Technique for Order Preference by Similarity to the Ideal Solution":ti,ab,kw
**#35**	("Time tradeoff" OR "time tradeoff"):ti,ab,kw
**#36**	"Value function methods":ti,ab,kw
**#37**	"Valutazione delle Tecnologie Sanitarie":ti,ab,kw
**#38**	VDA:ti,ab,kw
**#39**	VTS:ti,ab,kw
**#40**	"verbal decision analysis":ti,ab,kw
**#41**	"visual analog scale":ti,ab,kw
**#42**	"willingness-to-pay":ti,ab,kw
**#43**	((Scoring OR weighting) NEAR/1 methods):ti,ab,kw
**#44**	REGIME:ti,ab,kw
**#45**	(scal* NEAR/2 (methods OR Natural OR Constructed OR Objective)):ti,ab,kw
**#46**	#3 OR #4 OR #5 OR #6 OR #7 OR #8 OR #9 OR #10 OR #11 OR #12 OR #13 OR #14 OR #15 OR #16 OR #17 OR #18 OR #19 OR #20 OR [illegible]
**#47**	'clinical trial'/de
**#48**	'randomized controlled trial'/de
**#49**	'randomization'/de
**#50**	'single blind procedure'/de
**#51**	'double blind procedure'/de
**#52**	'crossover procedure'/de
**#53**	'placebo'/de OR 'prospective study'/de
**#54**	(randomi?ed NEXT/1 controlled NEXT/1 trial):ab,ti
**#55**	rct:ab,ti OR 'random allocation':ab,ti
**#56**	'randomly allocated':ab,ti OR 'allocated randomly':ab,ti
**#57**	(allocated NEAR/2 random):ab,ti
**#58**	((single OR double OR treble OR triple) NEXT/1 blind*):ab,ti
**#59**	placebo*:ab,ti
**#60**	#47 OR #48 OR #49 OR #50 OR #51 OR #52 OR #53 OR #54 OR #55 OR #56 OR #57 OR #58 OR #59
**#61**	(#1 OR #2 OR #46) AND #60
**#62**	#61 NOT (animal:de NOT human:de)
**#63**	[embase]/lim NOT ([embase]/lim AND [medline]/lim)

#1 ((value* OR "patient preference*" OR "treatment preference*") NEAR/5 (clarif* OR elicit*)):ti,ab
#2 ("Decision Making":kw AND "Social Values":kw)
#3 MCDA:ti,ab
#4 "analytical hierarchy process":ti,ab
#5 best-worst scaling:ti,ab
#6 ((conjoint OR decision) NEAR/3 analysis):ti,ab
#7 "Decision conferencing":ti,ab
#8 "Decision models":ti,ab
#9 "direct rating":ti,ab
#10 "points allocation":ti,ab
#11 "discrete choice experiment":ti,ab
#12 ("dominance-based" NEAR/3 approach*):ti,ab
#13 "Elimination and Choice Expressing Reality":ti,ab
#14 "EVIDEM framework":ti,ab
#15 "geometrical analysis for interactive aid" OR GAIA:ti,ab
#16 MACBETH:ti,ab
#17 ("Measuring Attractiveness" NEAR/4 "Categorical Based Evaluation TecHnique"):ti,ab
#18 "Multi-Attribute Global Inference of Quality":ti,ab
#19 "multiattribute objective function specification":ti,ab
#20 (("Multiple attribute" OR "multiple criteria" OR multiattribute) NEAR/2 (utility OR theory OR analysis)):ti,ab
#21 (MAUT OR MAVT OR MCUA OR MCA):ti,ab
#22 ("Novel approach to imprecise assessment and decision environments" OR NAIADE):ti,ab
#23 ORESTE:ti,ab
#24 "Pairwise comparisons":ti,ab
#25 PAPRIKA:ti,ab
#26 "Pairwise RanKings":ti,ab
#27 PROMETHEE:ti,ab
#28 "Preference Ranking Organization Method for Enrichment of Evaluations":ti,ab
#29 "QUALItative FLEXible":ti,ab
#30 "Simple Multi Attribute Rating Technique":ti,ab
#31 SMART:ti,ab
#32 "Standard gamble":ti,ab
#33 "Swing weighting":ti,ab

**#34**	TOPSIS:ti,ab
**#35**	"Technique for Order Preference by Similarity to the Ideal Solution":ti,ab
**#36**	("Time tradeoff" OR "time tradeoff"):ti,ab
**#37**	"Value function methods":ti,ab
**#38**	"Valutazione delle Tecnologie Sanitarie":ti,ab
**#39**	VDA:ti,ab
**#40**	VTS:ti,ab
**#41**	"verbal decision analysis":ti,ab
**#42**	"visual analog scale":ti,ab
**#43**	"willingness-to-pay":ti,ab
**#44**	scoring method*:ti,ab OR "weighting method*":ti,ab
**#45**	REGIME:ti,ab
**#46**	(scal* NEAR/2 (methods OR Natural OR Constructed OR Objective)):ti,ab
**#47**	{OR #3-#46}
**#48**	#1 OR #2 OR #47

**#1**	TS=(((value$ OR "patient preference$" OR "treatment preference$") NEAR/5 (clarif* OR elicit*)))
**#2**	TS=((conjoint OR decision) NEAR/3 analysis)
**#3**	TS=(MCDA)
**#4**	TS=("analytical hierarchy process")
**#5**	TS=("best-worst scaling")
**#6**	TS=("data envelopment analysis")
**#7**	TS=("Decision conferencing")
**#8**	TS=("Decision models")
**#9**	TS=("direct rating")
**#10**	TS=("points allocation")
**#11**	TS=("discrete choice experiment")
**#12**	TS=("dominance-based" NEAR/3 approach*)
**#13**	TS=("Elimination and Choice Expressing Reality")
**#14**	TS=("EVIDEM framework")
**#15**	TS=("geometrical analysis for interactive aid" OR GAIA)
**#16**	TS=(MACBETH)
**#17**	TS= ("Measuring Attractiveness" NEAR/4 "Categorical Based Evaluation TecHnique")
**#18**	TS=("Multi-Attribute Global Inference of Quality")
**#19**	TS=(("Multiple attribute" OR "multiple criteria" OR multiattribute) NEAR/2 (utility OR theory OR analysis))
**#20**	TS=(MAUT OR MAVT OR MCUA OR MCA)
**#21**	TS=("Novel approach to imprecise assessment and decision environments" OR NAIADE)
**#22**	TS=(ORESTE)
**#23**	TS=("Pairwise comparisons")
**#24**	TS=(PAPRIKA)
**#25**	TS=("Pairwise RanKings")
**#26**	TS=(PROMETHEE)
**#27**	TS=("Preference Ranking Organization Method for Enrichment of Evaluations")
**#28**	TS=("QUALItative FLEXible")
**#29**	TS=("Simple MultiAttribute Rating Technique")
**#30**	TS=(SMART)
**#31**	TS=("Standard gamble")
**#32**	TS=(TOPSIS)
**#33**	TS=("Technique for Order Preference by Similarity to the Ideal Solution")

**#34**	TS=("time tradeoff" OR "time tradeoff")
**#35**	TS=("Value function methods")
**#36**	TS=("Valutazione delle Tecnologie Sanitarie")
**#37**	TS=(VDA)
**#38**	TS=(VTS)
**#39**	TS=("verbal decision analysis")
**#40**	TS=("visual analog scale")
**#41**	TS=("willingness-to-pay")
**#42**	TS= ((Scoring OR weighinng) NEAR/1 methods)
**#43**	TS=(REGIME)
**#44**	TS=(scal* NEAR/2 (methods OR Natural OR Constructed OR Objective))
**#45**	#44 OR #43 OR #42 OR #41 OR #40 OR #39 OR #38 OR #37 OR #36 OR #35 OR #34 OR #33 OR #32 OR #31 OR #30 OR #29 OR #28 OR #27 OR #26 OR #25 OR #24 OR #23 OR #22 OR #21 OR #20 OR #19 OR #18 OR #17 OR #16 OR #15 OR #14 OR #13 OR #12 OR #11 OR #10 OR #9 OR #8 OR #7 OR #6 OR #5 OR #4 OR #3 OR #2
**#46**	"clinical trial"
**#47**	randomization
**#48**	"crossover procedure"
**#49**	placebo
**#50**	"prospective study"
**#51**	(randomi$ed NEAR/1 "controlled trial")
**#52**	rct
**#53**	(allocat* NEAR/2 random*)
**#54**	((single OR double OR treble OR triple) NEAR/1 blind*))
**#55**	#46 OR #47 OR #48 OR #49 OR #50 OR #51 OR #52 OR #53 #54
**#56**	(#1 OR #45) AND #55

**#1**	TI ((value# OR "patient preference#" OR "treatment preference#") N5 ((clarif* OR elicit*))
**#2**	AB ((value# OR "patient preference#" OR "treatment preference#") N5 ((clarif* OR elicit*))
**#3**	MH "Values Clarification"
**#4**	MM "Decision Support Techniques"
**#5**	TI ((conjoint OR decision) N3 analysis) OR AB ((conjoint OR decision) N3 analysis)
**#6**	TI MCDA OR AB MCDA
**#7**	TI "analytical hierarchy process" OR AB "analytical hierarchy process"
**#8**	TI "best-worst scaling" OR AB "best-worst scaling"
**#9**	TI "data envelopment analysis" OR AB "data envelopment analysis"
**#10**	TI "Decision conferencing" OR AB "Decision conferencing"
**#11**	TI "Decision models" OR AB "Decision models"
**#12**	TI "direct rating" OR AB "direct rating"
**#13**	TI "points allocation" OR AB "points allocation"
**#14**	TI "discrete choice experiment" OR AB "discrete choice experiment"
**#15**	TI ("dominance-based" N3 approach*) OR AB ("dominance-based" N3 approach*)
**#16**	TI "Elimination and Choice Expressing Reality" OR AB "Elimination and Choice Expressing Reality"
**#17**	TI "EVIDEM framework" OR AB "EVIDEM framework"
**#18**	TI "geometrical analysis for interactive aid" OR GAIA OR AB "geometrical analysis for interactive aid" OR GAIA
**#19**	TI MACBETH OR AB MACBETH
**#20**	TI ("Measuring Attractiveness" N4 "Categorical Based Evaluation TecHnique") OR AB ("Measuring Attractiveness" N4 "
**#21**	TI (("Multiple attribute" OR "multiple criteria" OR multiattribute) N2 (utility OR theory OR analysis)) OR AB (("Multiple
**#22**	TI (MAUT OR MAVT OR MCUA OR MCA) OR AB (MAUT OR MAVT OR MCUA OR MCA)
**#23**	TI ORESTE OR AB ORESTE
**#24**	TI "Pairwise comparisons" OR AB "Pairwise comparisons"
**#25**	TI PAPRIKA OR AB PAPRIKA
**#26**	TI "Pairwise RanKings" OR AB "Pairwise RanKings"
**#27**	TI SMART OR AB SMART
**#28**	TI "Standard gamble" OR AB "Standard gamble"
**#29**	TI "Swing weighting" OR AB "Swing weighting"
**#30**	TI TOPSIS OR AB TOPSIS
**#31**	TI "Technique for Order Preference by Similarity to the Ideal Solution" OR AB "Technique for Order Preference by Simi
**#32**	TI ("Time tradeoff" OR "time tradeoff") OR AB ("Time tradeoff" OR "time tradeoff")
**#33**	TI VDA OR AB VDA

#34 TI VTS OR AB VTS

#35 TI "visual analog scale" OR AB "visual analog scale"

#36 TI "willingness-to-pay" OR AB "willingness-to-pay"

#37 TI Scoring OR "weighting method#" OR AB Scoring OR "weighting method#"

#38 TI REGIME OR AB REGIME

#39 TI (scal* N2 (methods OR Natural OR Constructed OR Objective)) OR AB (scal* N2 (methods OR Natural OR Constructe

#40 (S5 OR S6 OR S7 OR S8 OR S9 OR S10 OR S11 OR S12 OR S13 OR S14 OR S15 OR S16 OR S17 OR S18 OR S19 OR S20 OR

#41 TX allocat* random*

#42 MH "Quantitative Studies"

#43 MH "Placebos"

#44 TX placebo*

#45 TX random* allocat*

#46 MH "Random Assignment"

#47 TX randomi* control* trial*

#48 TX ((singl* n1 blind*) OR (singl* n1 mask*))

#49 TX ((doubl* n1 blind*) OR (doubl* n1 mask*))

#50 TX ((tripl* n1 blind*) OR (tripl* n1 mask*))

#51 TX ((trebl* n1 blind*) or (trebl* n1 mask*))

#52 TX (clinic* n1 trial*)

#53 PT "Clinical trial"

#54 MH "Clinical Trials+"

#55 #52 OR #65

#56 (#1 OR #2 OR #3 OR #4 OR #40) AND #55
