# Supplementary material for: Clarifying Values: An Updated and Expanded Systematic Review and Meta-Analysis
Source: Med Decis Making. 2021 Sep 25;41(7):801–20. doi: 10.1177/0272989X211037946 (PMC8482297; doi:10.1177/0272989X211037946)

Online Appendix 3. Additional Results

This appendix is associated with Witteman and colleagues, 2021, Clarifying Values: An Updated and Expanded Systematic Review and Meta-Analysis.

Additional Meta-analytic Results

Figure S1. Risk of Values-Incongruent Decisions by Tradeoffs

This analysis compares the risk of values-incongruent decisions for values clarification methods that explicitly require the user to engage with tradeoffs in some way versus those that do not require such engagement.

	VCM		no VCM			Risk Difference	
**Study or Subgroup**	**Events**	**Total**	**Events**	**Total**	**Weight**	**M-H, Random, 95% CI**	**Year**
**2.2.1 Explicit tradeoffs**							
Sheridan 2010	3	62	6	75	5.9%	-0.03 [-0.11, 0.05]	2010
Jayadevappa 2015	17	343	21	350	18.7%	-0.01 [-0.04, 0.02]	2015
Witteman 2015	95	205	110	202	4.4%	-0.08 [-0.18, 0.02]	2015
Witteman 2020 6b	68	395	93	422	10.9%	-0.05 [-0.10, 0.01]	2020
Witteman 2020 2a	43	239	59	217	6.5%	-0.09 [-0.17, -0.02]	2020
Witteman 2020 2a + 2b	26	237	41	217	8.4%	-0.08 [-0.14, -0.01]	2020
Witteman 2020 6b + 6c	59	413	93	422	11.6%	-0.08 [-0.13, -0.03]	2020
**Subtotal (95% CI)**		**1894**		**1905**	**66.5%**	**-0.05 [-0.08, -0.02]**	
Total events	311		423				
Heterogeneity: Tau² = 0.00; Chi² = 10.41, df = 6 (P = 0.11); I² = 42%							
Test for overall effect: Z = 3.49 (P = 0.0005)							
**2.2.2 Not at all**							
O'Connor 1999	35	101	33	100	2.6%	0.02 [-0.11, 0.15]	1999
Witteman 2020 4b	74	350	93	422	9.9%	-0.01 [-0.07, 0.05]	2020
Witteman 2020 6a	85	363	93	422	9.8%	0.01 [-0.05, 0.07]	2020
Witteman 2020 6c	79	440	93	422	11.2%	-0.04 [-0.09, 0.01]	2020
**Subtotal (95% CI)**		**1254**		**1366**	**33.5%**	**-0.01 [-0.04, 0.02]**	
Total events	273		312				
Heterogeneity: Tau² = 0.00; Chi² = 2.06, df = 3 (P = 0.56); I² = 0%							
Test for overall effect: Z = 0.75 (P = 0.45)							
**Total (95% CI)**		**3148**		**3271**	**100.0%**	**-0.04 [-0.06, -0.02]**	
Total events	584		735				
Heterogeneity: Tau² = 0.00; Chi² = 13.88, df = 10 (P = 0.18); I² = 28%							
Test for overall effect: Z = 3.42 (P = 0.0006)							
Test for subgroup differences: Chi² = 3.47, df = 1 (P = 0.06), I² = 71.2%							

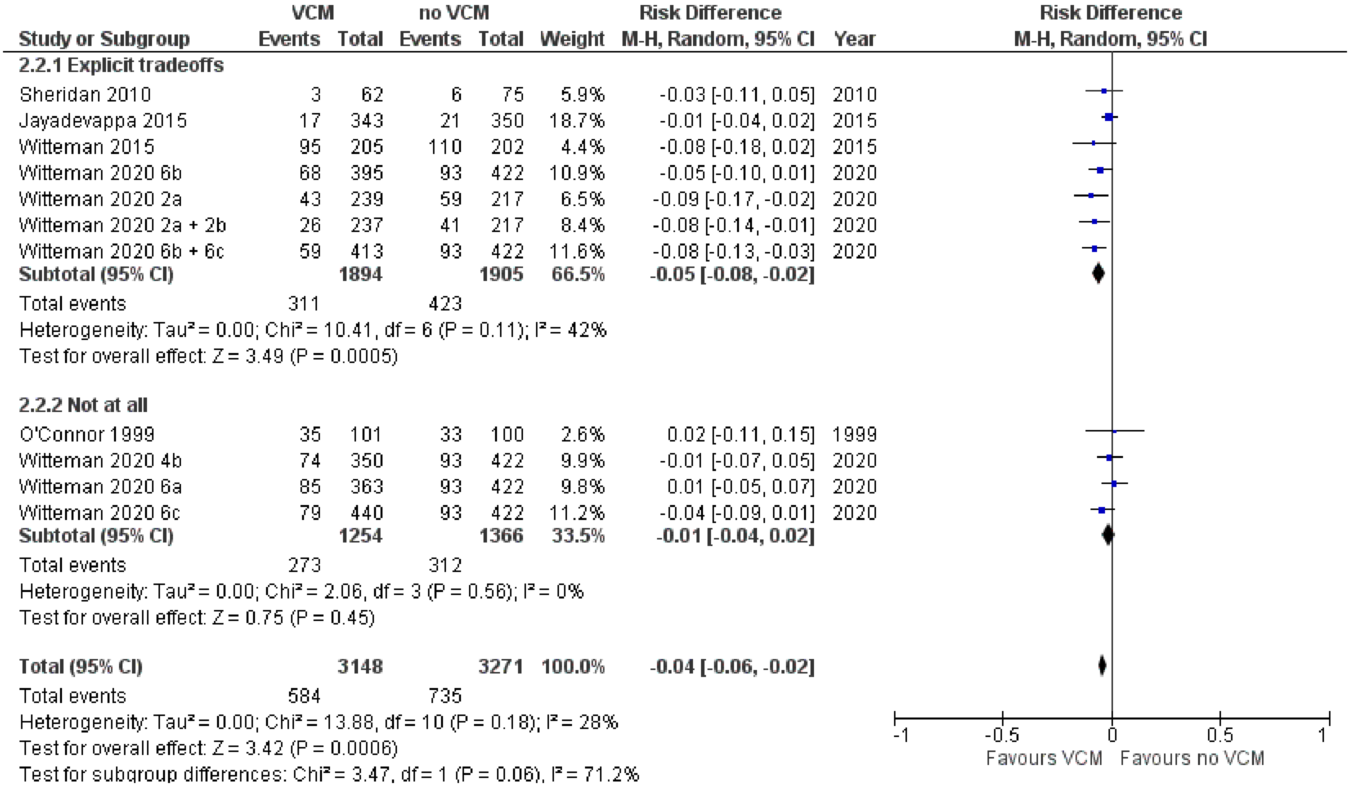

Figure S2. Risk of Values-Incongruent Decisions by Implications/Presentation of Results

This analysis compares the risk of values-incongruent decisions for values clarification methods that explicitly show the user with the implications of what they value (i.e., present the user with the ‘results’ of their values clarification process) versus those that do not show the user any such thing.

	VCM		no VCM			Risk Difference	
**Study or Subgroup**	**Events**	**Total**	**Events**	**Total**	**Weight**	**M-H, Random, 95% CI**	**Year**
**2.2.1 Presentation of results**							
Witteman 2015	95	205	110	202	4.4%	-0.08 [-0.18, 0.02]	2015
Jayadevappa 2015	17	343	21	350	18.7%	-0.01 [-0.04, 0.02]	2015
Witteman 2020 6b + 6c	59	413	93	422	11.6%	-0.08 [-0.13, -0.03]	2020
Witteman 2020 6c	79	440	93	422	11.2%	-0.04 [-0.09, 0.01]	2020
Witteman 2020 2a	43	239	59	217	6.5%	-0.09 [-0.17, -0.02]	2020
Witteman 2020 2a + 2b	26	237	41	217	8.4%	-0.08 [-0.14, -0.01]	2020
**Subtotal (95% CI)**		**1877**		**1830**	**60.8%**	**-0.06 [-0.09, -0.02]**	
Total events	319		417				
Heterogeneity: Tau² = 0.00; Chi² = 10.07, df = 5 (P = 0.07); I² = 50%							
Test for overall effect: Z = 3.25 (P = 0.001)							
**2.2.2 No presentation of results**							
O'Connor 1999	35	101	33	100	2.6%	0.02 [-0.11, 0.15]	1999
Sheridan 2010	3	62	6	75	5.9%	-0.03 [-0.11, 0.05]	2010
Witteman 2020 4b	74	350	93	422	9.9%	-0.01 [-0.07, 0.05]	2020
Witteman 2020 6a	85	363	93	422	9.8%	0.01 [-0.05, 0.07]	2020
Witteman 2020 6b	68	395	93	422	10.9%	-0.05 [-0.10, 0.01]	2020
**Subtotal (95% CI)**		**1271**		**1441**	**39.2%**	**-0.02 [-0.05, 0.01]**	
Total events	265		318				
Heterogeneity: Tau² = 0.00; Chi² = 2.81, df = 4 (P = 0.59); I² = 0%							
Test for overall effect: Z = 1.10 (P = 0.27)							
**Total (95% CI)**		**3148**		**3271**	**100.0%**	**-0.04 [-0.06, -0.02]**	
Total events	584		735				
Heterogeneity: Tau² = 0.00; Chi² = 13.88, df = 10 (P = 0.18); I² = 28%							
Test for overall effect: Z = 3.42 (P = 0.0006)							
Test for subgroup differences: Chi² = 2.92, df = 1 (P = 0.09), I² = 65.7%							

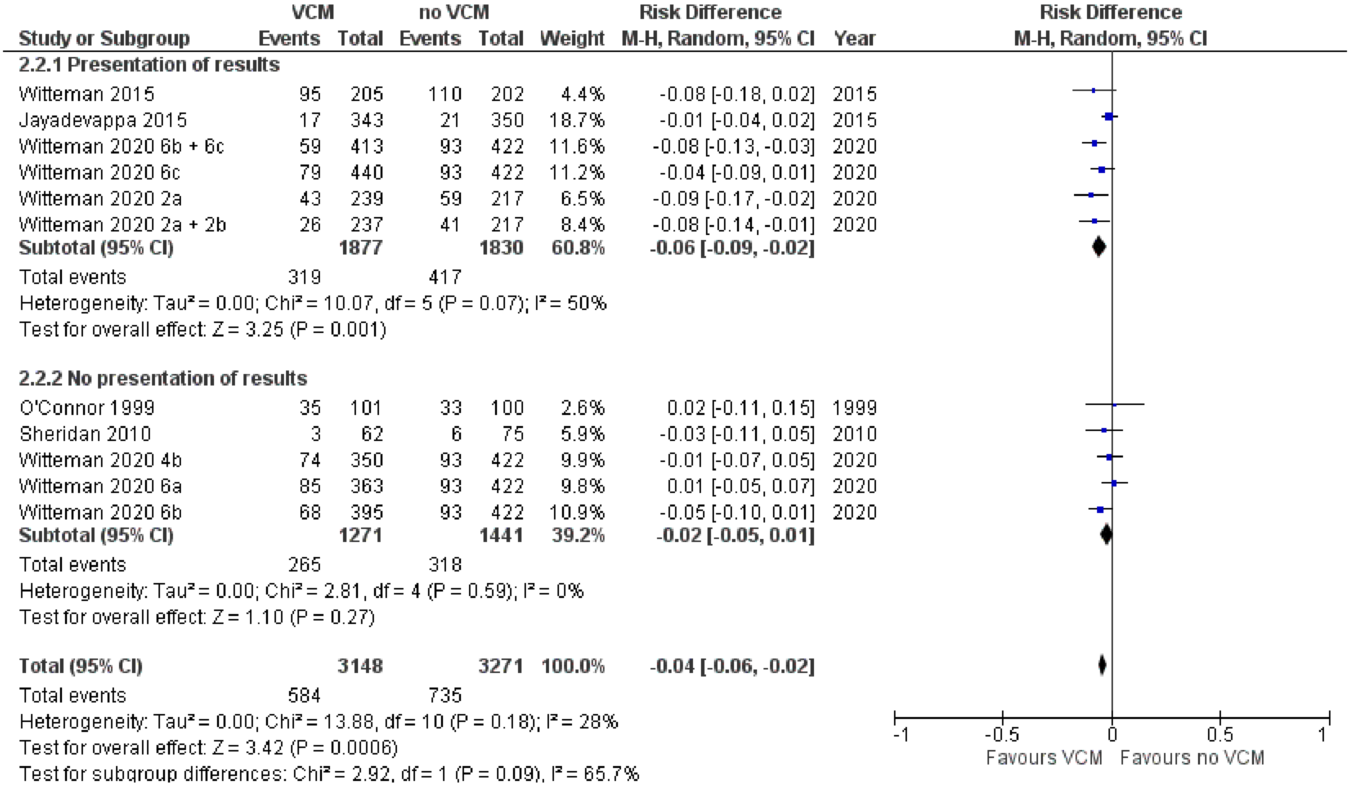

Figure S3. Risk of Values-Incongruent Decisions by Stated Use of a Theory/Framework

This analysis compares the risk of values-incongruent decisions for values clarification methods that are underpinned by a formal theory or conceptual framework versus those that are not.

	VCM		no VCM			Risk Difference	
**Study or Subgroup**	**Events**	**Total**	**Events**	**Total**	**Weight**	**M-H, Random, 95% CI**	**Year**
**2.2.1 Stated theory**							
Jayadevappa 2015	17	343	21	350	18.7%	-0.01 [-0.04, 0.02]	2015
**Subtotal (95% CI)**		**343**		**350**	**18.7%**	**-0.01 [-0.04, 0.02]**	
Total events	17		21				
Heterogeneity: Not applicable							
Test for overall effect: Z = 0.60 (P = 0.55)							
**2.2.2 No stated theory**							
O'Connor 1999	35	101	33	100	2.6%	0.02 [-0.11, 0.15]	1999
Sheridan 2010	3	62	6	75	5.9%	-0.03 [-0.11, 0.05]	2010
Witteman 2015	95	205	110	202	4.4%	-0.08 [-0.18, 0.02]	2015
Witteman 2020 2a	43	239	59	217	6.5%	-0.09 [-0.17, -0.02]	2020
Witteman 2020 2a + 2b	26	237	41	217	8.4%	-0.08 [-0.14, -0.01]	2020
Witteman 2020 4b	74	350	93	422	9.9%	-0.01 [-0.07, 0.05]	2020
Witteman 2020 6a	85	363	93	422	9.8%	0.01 [-0.05, 0.07]	2020
Witteman 2020 6b	68	395	93	422	10.9%	-0.05 [-0.10, 0.01]	2020
Witteman 2020 6b + 6c	59	413	93	422	11.6%	-0.08 [-0.13, -0.03]	2020
Witteman 2020 6c	79	440	93	422	11.2%	-0.04 [-0.09, 0.01]	2020
**Subtotal (95% CI)**		**2805**		**2921**	**81.3%**	**-0.04 [-0.07, -0.02]**	
Total events	567		714				
Heterogeneity: Tau² = 0.00; Chi² = 10.83, df = 9 (P = 0.29); I² = 17%							
Test for overall effect: Z = 3.79 (P = 0.0002)							
**Total (95% CI)**		**3148**		**3271**	**100.0%**	**-0.04 [-0.06, -0.02]**	
Total events	584		735				
Heterogeneity: Tau² = 0.00; Chi² = 13.88, df = 10 (P = 0.18); I² = 28%							
Test for overall effect: Z = 3.42 (P = 0.0006)							
Test for subgroup differences: Chi² = 2.64, df = 1 (P = 0.10), I² = 62.1%							

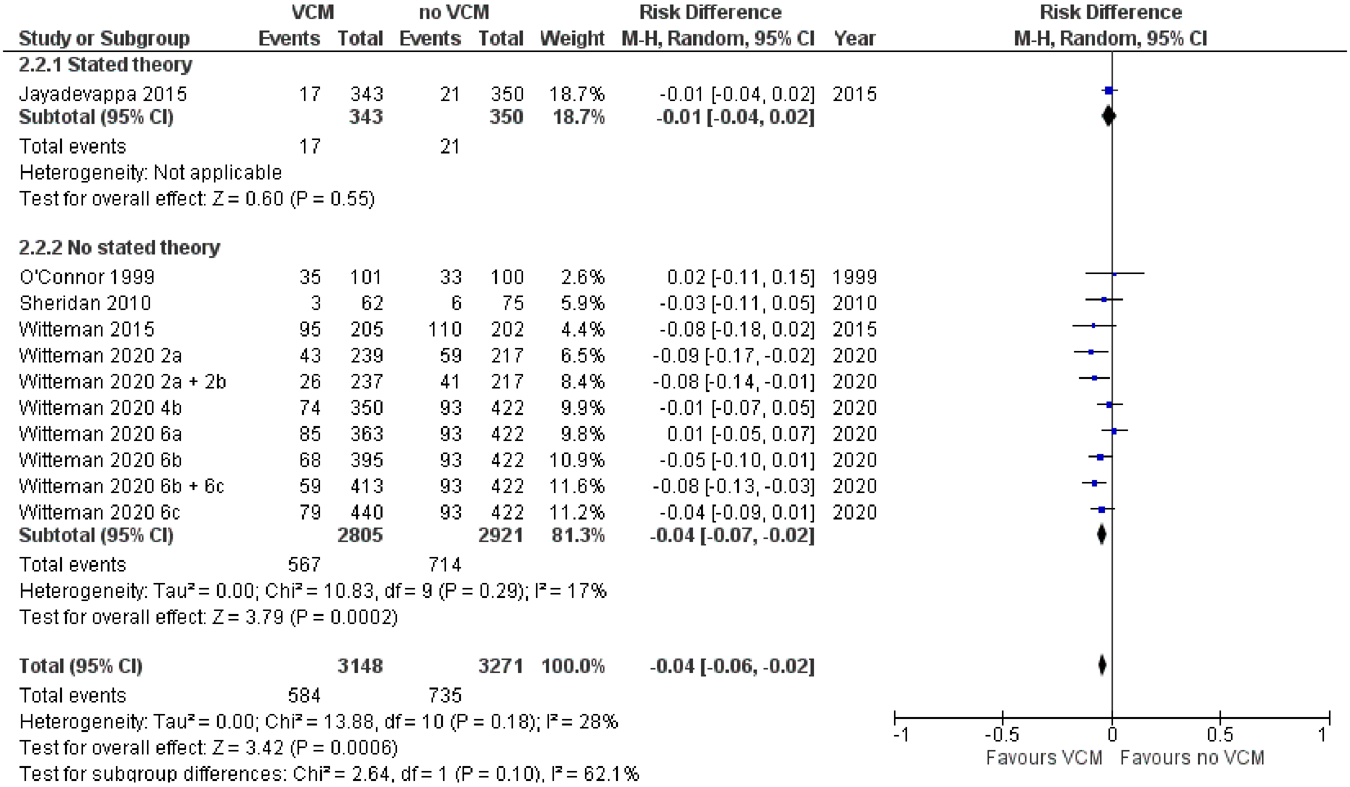

Figure S4. Risk of Values-Incongruent Decisions by Real/Hypothetical Decision

This analysis compares the risk of values-incongruent decisions for values clarification methods tested in the context of real decisions versus those tested in the context of hypothetical decisions.

	VCM		no VCM			Risk Difference	
**Study or Subgroup**	**Events**	**Total**	**Events**	**Total**	**Weight**	**M-H, Random, 95% CI**	**Year**
**2.2.1 Real decision**							
O'Connor 1999	35	101	33	100	2.6%	0.02 [-0.11, 0.15]	1999
Jayadevappa 2015	17	343	21	350	18.7%	-0.01 [-0.04, 0.02]	2015
Witteman 2015	95	205	110	202	4.4%	-0.08 [-0.18, 0.02]	2015
**Subtotal (95% CI)**		**649**		**652**	**25.7%**	**-0.02 [-0.07, 0.03]**	
Total events	147		164				
Heterogeneity: Tau² = 0.00; Chi² = 2.67, df = 2 (P = 0.26); I² = 25%							
Test for overall effect: Z = 0.86 (P = 0.39)							
**2.2.2 Hypothetical decision**							
Sheridan 2010	3	62	6	75	5.9%	-0.03 [-0.11, 0.05]	2010
Witteman 2020 4b	74	350	93	422	9.9%	-0.01 [-0.07, 0.05]	2020
Witteman 2020 6a	85	363	93	422	9.8%	0.01 [-0.05, 0.07]	2020
Witteman 2020 6b	68	395	93	422	10.9%	-0.05 [-0.10, 0.01]	2020
Witteman 2020 6b + 6c	59	413	93	422	11.6%	-0.08 [-0.13, -0.03]	2020
Witteman 2020 6c	79	440	93	422	11.2%	-0.04 [-0.09, 0.01]	2020
Witteman 2020 2a	43	239	59	217	6.5%	-0.09 [-0.17, -0.02]	2020
Witteman 2020 2a + 2b	26	237	41	217	8.4%	-0.08 [-0.14, -0.01]	2020
**Subtotal (95% CI)**		**2499**		**2619**	**74.3%**	**-0.04 [-0.07, -0.02]**	
Total events	437		571				
Heterogeneity: Tau² = 0.00; Chi² = 9.45, df = 7 (P = 0.22); I² = 26%							
Test for overall effect: Z = 3.47 (P = 0.0005)							
**Total (95% CI)**		**3148**		**3271**	**100.0%**	**-0.04 [-0.06, -0.02]**	
Total events	584		735				
Heterogeneity: Tau² = 0.00; Chi² = 13.88, df = 10 (P = 0.18); I² = 28%							
Test for overall effect: Z = 3.42 (P = 0.0006)							
Test for subgroup differences: Chi² = 0.68, df = 1 (P = 0.41), I² = 0%							

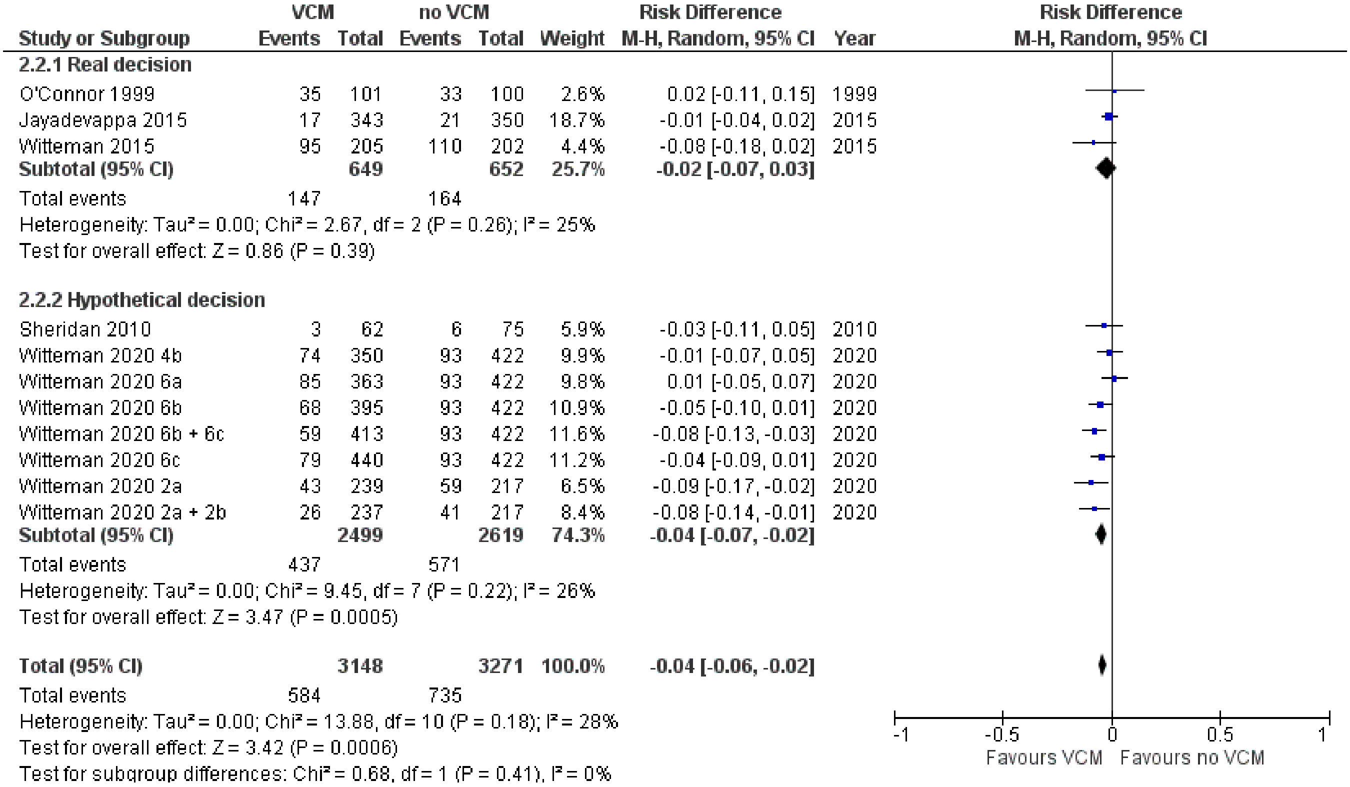

Figure S5. Decisional Conflict by Tradeoffs

This analysis compares decisional conflict for values clarification methods that explicitly require the user to engage with tradeoffs in some way versus those that do not require such engagement.

	VCM			no VCM				Std. Mean Difference	
**Study or Subgroup**	**Mean**	**SD**	**Total**	**Mean**	**SD**	**Total**	**Weight**	**IV, Random, 95% CI**	**Year**
**1.1.1 Explicit tradeoffs**									
Montgomery 2003	27.6	12.1	100	38.9	18.3	112	5.7%	-0.72 [-1.00, -0.44]	2003
Montgomery 2007	23.6	15.1	198	27.8	14.6	201	8.0%	-0.28 [-0.48, -0.09]	2007
Frosh 2008	27.95	13.45	153	30	10.77	151	7.1%	-0.17 [-0.39, 0.06]	2008
Kuppermann 2014	12.9	14.1	357	13.8	15.6	353	9.7%	-0.06 [-0.21, 0.09]	2014
Garvelink 2014	41.6	9.5	66	44.1	12.3	66	4.4%	-0.23 [-0.57, 0.12]	2014
Shirk 2017	2.5	0.9	59	2.2	0.9	63	4.2%	0.33 [-0.03, 0.69]	2017
Hopkin 2019	14.59	15.04	122	23.13	20.34	136	6.5%	-0.47 [-0.72, -0.22]	2019
Witteman 2020 6b + 6c	36	27	413	42	27	422	10.1%	-0.22 [-0.36, -0.09]	2020
Witteman 2020 6b	39	27	395	42	27	422	10.0%	-0.11 [-0.25, 0.03]	2020
Witteman 2020 4b	35	28	350	42	27	422	9.9%	-0.25 [-0.40, -0.11]	2020
**Subtotal (95% CI)**			**2213**			**2348**	**75.7%**	**-0.22 [-0.34, -0.10]**	
Heterogeneity: Tau² = 0.02; Chi² = 32.89, df = 9 (P = 0.0001); I² = 73%									
Test for overall effect: Z = 3.67 (P = 0.0002)									
**1.1.2 Not at all**									
Epstein 2018	9.09	2.35	61	9.48	2.23	61	4.2%	-0.17 [-0.52, 0.19]	2018
Witteman 2020 6c	36	26	440	42	27	422	10.2%	-0.23 [-0.36, -0.09]	2020
Witteman 2020 6a	35	27	363	42	27	422	9.9%	-0.26 [-0.40, -0.12]	2020
**Subtotal (95% CI)**			**864**			**905**	**24.3%**	**-0.24 [-0.33, -0.14]**	
Heterogeneity: Tau² = 0.00; Chi² = 0.26, df = 2 (P = 0.88); I² = 0%									
Test for overall effect: Z = 4.95 (P < 0.00001)									
**Total (95% CI)**			**3077**			**3253**	**100.0%**	**-0.22 [-0.31, -0.13]**	
Heterogeneity: Tau² = 0.02; Chi² = 33.47, df = 12 (P = 0.0008); I² = 64%									
Test for overall effect: Z = 4.94 (P < 0.00001)									
Test for subgroup differences: Chi² = 0.04, df = 1 (P = 0.85), I² = 0%									

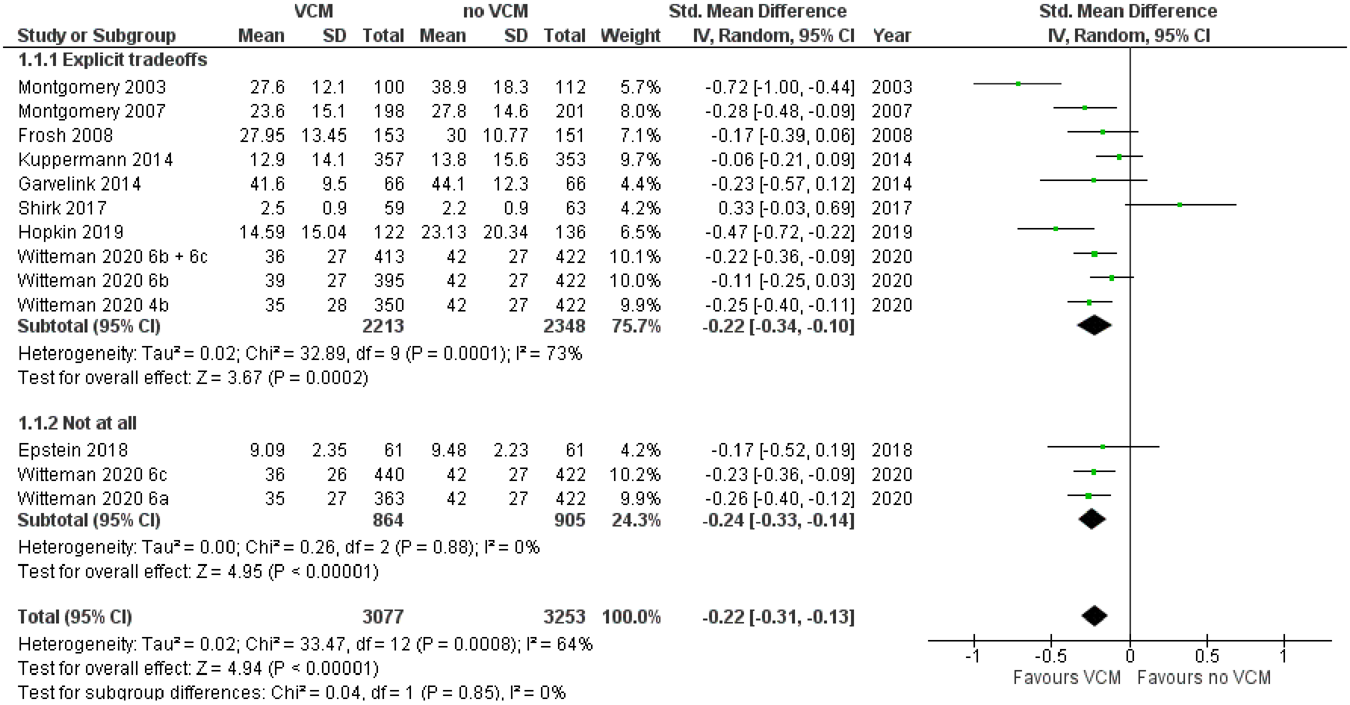

Figure S6. Decisional Conflict by Implications/Presentation of Results

This analysis compares decisional conflict for values clarification methods that explicitly show the user with the implications of what they value (i.e., present the user with the ‘results’ of their values clarification process) versus those that do not show the user any such thing.

	VCM			no VCM				Std. Mean Difference		Std. Mean Difference
**Study or Subgroup**	**Mean**	**SD**	**Total**	**Mean**	**SD**	**Total**	**Weight**	**IV, Random, 95% CI**	**Year**	**IV, Random, 95% CI**
**1.1.1 Presentation of results**										
Montgomery 2003	27.6	12.1	100	38.9	18.3	112	5.7%	-0.72 [-1.00, -0.44]	2003	
Montgomery 2007	23.6	15.1	198	27.8	14.6	201	8.0%	-0.28 [-0.48, -0.09]	2007	
Kuppermann 2014	12.9	14.1	357	13.8	15.6	353	9.7%	-0.06 [-0.21, 0.09]	2014	
Garvelink 2014	41.6	9.5	66	44.1	12.3	66	4.4%	-0.23 [-0.57, 0.12]	2014	
Shirk 2017	2.5	0.9	59	2.2	0.9	63	4.2%	0.33 [-0.03, 0.69]	2017	
Hopkin 2019	14.59	15.04	122	23.13	20.34	136	6.5%	-0.47 [-0.72, -0.22]	2019	
Witteman 2020 6c	36	26	440	42	27	422	10.2%	-0.23 [-0.36, -0.09]	2020	
Witteman 2020 6b + 6c	36	27	413	42	27	422	10.1%	-0.22 [-0.36, -0.09]	2020	
**Subtotal (95% CI)**			**1755**			**1775**	**58.8%**	**-0.24 [-0.39, -0.09]**		
Heterogeneity: Tau² = 0.03; Chi² = 30.29, df = 7 (P < 0.0001); I² = 77%										
Test for overall effect: Z = 3.19 (P = 0.001)										
**1.1.2 No presentation of results**										
Frosh 2008	27.95	13.45	153	30	10.77	151	7.1%	-0.17 [-0.39, 0.06]	2008	
Epstein 2018	9.09	2.35	61	9.48	2.23	61	4.2%	-0.17 [-0.52, 0.19]	2018	
Witteman 2020 6b	39	27	395	42	27	422	10.0%	-0.11 [-0.25, 0.03]	2020	
Witteman 2020 4b	35	28	350	42	27	422	9.9%	-0.25 [-0.40, -0.11]	2020	
Witteman 2020 6a	35	27	363	42	27	422	9.9%	-0.26 [-0.40, -0.12]	2020	
**Subtotal (95% CI)**			**1322**			**1478**	**41.2%**	**-0.20 [-0.27, -0.13]**		
Heterogeneity: Tau² = 0.00; Chi² = 2.96, df = 4 (P = 0.56); I² = 0%										
Test for overall effect: Z = 5.28 (P < 0.00001)										
**Total (95% CI)**			**3077**			**3253**	**100.0%**	**-0.22 [-0.31, -0.13]**		
Heterogeneity: Tau² = 0.02; Chi² = 33.47, df = 12 (P = 0.0008); I² = 64%										
Test for overall effect: Z = 4.94 (P < 0.00001)										
Test for subgroup differences: Chi² = 0.24, df = 1 (P = 0.62), I² = 0%										

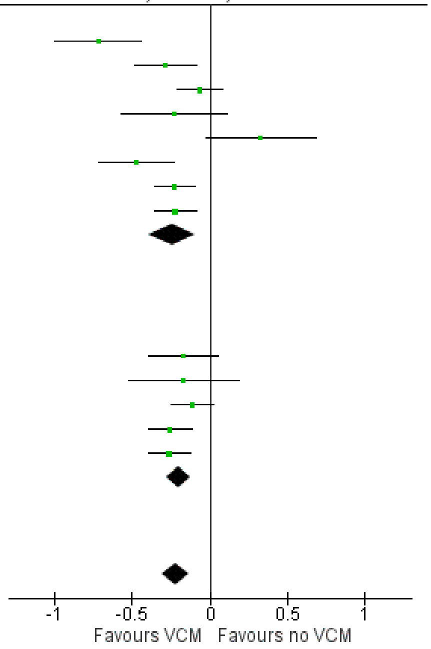

Figure S7. Decisional Conflict by Stated Use of a Theory/Framework

This analysis compares decisional conflict for values clarification methods that are underpinned by a formal theory or conceptual framework versus those that are not.

	VCM			no VCM				Std. Mean Difference	
**Study or Subgroup**	**Mean**	**SD**	**Total**	**Mean**	**SD**	**Total**	**Weight**	**IV, Random, 95% CI**	**Year**
**1.1.1 Stated theory**									
Montgomery 2003	27.6	12.1	100	38.9	18.3	112	5.7%	-0.72 [-1.00, -0.44]	2003
Montgomery 2007	23.6	15.1	198	27.8	14.6	201	8.0%	-0.28 [-0.48, -0.09]	2007
Shirk 2017	2.5	0.9	59	2.2	0.9	63	4.2%	0.33 [-0.03, 0.69]	2017
Epstein 2018	9.09	2.35	61	9.48	2.23	61	4.2%	-0.17 [-0.52, 0.19]	2018
Hopkin 2019	14.59	15.04	122	23.13	20.34	136	6.5%	-0.47 [-0.72, -0.22]	2019
**Subtotal (95% CI)**			**540**			**573**	**28.6%**	**-0.28 [-0.57, 0.02]**	
Heterogeneity: Tau² = 0.09; Chi² = 22.86, df = 4 (P = 0.0001); I² = 83%									
Test for overall effect: Z = 1.85 (P = 0.06)									
**1.1.2 No stated theory or framework**									
Frosh 2008	27.95	13.45	153	30	10.77	151	7.1%	-0.17 [-0.39, 0.06]	2008
Garvelink 2014	41.6	9.5	66	44.1	12.3	66	4.4%	-0.23 [-0.57, 0.12]	2014
Kuppermann 2014	12.9	14.1	357	13.8	15.6	353	9.7%	-0.06 [-0.21, 0.09]	2014
Witteman 2020 6b	39	27	395	42	27	422	10.0%	-0.11 [-0.25, 0.03]	2020
Witteman 2020 4b	35	28	350	42	27	422	9.9%	-0.25 [-0.40, -0.11]	2020
Witteman 2020 6c	36	26	440	42	27	422	10.2%	-0.23 [-0.36, -0.09]	2020
Witteman 2020 6a	35	27	363	42	27	422	9.9%	-0.26 [-0.40, -0.12]	2020
Witteman 2020 6b + 6c	36	27	413	42	27	422	10.1%	-0.22 [-0.36, -0.09]	2020
**Subtotal (95% CI)**			**2537**			**2680**	**71.4%**	**-0.19 [-0.24, -0.14]**	
Heterogeneity: Tau² = 0.00; Chi² = 6.54, df = 7 (P = 0.48); I² = 0%									
Test for overall effect: Z = 6.85 (P < 0.00001)									
**Total (95% CI)**			**3077**			**3253**	**100.0%**	**-0.22 [-0.31, -0.13]**	
Heterogeneity: Tau² = 0.02; Chi² = 33.47, df = 12 (P = 0.0008); I² = 64%									
Test for overall effect: Z = 4.94 (P < 0.00001)									
Test for subgroup differences: Chi² = 0.33, df = 1 (P = 0.56), I² = 0%									

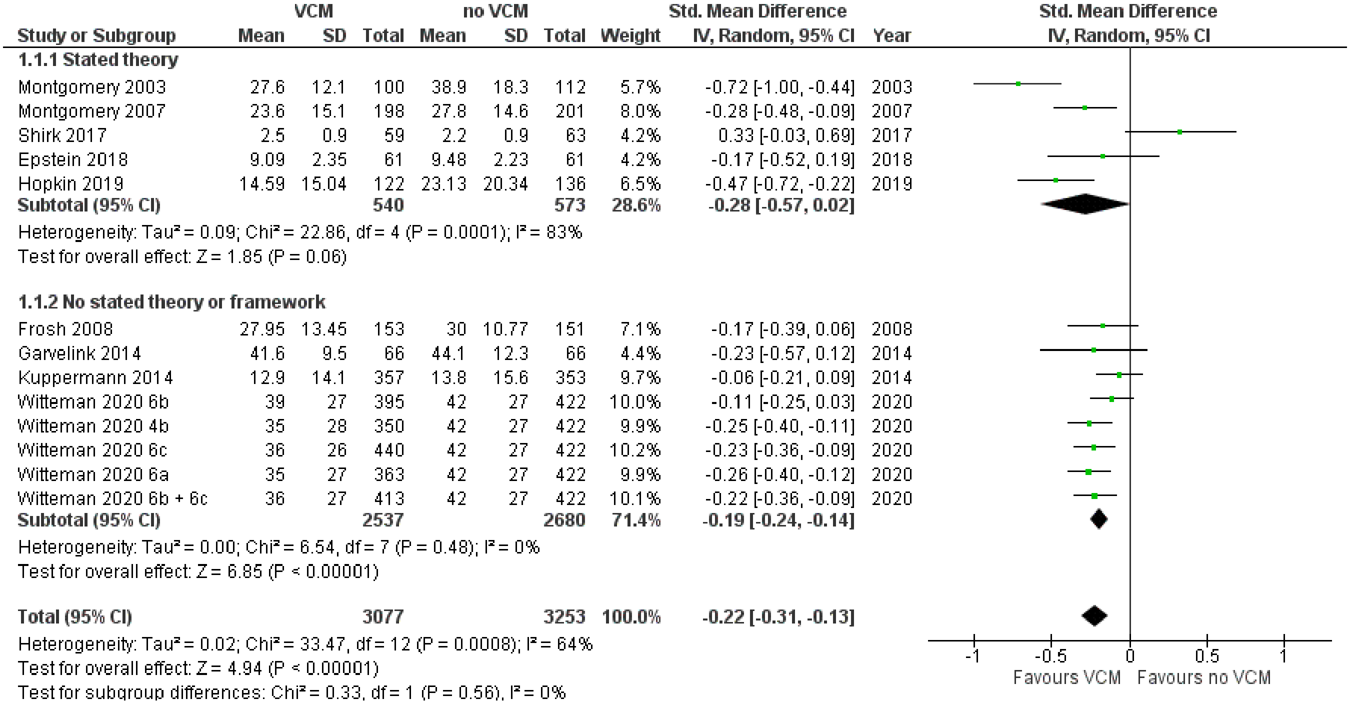

Figure S8. Decisional Conflict by Real/Hypothetical

This analysis compares decisional conflict for values clarification methods tested in the context of real decisions versus those tested in the context of hypothetical decisions.

	VCM			no VCM				Std. Mean Difference	
**Study or Subgroup**	**Mean**	**SD**	**Total**	**Mean**	**SD**	**Total**	**Weight**	**IV, Random, 95% CI**	**Year**
**1.1.1 Real decision**									
Montgomery 2003	27.6	12.1	100	38.9	18.3	112	5.7%	-0.72 [-1.00, -0.44]	2003
Montgomery 2007	23.6	15.1	198	27.8	14.6	201	8.0%	-0.28 [-0.48, -0.09]	2007
Frosh 2008	27.95	13.45	153	30	10.77	151	7.1%	-0.17 [-0.39, 0.06]	2008
Kuppermann 2014	12.9	14.1	357	13.8	15.6	353	9.7%	-0.06 [-0.21, 0.09]	2014
Shirk 2017	2.5	0.9	59	2.2	0.9	63	4.2%	0.33 [-0.03, 0.69]	2017
Epstein 2018	9.09	2.35	61	9.48	2.23	61	4.2%	-0.17 [-0.52, 0.19]	2018
**Subtotal (95% CI)**			**928**			**941**	**39.0%**	**-0.19 [-0.41, 0.03]**	
Heterogeneity: Tau² = 0.06; Chi² = 25.78, df = 5 (P < 0.0001); I² = 81%									
Test for overall effect: Z = 1.66 (P = 0.10)									
**1.1.2 Hypothetical decision**									
Garvelink 2014	41.6	9.5	66	44.1	12.3	66	4.4%	-0.23 [-0.57, 0.12]	2014
Hopkin 2019	14.59	15.04	122	23.13	20.34	136	6.5%	-0.47 [-0.72, -0.22]	2019
Witteman 2020 6b	39	27	395	42	27	422	10.0%	-0.11 [-0.25, 0.03]	2020
Witteman 2020 4b	35	28	350	42	27	422	9.9%	-0.25 [-0.40, -0.11]	2020
Witteman 2020 6b + 6c	36	27	413	42	27	422	10.1%	-0.22 [-0.36, -0.09]	2020
Witteman 2020 6c	36	26	440	42	27	422	10.2%	-0.23 [-0.36, -0.09]	2020
Witteman 2020 6a	35	27	363	42	27	422	9.9%	-0.26 [-0.40, -0.12]	2020
**Subtotal (95% CI)**			**2149**			**2312**	**61.0%**	**-0.23 [-0.29, -0.17]**	
Heterogeneity: Tau² = 0.00; Chi² = 6.85, df = 6 (P = 0.34); I² = 12%									
Test for overall effect: Z = 7.07 (P < 0.00001)									
**Total (95% CI)**			**3077**			**3253**	**100.0%**	**-0.22 [-0.31, -0.13]**	
Heterogeneity: Tau² = 0.02; Chi² = 33.47, df = 12 (P = 0.0008); I² = 64%									
Test for overall effect: Z = 4.94 (P < 0.00001)									
Test for subgroup differences: Chi² = 0.13, df = 1 (P = 0.72), I² = 0%									

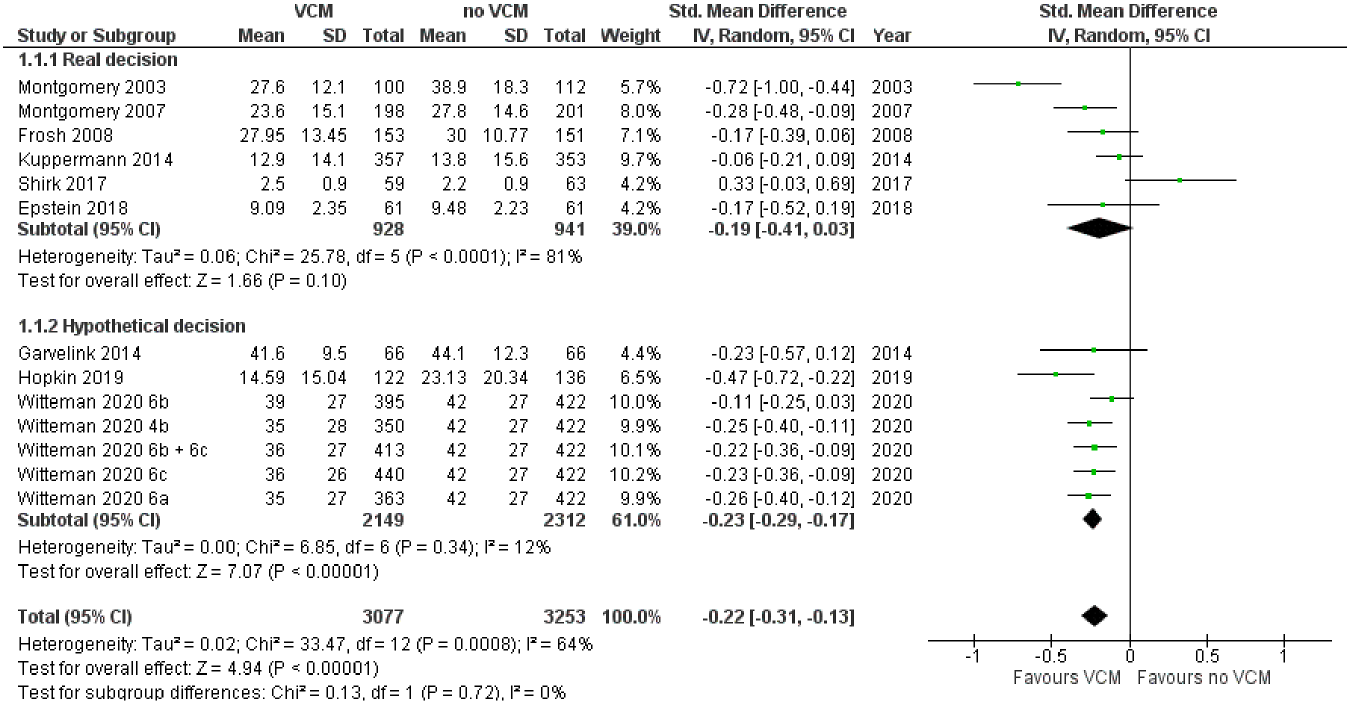

Figure S9. Decisional Conflict by Risk of Bias

This analysis compares decisional conflict for values clarification methods tested in the context of low risk of bias or some concerns versus those tested in the context of high risk of bias. Note that high risk of bias may be necessary in some cases due to the nature of values clarification methods. For example, it may be difficult to conceal the purpose of an intervention from the person using it.

	VCM			no VCM				Std. Mean Difference	
**Study or Subgroup**	**Mean**	**SD**	**Total**	**Mean**	**SD**	**Total**	**Weight**	**IV, Random, 95% CI**	**Year**
**1.1.1 Low risk of bias or some concerns**									
Montgomery 2007	23.6	15.1	198	27.8	14.6	201	7.7%	-0.28 [-0.48, -0.09]	2007
Frosh 2008	27.95	13.45	153	30	10.77	151	6.9%	-0.17 [-0.39, 0.06]	2008
Garvelink 2014 2	41.6	9.5	66	44.1	12.3	65	4.4%	-0.23 [-0.57, 0.12]	2014
Kuppermann 2014	12.9	14.1	357	13.8	15.6	353	9.1%	-0.06 [-0.21, 0.09]	2014
Shirk 2017	2.5	0.9	59	2.2	0.9	63	4.2%	0.33 [-0.03, 0.69]	2017
Epstein 2018	9.09	2.35	61	9.48	2.23	61	4.3%	-0.17 [-0.52, 0.19]	2018
Hopkin 2019	14.59	15.04	122	23.13	20.34	136	6.4%	-0.47 [-0.72, -0.22]	2019
Witteman 2020 6b + 6c	36	27	413	42	27	422	9.4%	-0.22 [-0.36, -0.09]	2020
Witteman 2020 6c	36	26	440	42	27	422	9.5%	-0.23 [-0.36, -0.09]	2020
Witteman 2020 4b	35	28	350	42	27	422	9.2%	-0.25 [-0.40, -0.11]	2020
Witteman 2020 6a	35	27	363	42	27	422	9.3%	-0.26 [-0.40, -0.12]	2020
Witteman 2020 6b	39	27	395	42	27	422	9.4%	-0.11 [-0.25, 0.03]	2020
**Subtotal (95% CI)**			**2977**			**3140**	**89.7%**	**-0.20 [-0.27, -0.12]**	
Heterogeneity: Tau² = 0.01; Chi² = 20.46, df = 11 (P = 0.04); I² = 46%									
Test for overall effect: Z = 5.26 (P < 0.00001)									
**1.1.2 High risk of bias**									
Montgomery 2003	27.6	12.1	100	38.9	18.3	112	5.7%	-0.72 [-1.00, -0.44]	2003
Garvelink 2014 1	43.6	14.2	70	40.9	11.6	70	4.6%	0.21 [-0.13, 0.54]	2014
**Subtotal (95% CI)**			**170**			**182**	**10.3%**	**-0.26 [-1.17, 0.65]**	
Heterogeneity: Tau² = 0.40; Chi² = 17.50, df = 1 (P < 0.0001); I² = 94%									
Test for overall effect: Z = 0.56 (P = 0.57)									
**Total (95% CI)**			**3147**			**3322**	**100.0%**	**-0.20 [-0.29, -0.11]**	
Heterogeneity: Tau² = 0.02; Chi² = 39.50, df = 13 (P = 0.0002); I² = 67%									
Test for overall effect: Z = 4.35 (P < 0.0001)									
Test for subgroup differences: Chi² = 0.02, df = 1 (P = 0.89), I² = 0%									

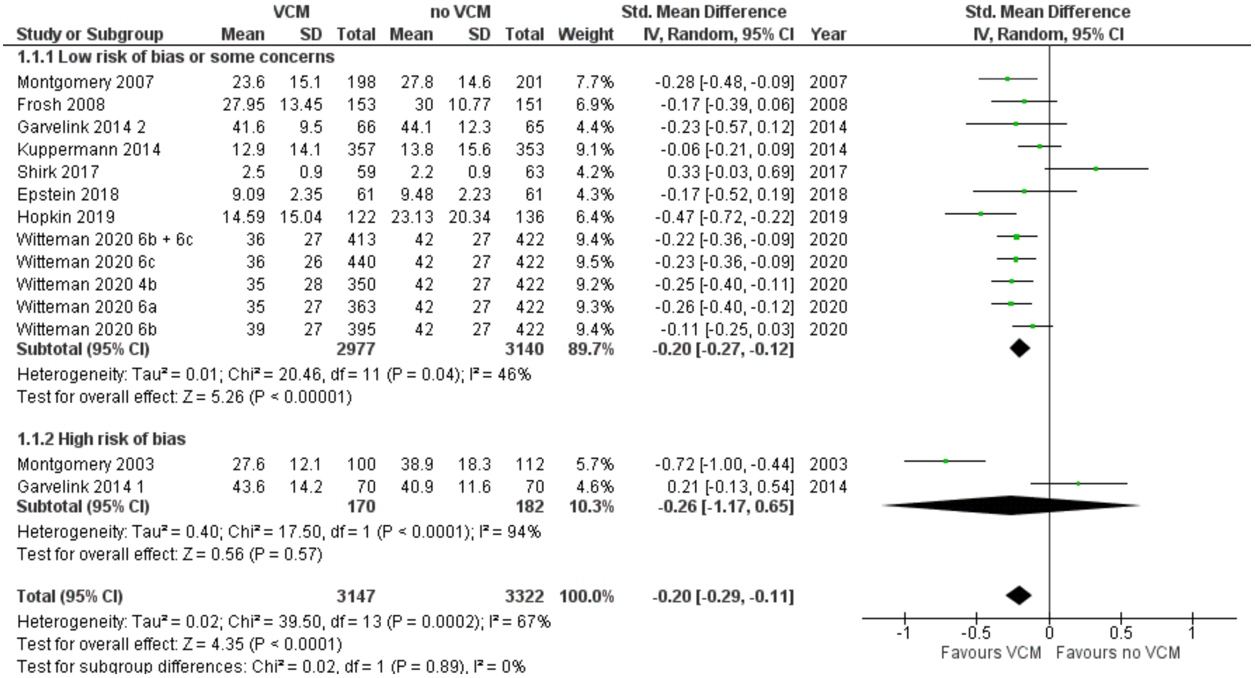

Head-to-Head Evaluations of Values Clarification Methods

Feldman-Stewart et al. (2006) found no difference across all three groups (information only; values clarification method without a summary bar, i.e. rating scales; values clarification with a summary bar, i.e. multicriteria decision analysis) in terms of the attributes participants identified as important to their decisions nor in how difficult it was to make the decision. When trial participants were unblinded at the end of the study and shown all three options, all of them ranked the bars with the summary option (multicriteria decision analysis) as the most helpful.

Pignone et al. (2012) found that a discrete choice experiment produced somewhat different patterns of attribute importance compared to ranking and rating. Agreement between the most important attribute derived from the values clarification method and the most important attribute as reported by participants in the questionnaire was slightly higher in the ranking and rating arm than the discrete choice experiment arm. The authors found no difference between study arms in terms of values clarity, intent to be screened and unlabelled screening test preference.

Pignone et al. (2013) found that different values clarification methods produced differences in attribute importance and screening test preference. Participants who received the rating and ranking test were more likely to report the chance of dying from prostate cancer as the most important attribute compared to the balance sheet and discrete choice experiment groups. Those who received the balance sheet were more likely to prefer the unlabelled PSA-like test option compared to the two other groups. Participants who received the discrete choice experiment were somewhat less likely to select reduction of mortality as the most important attribute, and were least likely to select the PSA-like option on the unlabelled preference question. There was no difference across groups in intent to be screened (labelled PSA test option) nor on values clarity.

Brenner et al. (2014) found that different values clarification methods produced different results in terms of individuals' most important screening test attributes. Specifically, respondents who received the rating and ranking exercise, compared to a discrete choice experiment or a balance sheet (i.e., implicit values clarification method), were the most likely to choose risk reduction as the most important attribute. They found no differences in terms of test preferences, values clarity, nor intention to be screened.

Witteman et al. (2020) found that overall, methods using mathematical models (e.g., decision analysis, allocation of points) were more promising than other methods (e.g., pros and cons, rating scales) for encouraging values-congruent decisions. All methods encouraged lower decisional conflict when this was assessed.

Risk of Bias

Article	Random Sequence Generation (Selection Bias)	Allocation Concealme nt (Selection Bias)	Blinding of Participants and Personnel (Performanc e Bias)	Blinding of Outcome Assessment (Detection Bias)	Incomplete Outcome Data (Attrition Data)	Selective Reporting (Reporting Bias)	Other Bias
Abhyankar 2010	low	unclear	high	unclear	low	low	low
Au 2012	low	low	unclear	unclear	unclear	unclear	unclear
Bekker 2004	low	low	unclear	unclear	unclear	low	low
Brenner 2014	low	low	low	unclear	unclear	low	low
Clancy 1988	low	unclear	unclear	low	unclear	unclear	high
deAchaval 2012	low	low	low	low	low	low	low
Epstein 2018	low	unclear	unclear	high	unclear	low	unclear
Feldman-Stewart 2006	unclear	unclear	unclear	unclear	unclear	unclear	low
Feldman-Stewart 2012	low	low	unclear	low	low	unclear	low
Fraenken 2007	low	low	high	unclear	low	low	low
Frosch 2008	low	low	unclear	low	low	unclear	low
Garvelink 2014 (1)	low	low	low	low	unclear	low	high
Garvelink 2014 (2)	low	low	low	low	low	low	unclear
Hess 2015	low	low	unclear	unclear	unclear	low	unclear
Hopkin 2019	low	low	low	low	unclear	low	unclear
Hutyra 2019	low	unclear	low	low	low	low	unclear
Jayadevappa 2019	low	low	unclear	unclear	low	unclear	unclear
Kennedy 2002	low	low	high	unclear	low	unclear	unclear
Kuppermann 2014	low	low	low	low	low	low	unclear
Lehrman 1997	unclear	unclear	high	unclear	low	low	low
Matheis-Kraft 1997	unclear	unclear	unclear	unclear	low	unclear	unclear
Montgomery 2007	low	low	low	low	low	low	unclear
Montgomery 2003	low	low	high	low	low	unclear	low

Myers 2003	unclear	unclear	unclear	unclear	unclear	low	unclear
Myers 2005	unclear	unclear	unclear	unclear	low	low	unclear
O'Connor 1999	low	low	unclear	low	low	unclear	low
Paquin 2018	low	low	unclear	unclear	low	low	unclear
Peinado 2020	unclear	low	unclear	unclear	unclear	low	low
Pignone 2012	unclear	unclear	low	unclear	unclear	low	low
Pignone 2013	low	low	low	unclear	unclear	low	low
Sheridan 2010	low	low	low	low	low	low	low
Shirk 2017	unclear	unclear	unclear	unclear	low	low	unclear
Witteman 2015	low	low	unclear	unclear	unclear	low	unclear
Witteman 2020	unclear	low	unclear	unclear	low	low	low

Sensitivity Analyses of I^2 Estimates

Because Witteman 2020 included two comparisons from one study population (2a; 2a + 2b) and five comparisons from another study population (4b; 6a; 6b; 6c; 6b + 6c), for each outcome, we conducted 10 additional meta-analyses (2 x 5 possible combinations) using a random effects model and 1 additional meta-analysis using a fixed effect model on all comparisons contributed by Witteman 2020. This allowed us to assess I^2 sensitivity to the inclusion of multiple comparisons drawing from the same study populations. Overall, we found that the I^2 estimates were robust to the inclusion of multiple comparisons.

Figure S10. Risk of Values-Incongruent Decisions with Witteman 2020 2a and 4b

Study or Subgroup	VCM Events	VCM Total	no VCM Events	no VCM Total	Weight	Risk Difference M-H, Random, 95% CI	Year
**2.2.1 Other articles and Witteman 2a, 4b**							
O'Connor 1999	35	101	33	100	2.6%	0.02 [-0.11, 0.15]	1999
Sheridan 2010	3	62	6	75	5.9%	-0.03 [-0.11, 0.05]	2010
Jayadevappa 2015	17	343	21	350	18.7%	-0.01 [-0.04, 0.02]	2015
Witteman 2015	95	205	110	202	4.4%	-0.08 [-0.18, 0.02]	2015
Witteman 2020 4b	74	350	93	422	9.9%	-0.01 [-0.07, 0.05]	2020
Witteman 2020 2a	43	239	59	217	6.5%	-0.09 [-0.17, -0.02]	2020
**Subtotal (95% CI)**		**1300**		**1366**	**48.1%**	**-0.03 [-0.06, 0.00]**	
Total events	267		322				
Heterogeneity: Tau² = 0.00; Chi² = 6.24, df = 5 (P = 0.28); I² = 20%							
Test for overall effect: Z = 1.82 (P = 0.07)							
**2.2.2 Remaining Witteman 2020**							
Witteman 2020 6a	85	363	93	422	9.8%	0.01 [-0.05, 0.07]	2020
Witteman 2020 6b	68	395	93	422	10.9%	-0.05 [-0.10, 0.01]	2020
Witteman 2020 6b + 6c	59	413	93	422	11.6%	-0.08 [-0.13, -0.03]	2020
Witteman 2020 6c	79	440	93	422	11.2%	-0.04 [-0.09, 0.01]	2020
Witteman 2020 2a + 2b	26	237	41	217	8.4%	-0.08 [-0.14, -0.01]	2020
**Subtotal (95% CI)**		**1848**		**1905**	**51.9%**	**-0.05 [-0.08, -0.01]**	
Total events	317		413				
Heterogeneity: Tau² = 0.00; Chi² = 6.45, df = 4 (P = 0.17); I² = 38%							
Test for overall effect: Z = 2.84 (P = 0.005)							
**Total (95% CI)**		**3148**		**3271**	**100.0%**	**-0.04 [-0.06, -0.02]**	
Total events	584		735				
Heterogeneity: Tau² = 0.00; Chi² = 13.88, df = 10 (P = 0.18); I² = 28%							
Test for overall effect: Z = 3.42 (P = 0.0006)							
Test for subgroup differences: Chi² = 0.65, df = 1 (P = 0.42), I² = 0%							

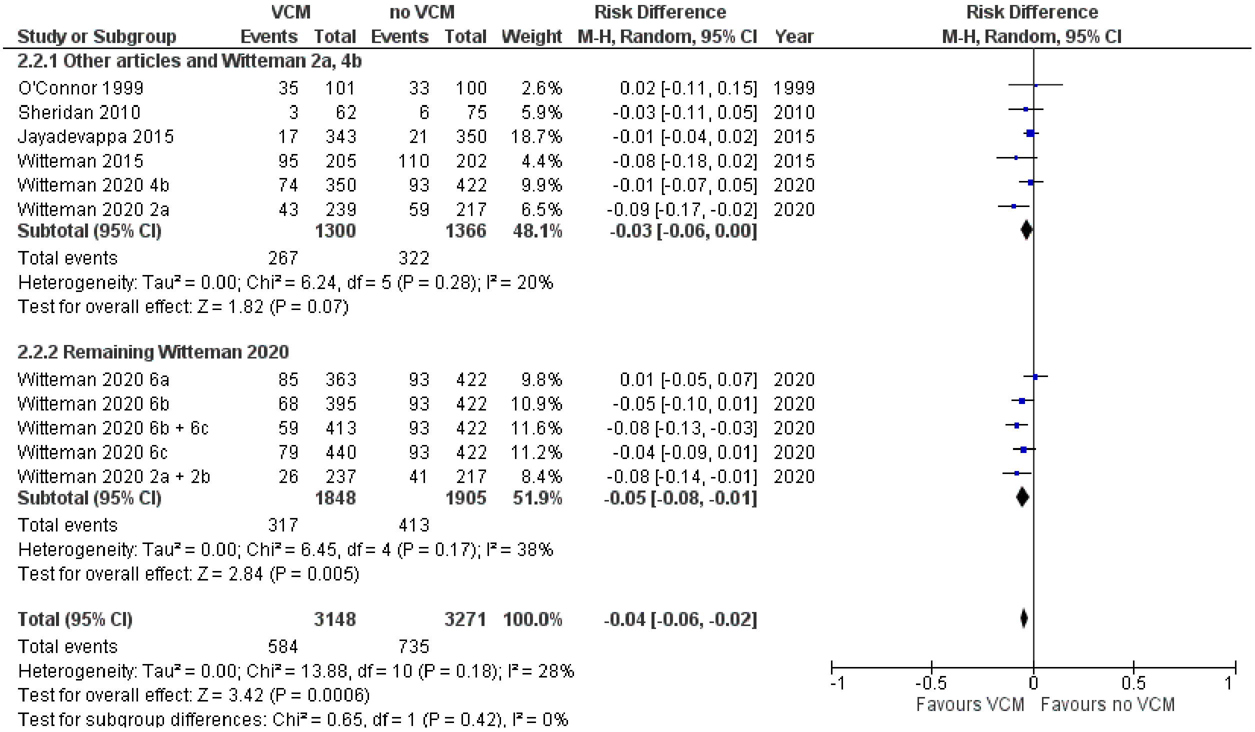

Figure S11. Risk of Values-Incongruent Decisions with Witteman 2020 2a and 6a

Study or Subgroup	VCM Events	VCM Total	no VCM Events	no VCM Total	Weight	Risk Difference M-H, Random, 95% CI	Year
**2.2.1 Other articles and Witteman 2a, 6a**							
O'Connor 1999	35	101	33	100	2.6%	0.02 [-0.11, 0.15]	1999
Sheridan 2010	3	62	6	75	5.9%	-0.03 [-0.11, 0.05]	2010
Jayadevappa 2015	17	343	21	350	18.7%	-0.01 [-0.04, 0.02]	2015
Witteman 2015	95	205	110	202	4.4%	-0.08 [-0.18, 0.02]	2015
Witteman 2020 6a	85	363	93	422	9.8%	0.01 [-0.05, 0.07]	2020
Witteman 2020 2a	43	239	59	217	6.5%	-0.09 [-0.17, -0.02]	2020
**Subtotal (95% CI)**		**1313**		**1366**	**48.0%**	**-0.03 [-0.06, 0.01]**	
Total events	278		322				
Heterogeneity: Tau² = 0.00; Chi² = 7.17, df = 5 (P = 0.21); I² = 30%							
Test for overall effect: Z = 1.48 (P = 0.14)							
**2.2.2 Remaining Witteman 2020**							
Witteman 2020 6c	79	440	93	422	11.2%	-0.04 [-0.09, 0.01]	2020
Witteman 2020 2a + 2b	26	237	41	217	8.4%	-0.08 [-0.14, -0.01]	2020
Witteman 2020 4b	74	350	93	422	9.9%	-0.01 [-0.07, 0.05]	2020
Witteman 2020 6b	68	395	93	422	10.9%	-0.05 [-0.10, 0.01]	2020
Witteman 2020 6b + 6c	59	413	93	422	11.6%	-0.08 [-0.13, -0.03]	2020
**Subtotal (95% CI)**		**1835**		**1905**	**52.0%**	**-0.05 [-0.08, -0.03]**	
Total events	306		413				
Heterogeneity: Tau² = 0.00; Chi² = 3.88, df = 4 (P = 0.42); I² = 0%							
Test for overall effect: Z = 3.96 (P < 0.0001)							
**Total (95% CI)**		**3148**		**3271**	**100.0%**	**-0.04 [-0.06, -0.02]**	
Total events	584		735				
Heterogeneity: Tau² = 0.00; Chi² = 13.88, df = 10 (P = 0.18); I² = 28%							
Test for overall effect: Z = 3.42 (P = 0.0006)							
Test for subgroup differences: Chi² = 1.41, df = 1 (P = 0.24), I² = 28.9%							

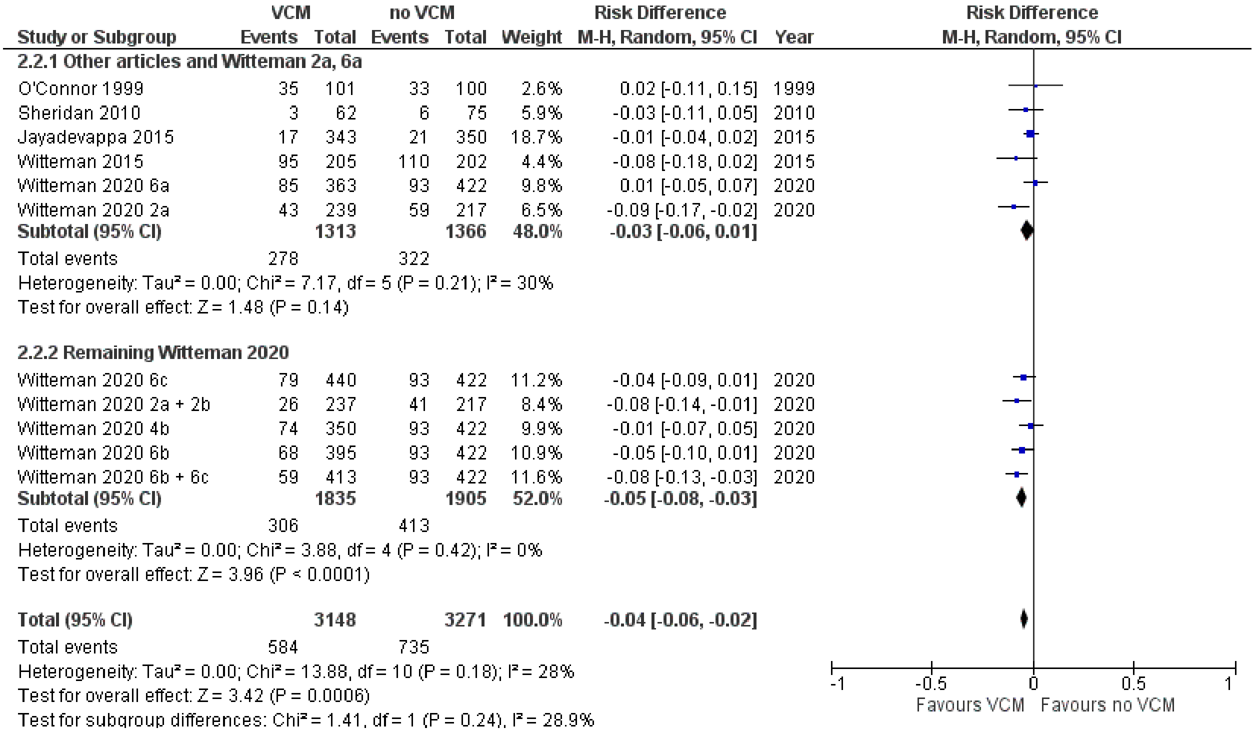

Figure S12. Risk of Values-Incongruent Decisions with Witteman 2020 2a and 6b

Study or Subgroup	VCM Events	VCM Total	no VCM Events	no VCM Total	Weight	Risk Difference M-H, Random, 95% CI	Year
**2.2.1 Other articles and Witteman 2a, 6b**							
O'Connor 1999	35	101	33	100	2.6%	0.02 [-0.11, 0.15]	1999
Sheridan 2010	3	62	6	75	5.9%	-0.03 [-0.11, 0.05]	2010
Jayadevappa 2015	17	343	21	350	18.7%	-0.01 [-0.04, 0.02]	2015
Witteman 2015	95	205	110	202	4.4%	-0.08 [-0.18, 0.02]	2015
Witteman 2020 2a	43	239	59	217	6.5%	-0.09 [-0.17, -0.02]	2020
Witteman 2020 6b	68	395	93	422	10.9%	-0.05 [-0.10, 0.01]	2020
**Subtotal (95% CI)**		**1345**		**1366**	**49.1%**	**-0.04 [-0.07, -0.01]**	
Total events	261		322				
Heterogeneity: Tau² = 0.00; Chi² = 6.99, df = 5 (P = 0.22); I² = 28%							
Test for overall effect: Z = 2.30 (P = 0.02)							
**2.2.2 Remaining Witteman 2020**							
Witteman 2020 6a	85	363	93	422	9.8%	0.01 [-0.05, 0.07]	2020
Witteman 2020 6c	79	440	93	422	11.2%	-0.04 [-0.09, 0.01]	2020
Witteman 2020 2a + 2b	26	237	41	217	8.4%	-0.08 [-0.14, -0.01]	2020
Witteman 2020 4b	74	350	93	422	9.9%	-0.01 [-0.07, 0.05]	2020
Witteman 2020 6b + 6c	59	413	93	422	11.6%	-0.08 [-0.13, -0.03]	2020
**Subtotal (95% CI)**		**1803**		**1905**	**50.9%**	**-0.04 [-0.07, -0.00]**	
Total events	323		413				
Heterogeneity: Tau² = 0.00; Chi² = 7.74, df = 4 (P = 0.10); I² = 48%							
Test for overall effect: Z = 2.12 (P = 0.03)							
**Total (95% CI)**		**3148**		**3271**	**100.0%**	**-0.04 [-0.06, -0.02]**	
Total events	584		735				
Heterogeneity: Tau² = 0.00; Chi² = 13.88, df = 10 (P = 0.18); I² = 28%							
Test for overall effect: Z = 3.42 (P = 0.0006)							
Test for subgroup differences: Chi² = 0.00, df = 1 (P = 0.98), I² = 0%							

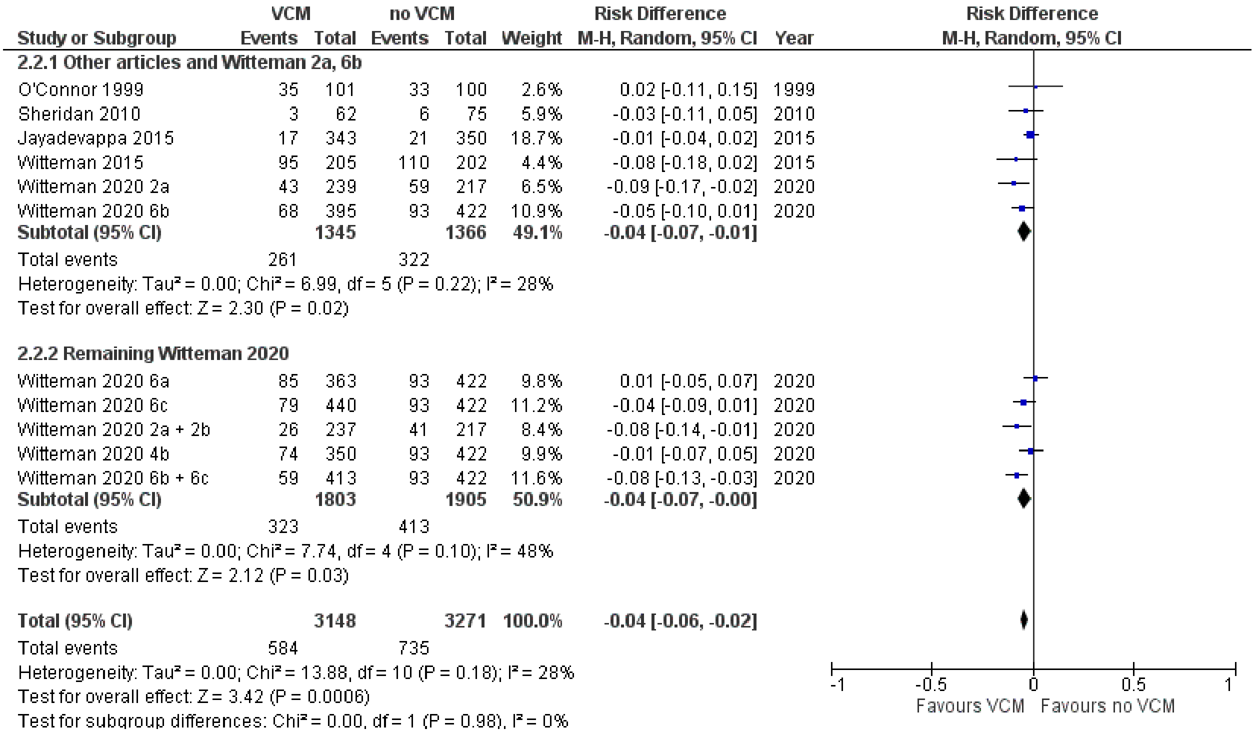

Figure S13. Risk of Values-Incongruent Decisions with Witteman 2020 2a and 6c

Study or Subgroup	VCM Events	VCM Total	no VCM Events	no VCM Total	Weight	Risk Difference M-H, Random, 95% CI	Year
**2.2.1 Other articles and Witteman 2a, 6c**							
O'Connor 1999	35	101	33	100	2.6%	0.02 [-0.11, 0.15]	1999
Sheridan 2010	3	62	6	75	5.9%	-0.03 [-0.11, 0.05]	2010
Jayadevappa 2015	17	343	21	350	18.7%	-0.01 [-0.04, 0.02]	2015
Witteman 2015	95	205	110	202	4.4%	-0.08 [-0.18, 0.02]	2015
Witteman 2020 2a	43	239	59	217	6.5%	-0.09 [-0.17, -0.02]	2020
Witteman 2020 6c	79	440	93	422	11.2%	-0.04 [-0.09, 0.01]	2020
**Subtotal (95% CI)**		**1390**		**1366**	**49.4%**	**-0.04 [-0.07, -0.00]**	
Total events	272		322				
Heterogeneity: Tau² = 0.00; Chi² = 6.59, df = 5 (P = 0.25); I² = 24%							
Test for overall effect: Z = 2.25 (P = 0.02)							
**2.2.2 Remaining Witteman 2020**							
Witteman 2020 6b + 6c	59	413	93	422	11.6%	-0.08 [-0.13, -0.03]	2020
Witteman 2020 6b	68	395	93	422	10.9%	-0.05 [-0.10, 0.01]	2020
Witteman 2020 6a	85	363	93	422	9.8%	0.01 [-0.05, 0.07]	2020
Witteman 2020 2a + 2b	26	237	41	217	8.4%	-0.08 [-0.14, -0.01]	2020
Witteman 2020 4b	74	350	93	422	9.9%	-0.01 [-0.07, 0.05]	2020
**Subtotal (95% CI)**		**1758**		**1905**	**50.6%**	**-0.04 [-0.08, -0.00]**	
Total events	312		413				
Heterogeneity: Tau² = 0.00; Chi² = 7.83, df = 4 (P = 0.10); I² = 49%							
Test for overall effect: Z = 2.19 (P = 0.03)							
**Total (95% CI)**		**3148**		**3271**	**100.0%**	**-0.04 [-0.06, -0.02]**	
Total events	584		735				
Heterogeneity: Tau² = 0.00; Chi² = 13.88, df = 10 (P = 0.18); I² = 28%							
Test for overall effect: Z = 3.42 (P = 0.0006)							
Test for subgroup differences: Chi² = 0.04, df = 1 (P = 0.85), I² = 0%							

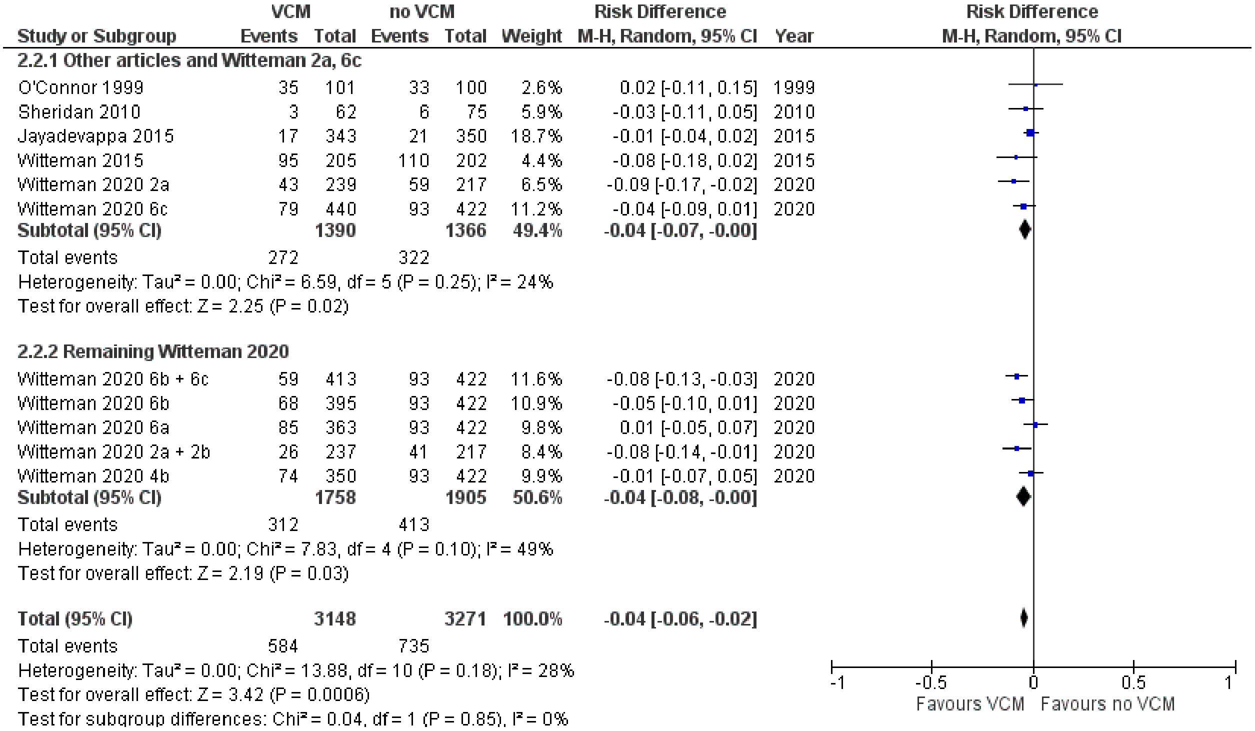

Figure S14. Risk of Values-Incongruent Decisions with Witteman 2020 2a and 6b + 6c

Study or Subgroup	VCM Events	VCM Total	no VCM Events	no VCM Total	Weight	Risk Difference M-H, Random, 95% CI	Year
**2.2.1 Other articles and Witteman 2a, 6b+6c**							
O'Connor 1999	35	101	33	100	2.6%	0.02 [-0.11, 0.15]	1999
Sheridan 2010	3	62	6	75	5.9%	-0.03 [-0.11, 0.05]	2010
Jayadevappa 2015	17	343	21	350	18.7%	-0.01 [-0.04, 0.02]	2015
Witteman 2015	95	205	110	202	4.4%	-0.08 [-0.18, 0.02]	2015
Witteman 2020 6b + 6c	59	413	93	422	11.6%	-0.08 [-0.13, -0.03]	2020
Witteman 2020 2a	43	239	59	217	6.5%	-0.09 [-0.17, -0.02]	2020
**Subtotal (95% CI)**		**1363**		**1366**	**49.8%**	**-0.05 [-0.09, -0.01]**	
Total events	252		322				
Heterogeneity: Tau² = 0.00; Chi² = 9.84, df = 5 (P = 0.08); I² = 49%							
Test for overall effect: Z = 2.36 (P = 0.02)							
**2.2.2 Remaining Witteman 2020**							
Witteman 2020 6c	79	440	93	422	11.2%	-0.04 [-0.09, 0.01]	2020
Witteman 2020 6b	68	395	93	422	10.9%	-0.05 [-0.10, 0.01]	2020
Witteman 2020 6a	85	363	93	422	9.8%	0.01 [-0.05, 0.07]	2020
Witteman 2020 2a + 2b	26	237	41	217	8.4%	-0.08 [-0.14, -0.01]	2020
Witteman 2020 4b	74	350	93	422	9.9%	-0.01 [-0.07, 0.05]	2020
**Subtotal (95% CI)**		**1785**		**1905**	**50.2%**	**-0.03 [-0.06, -0.00]**	
Total events	332		413				
Heterogeneity: Tau² = 0.00; Chi² = 5.41, df = 4 (P = 0.25); I² = 26%							
Test for overall effect: Z = 2.08 (P = 0.04)							
**Total (95% CI)**		**3148**		**3271**	**100.0%**	**-0.04 [-0.06, -0.02]**	
Total events	584		735				
Heterogeneity: Tau² = 0.00; Chi² = 13.88, df = 10 (P = 0.18); I² = 28%							
Test for overall effect: Z = 3.42 (P = 0.0006)							
Test for subgroup differences: Chi² = 0.37, df = 1 (P = 0.54), I² = 0%							

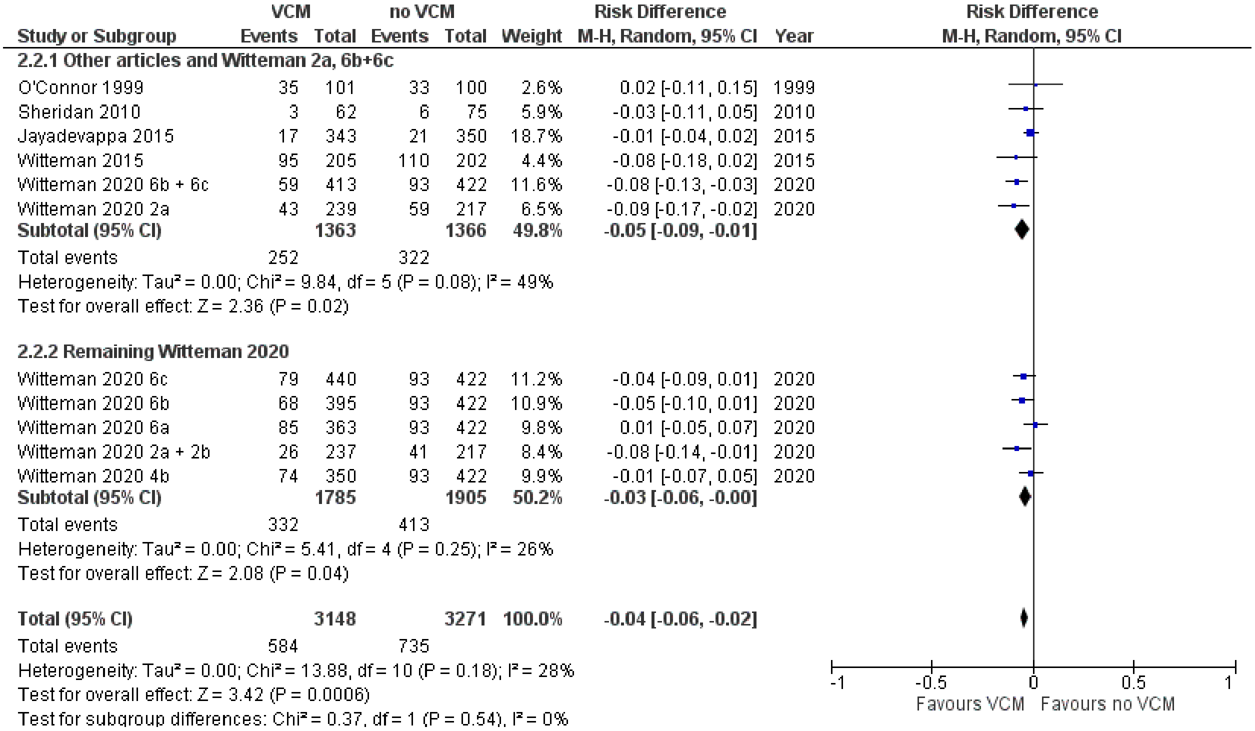

Figure S15. Risk of Values-Incongruent Decisions with Witteman 2020 2a + 2b and 4b

Study or Subgroup	VCM Events	VCM Total	no VCM Events	no VCM Total	Weight	Risk Difference M-H, Random, 95% CI	Year
**2.2.1 Other articles and Witteman 2a+2b, 4b**							
O'Connor 1999	35	101	33	100	2.6%	0.02 [-0.11, 0.15]	1999
Sheridan 2010	3	62	6	75	5.9%	-0.03 [-0.11, 0.05]	2010
Jayadevappa 2015	17	343	21	350	18.7%	-0.01 [-0.04, 0.02]	2015
Witteman 2015	95	205	110	202	4.4%	-0.08 [-0.18, 0.02]	2015
Witteman 2020 2a + 2b	26	237	41	217	8.4%	-0.08 [-0.14, -0.01]	2020
Witteman 2020 4b	74	350	93	422	9.9%	-0.01 [-0.07, 0.05]	2020
**Subtotal (95% CI)**		**1298**		**1366**	**49.9%**	**-0.03 [-0.05, 0.00]**	
Total events	250		304				
Heterogeneity: Tau² = 0.00; Chi² = 5.64, df = 5 (P = 0.34); I² = 11%							
Test for overall effect: Z = 1.95 (P = 0.05)							
**2.2.2 Remaining Witteman 2020**							
Witteman 2020 6b + 6c	59	413	93	422	11.6%	-0.08 [-0.13, -0.03]	2020
Witteman 2020 2a	43	239	59	217	6.5%	-0.09 [-0.17, -0.02]	2020
Witteman 2020 6c	79	440	93	422	11.2%	-0.04 [-0.09, 0.01]	2020
Witteman 2020 6b	68	395	93	422	10.9%	-0.05 [-0.10, 0.01]	2020
Witteman 2020 6a	85	363	93	422	9.8%	0.01 [-0.05, 0.07]	2020
**Subtotal (95% CI)**		**1850**		**1905**	**50.1%**	**-0.05 [-0.08, -0.01]**	
Total events	334		431				
Heterogeneity: Tau² = 0.00; Chi² = 6.81, df = 4 (P = 0.15); I² = 41%							
Test for overall effect: Z = 2.73 (P = 0.006)							
**Total (95% CI)**		**3148**		**3271**	**100.0%**	**-0.04 [-0.06, -0.02]**	
Total events	584		735				
Heterogeneity: Tau² = 0.00; Chi² = 13.88, df = 10 (P = 0.18); I² = 28%							
Test for overall effect: Z = 3.42 (P = 0.0006)							
Test for subgroup differences: Chi² = 0.82, df = 1 (P = 0.37), I² = 0%							

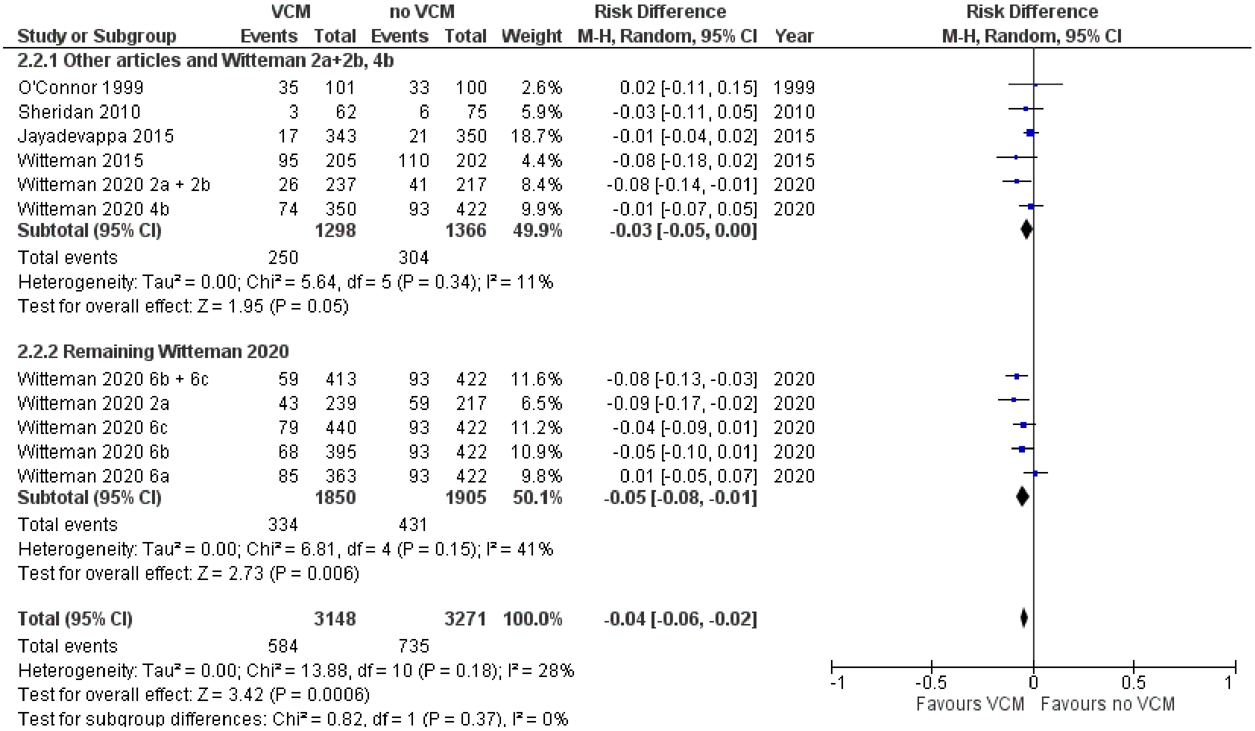

Figure S16. Risk of Values-Incongruent Decisions with Witteman 2020 2a + 2b and 6a

Study or Subgroup	VCM Events	VCM Total	no VCM Events	no VCM Total	Weight	Risk Difference M-H, Random, 95% CI	Year
**2.2.1 Other articles and Witteman 2a+2b, 6a**							
O'Connor 1999	35	101	33	100	2.6%	0.02 [-0.11, 0.15]	1999
Sheridan 2010	3	62	6	75	5.9%	-0.03 [-0.11, 0.05]	2010
Jayadevappa 2015	17	343	21	350	18.7%	-0.01 [-0.04, 0.02]	2015
Witteman 2015	95	205	110	202	4.4%	-0.08 [-0.18, 0.02]	2015
Witteman 2020 6a	85	363	93	422	9.8%	0.01 [-0.05, 0.07]	2020
Witteman 2020 2a + 2b	26	237	41	217	8.4%	-0.08 [-0.14, -0.01]	2020
**Subtotal (95% CI)**		**1311**		**1366**	**49.8%**	**-0.02 [-0.06, 0.01]**	
Total events	261		304				
Heterogeneity: Tau² = 0.00; Chi² = 6.71, df = 5 (P = 0.24); I² = 25%							
Test for overall effect: Z = 1.55 (P = 0.12)							
**2.2.2 Remaining Witteman 2020**							
Witteman 2020 4b	74	350	93	422	9.9%	-0.01 [-0.07, 0.05]	2020
Witteman 2020 6b + 6c	59	413	93	422	11.6%	-0.08 [-0.13, -0.03]	2020
Witteman 2020 2a	43	239	59	217	6.5%	-0.09 [-0.17, -0.02]	2020
Witteman 2020 6c	79	440	93	422	11.2%	-0.04 [-0.09, 0.01]	2020
Witteman 2020 6b	68	395	93	422	10.9%	-0.05 [-0.10, 0.01]	2020
**Subtotal (95% CI)**		**1837**		**1905**	**50.2%**	**-0.05 [-0.08, -0.02]**	
Total events	323		431				
Heterogeneity: Tau² = 0.00; Chi² = 4.25, df = 4 (P = 0.37); I² = 6%							
Test for overall effect: Z = 3.78 (P = 0.0002)							
**Total (95% CI)**		**3148**		**3271**	**100.0%**	**-0.04 [-0.06, -0.02]**	
Total events	584		735				
Heterogeneity: Tau² = 0.00; Chi² = 13.88, df = 10 (P = 0.18); I² = 28%							
Test for overall effect: Z = 3.42 (P = 0.0006)							
Test for subgroup differences: Chi² = 1.58, df = 1 (P = 0.21), I² = 36.8%							

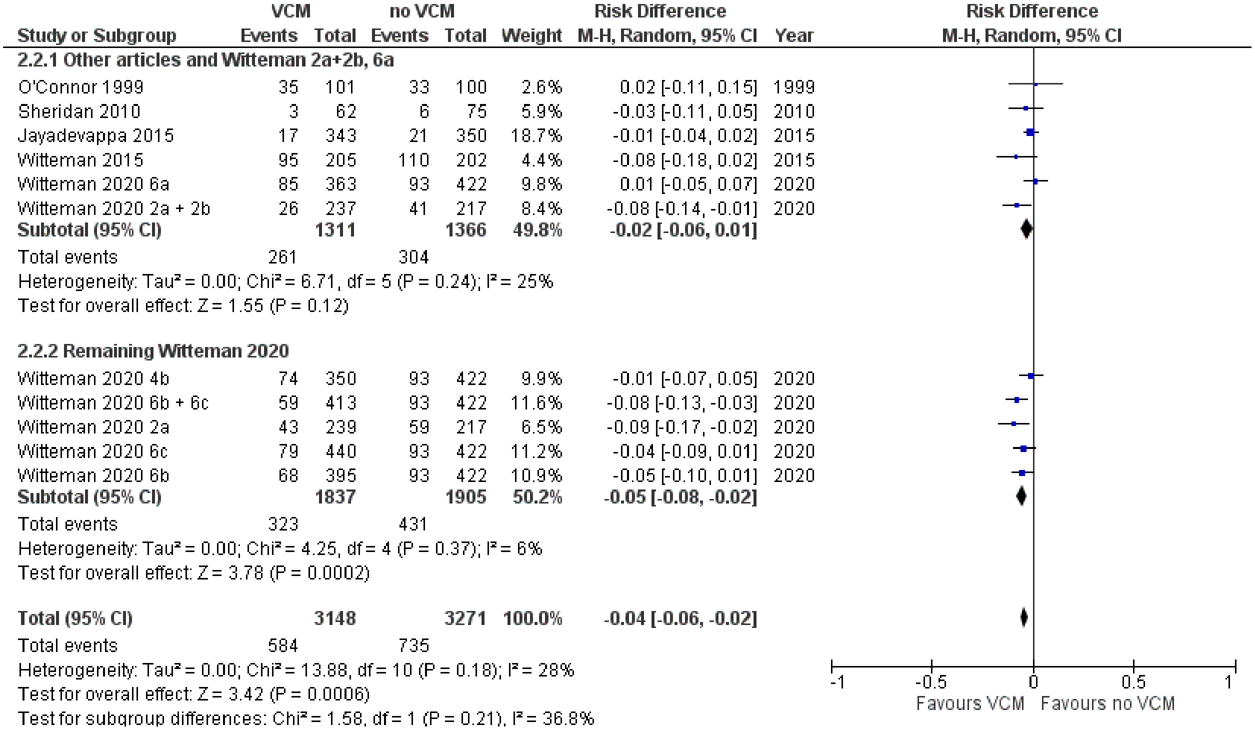

Figure S17. Risk of Values-Incongruent Decisions with Witteman 2020 2a + 2b and 6b

Study or Subgroup	VCM Events	VCM Total	no VCM Events	no VCM Total	Weight	Risk Difference M-H, Random, 95% CI	Year
**2.2.1 Other articles and Witteman 2a+2b, 6b**							
O'Connor 1999	35	101	33	100	2.6%	0.02 [-0.11, 0.15]	1999
Sheridan 2010	3	62	6	75	5.9%	-0.03 [-0.11, 0.05]	2010
Jayadevappa 2015	17	343	21	350	18.7%	-0.01 [-0.04, 0.02]	2015
Witteman 2015	95	205	110	202	4.4%	-0.08 [-0.18, 0.02]	2015
Witteman 2020 6b	68	395	93	422	10.9%	-0.05 [-0.10, 0.01]	2020
Witteman 2020 2a + 2b	26	237	41	217	8.4%	-0.08 [-0.14, -0.01]	2020
**Subtotal (95% CI)**		**1343**		**1366**	**50.9%**	**-0.04 [-0.07, -0.01]**	
Total events	244		304				
Heterogeneity: Tau² = 0.00; Chi² = 6.24, df = 5 (P = 0.28); I² = 20%							
Test for overall effect: Z = 2.44 (P = 0.01)							
**2.2.2 Remaining Witteman 2020**							
Witteman 2020 2a	43	239	59	217	6.5%	-0.09 [-0.17, -0.02]	2020
Witteman 2020 6c	79	440	93	422	11.2%	-0.04 [-0.09, 0.01]	2020
Witteman 2020 6b + 6c	59	413	93	422	11.6%	-0.08 [-0.13, -0.03]	2020
Witteman 2020 4b	74	350	93	422	9.9%	-0.01 [-0.07, 0.05]	2020
Witteman 2020 6a	85	363	93	422	9.8%	0.01 [-0.05, 0.07]	2020
**Subtotal (95% CI)**		**1805**		**1905**	**49.1%**	**-0.04 [-0.08, -0.00]**	
Total events	340		431				
Heterogeneity: Tau² = 0.00; Chi² = 8.08, df = 4 (P = 0.09); I² = 50%							
Test for overall effect: Z = 2.06 (P = 0.04)							
**Total (95% CI)**		**3148**		**3271**	**100.0%**	**-0.04 [-0.06, -0.02]**	
Total events	584		735				
Heterogeneity: Tau² = 0.00; Chi² = 13.88, df = 10 (P = 0.18); I² = 28%							
Test for overall effect: Z = 3.42 (P = 0.0006)							
Test for subgroup differences: Chi² = 0.02, df = 1 (P = 0.90), I² = 0%							

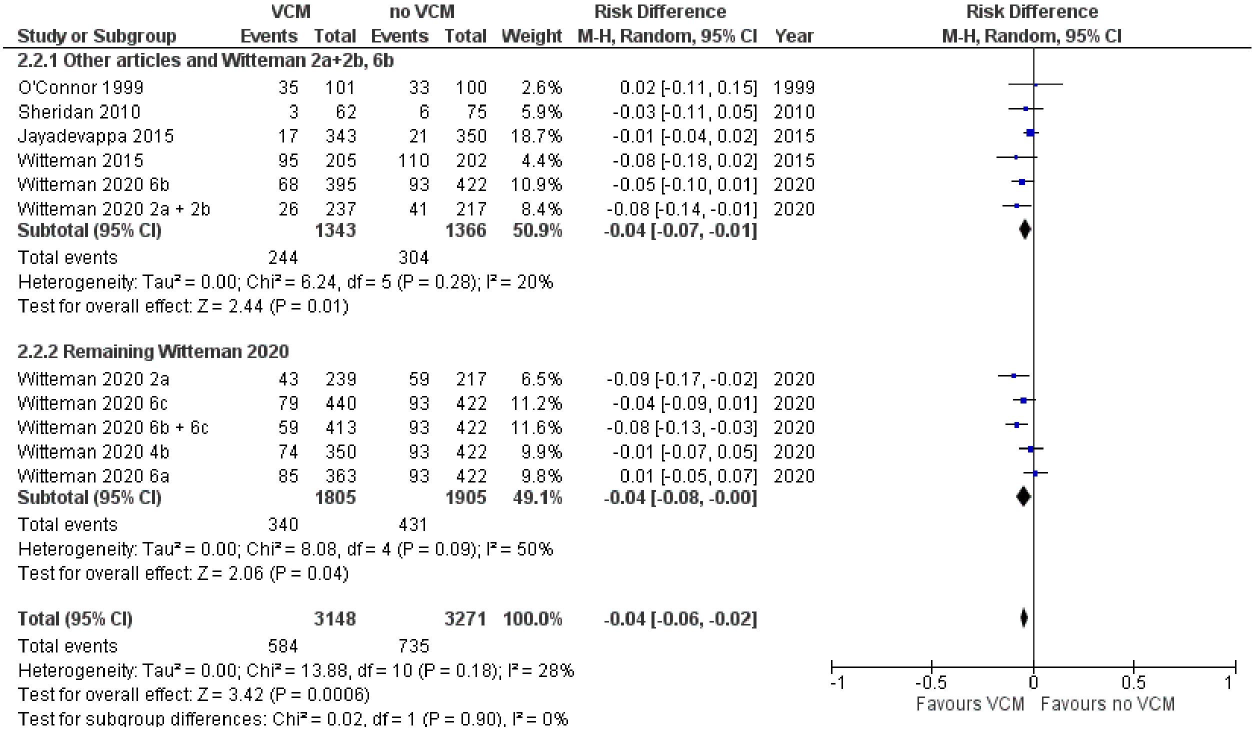

Figure S18. Risk of Values-Incongruent Decisions with Witteman 2020 2a + 2b and 6c

Study or Subgroup	VCM Events	VCM Total	no VCM Events	no VCM Total	Weight	Risk Difference M-H, Random, 95% CI	Year
**2.2.1 Other articles and Witteman 2a+2b, 6c**							
O'Connor 1999	35	101	33	100	2.6%	0.02 [-0.11, 0.15]	1999
Sheridan 2010	3	62	6	75	5.9%	-0.03 [-0.11, 0.05]	2010
Jayadevappa 2015	17	343	21	350	18.7%	-0.01 [-0.04, 0.02]	2015
Witteman 2015	95	205	110	202	4.4%	-0.08 [-0.18, 0.02]	2015
Witteman 2020 6c	79	440	93	422	11.2%	-0.04 [-0.09, 0.01]	2020
Witteman 2020 2a + 2b	26	237	41	217	8.4%	-0.08 [-0.14, -0.01]	2020
**Subtotal (95% CI)**		**1388**		**1366**	**51.2%**	**-0.03 [-0.06, -0.01]**	
Total events	255		304				
Heterogeneity: Tau² = 0.00; Chi² = 5.87, df = 5 (P = 0.32); I² = 15%							
Test for overall effect: Z = 2.40 (P = 0.02)							
**2.2.2 Remaining Witteman 2020**							
Witteman 2020 6b	68	395	93	422	10.9%	-0.05 [-0.10, 0.01]	2020
Witteman 2020 2a	43	239	59	217	6.5%	-0.09 [-0.17, -0.02]	2020
Witteman 2020 6a	85	363	93	422	9.8%	0.01 [-0.05, 0.07]	2020
Witteman 2020 4b	74	350	93	422	9.9%	-0.01 [-0.07, 0.05]	2020
Witteman 2020 6b + 6c	59	413	93	422	11.6%	-0.08 [-0.13, -0.03]	2020
**Subtotal (95% CI)**		**1760**		**1905**	**48.8%**	**-0.04 [-0.08, -0.00]**	
Total events	329		431				
Heterogeneity: Tau² = 0.00; Chi² = 8.17, df = 4 (P = 0.09); I² = 51%							
Test for overall effect: Z = 2.12 (P = 0.03)							
**Total (95% CI)**		**3148**		**3271**	**100.0%**	**-0.04 [-0.06, -0.02]**	
Total events	584		735				
Heterogeneity: Tau² = 0.00; Chi² = 13.88, df = 10 (P = 0.18); I² = 28%							
Test for overall effect: Z = 3.42 (P = 0.0006)							
Test for subgroup differences: Chi² = 0.09, df = 1 (P = 0.77), I² = 0%							

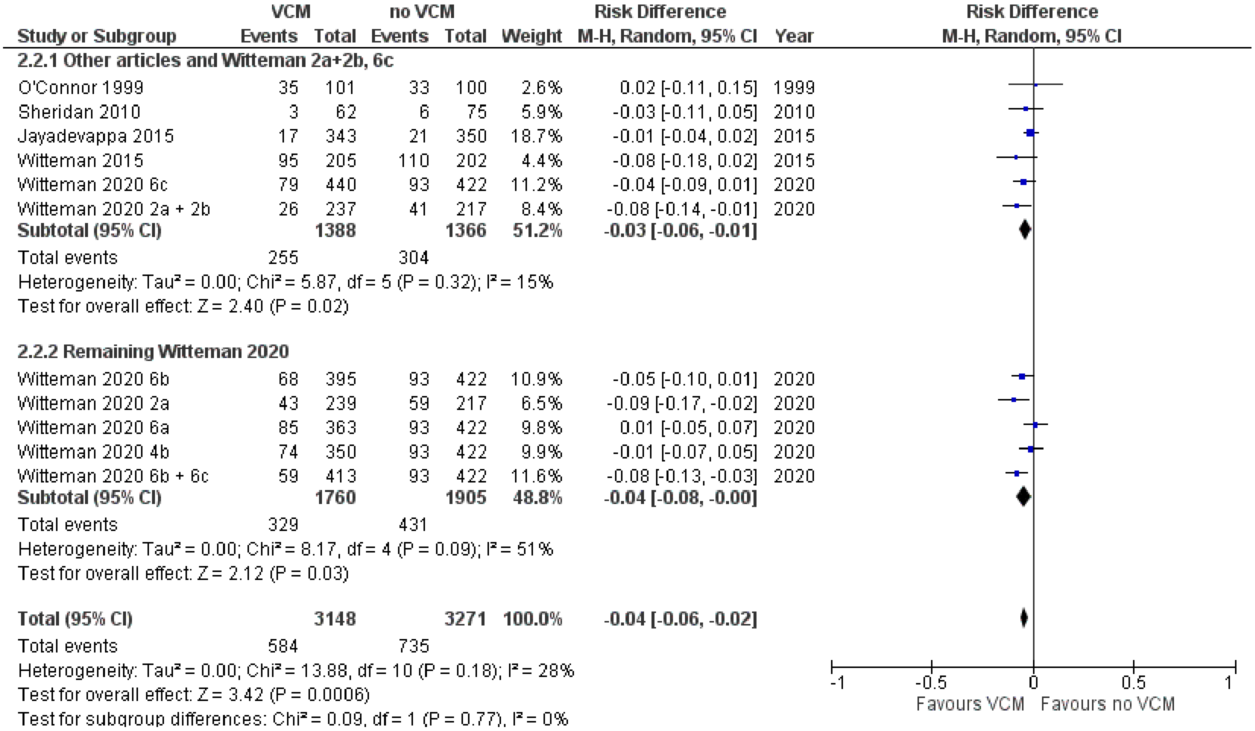

Figure S19. Risk of Values-Incongruent Decisions with Witteman 2020 2a + 2b and 6b + 6c

Study or Subgroup	VCM Events	VCM Total	no VCM Events	no VCM Total	Weight	Risk Difference M-H, Random, 95% CI	Year
**2.2.1 Other articles and Witteman 2a+2b, 6b+6c**							
O'Connor 1999	35	101	33	100	2.6%	0.02 [-0.11, 0.15]	1999
Sheridan 2010	3	62	6	75	5.9%	-0.03 [-0.11, 0.05]	2010
Jayadevappa 2015	17	343	21	350	18.7%	-0.01 [-0.04, 0.02]	2015
Witteman 2015	95	205	110	202	4.4%	-0.08 [-0.18, 0.02]	2015
Witteman 2020 6b + 6c	59	413	93	422	11.6%	-0.08 [-0.13, -0.03]	2020
Witteman 2020 2a + 2b	26	237	41	217	8.4%	-0.08 [-0.14, -0.01]	2020
**Subtotal (95% CI)**		**1361**		**1366**	**51.6%**	**-0.05 [-0.08, -0.01]**	
Total events	235		304				
Heterogeneity: Tau² = 0.00; Chi² = 9.03, df = 5 (P = 0.11); I² = 45%							
Test for overall effect: Z = 2.47 (P = 0.01)							
**2.2.2 Remaining Witteman 2020**							
Witteman 2020 6a	85	363	93	422	9.8%	0.01 [-0.05, 0.07]	2020
Witteman 2020 4b	74	350	93	422	9.9%	-0.01 [-0.07, 0.05]	2020
Witteman 2020 6c	79	440	93	422	11.2%	-0.04 [-0.09, 0.01]	2020
Witteman 2020 6b	68	395	93	422	10.9%	-0.05 [-0.10, 0.01]	2020
Witteman 2020 2a	43	239	59	217	6.5%	-0.09 [-0.17, -0.02]	2020
**Subtotal (95% CI)**		**1787**		**1905**	**48.4%**	**-0.03 [-0.06, -0.00]**	
Total events	349		431				
Heterogeneity: Tau² = 0.00; Chi² = 5.72, df = 4 (P = 0.22); I² = 30%							
Test for overall effect: Z = 1.97 (P = 0.05)							
**Total (95% CI)**		**3148**		**3271**	**100.0%**	**-0.04 [-0.06, -0.02]**	
Total events	584		735				
Heterogeneity: Tau² = 0.00; Chi² = 13.88, df = 10 (P = 0.18); I² = 28%							
Test for overall effect: Z = 3.42 (P = 0.0006)							
Test for subgroup differences: Chi² = 0.32, df = 1 (P = 0.57), I² = 0%							

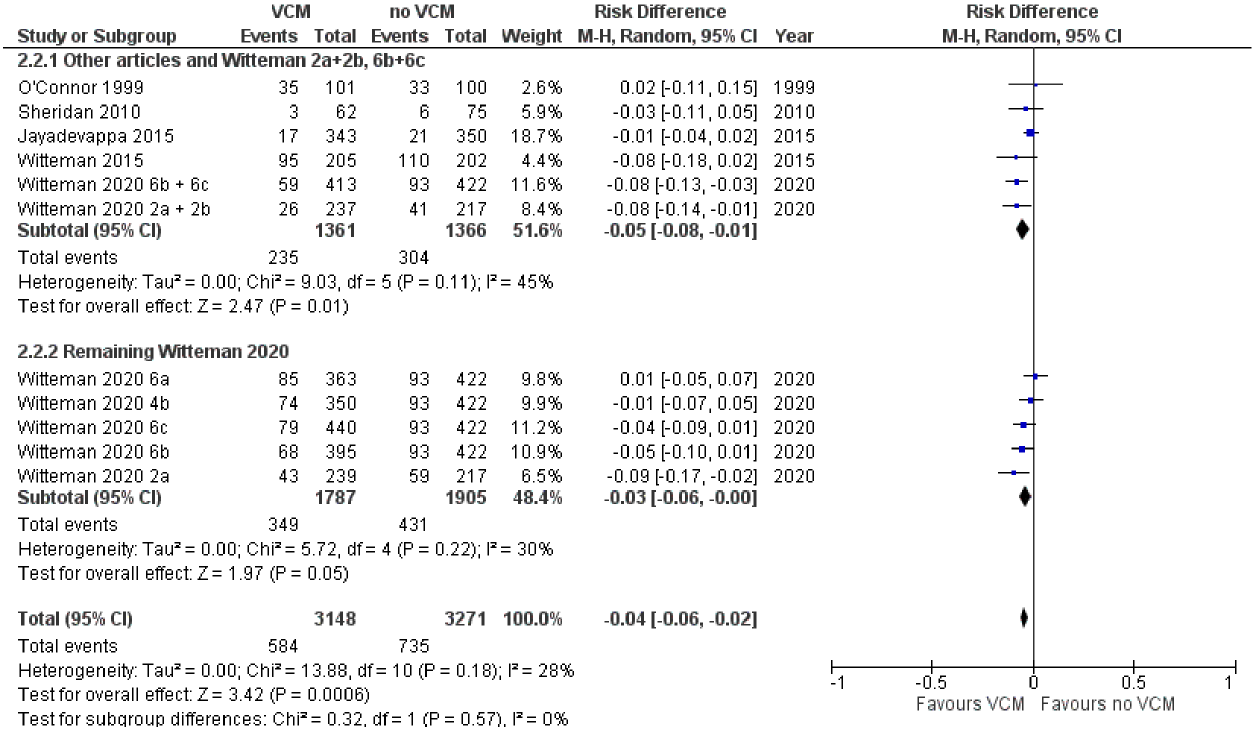

Figure S20. Risk of Values-Incongruent Decisions for Witteman 2020 Studies (Fixed Effects Model)

Study or Subgroup	VCM Events	VCM Total	No VCM Events	No VCM Total	Weight	Risk Difference M-H, Fixed, 95% CI	Year
Witteman 2020 6c	79	440	93	422	17.3%	-0.04 [-0.09, 0.01]	2020
Witteman 2020 2a	43	239	59	217	9.2%	-0.09 [-0.17, -0.02]	2020
Witteman 2020 2a + 2b	26	237	41	217	9.1%	-0.08 [-0.14, -0.01]	2020
Witteman 2020 4b	74	350	93	422	15.4%	-0.01 [-0.07, 0.05]	2020
Witteman 2020 6a	85	363	93	422	15.7%	0.01 [-0.05, 0.07]	2020
Witteman 2020 6b	68	395	93	422	16.4%	-0.05 [-0.10, 0.01]	2020
Witteman 2020 6b + 6c	59	413	93	422	16.8%	-0.08 [-0.13, -0.03]	2020
**Total (95% CI)**		**2437**		**2544**	**100.0%**	**-0.04 [-0.07, -0.02]**	
Total events	434		565				
Heterogeneity: Chi² = 9.37, df = 6 (P = 0.15); I² = 36%							
Test for overall effect: Z = 3.79 (P = 0.0002)							

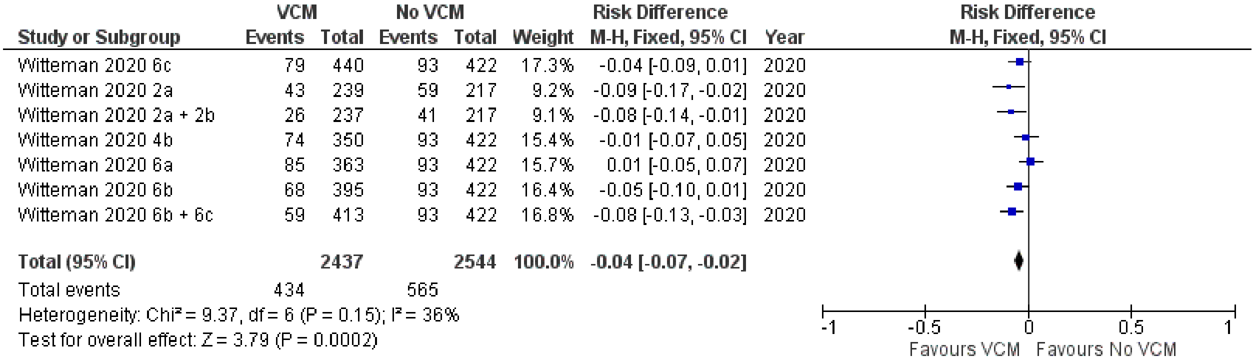

Figure S21. Decisional Conflict with Witteman 2020 4b

Study or Subgroup	VCM Mean	VCM SD	VCM Total	no VCM Mean	no VCM SD	no VCM Total	Weight	Std. Mean Difference IV, Random, 95% CI	Year
**1.1.1 Other articles and Witteman 2020 4b**									
Montgomery 2003	27.6	12.1	100	38.9	18.3	112	5.7%	-0.72 [-1.00, -0.44]	2003
Montgomery 2007	23.6	15.1	198	27.8	14.6	201	7.7%	-0.28 [-0.48, -0.09]	2007
Frosh 2008	27.95	13.45	153	30	10.77	151	6.9%	-0.17 [-0.39, 0.06]	2008
Kuppermann 2014	12.9	14.1	357	13.8	15.6	353	9.1%	-0.06 [-0.21, 0.09]	2014
Garvelink 2014 1	43.6	14.2	70	40.9	11.6	70	4.6%	0.21 [-0.13, 0.54]	2014
Garvelink 2014 2	41.6	9.5	66	44.1	12.3	65	4.4%	-0.23 [-0.57, 0.12]	2014
Shirk 2017	2.5	0.9	59	2.2	0.9	63	4.2%	0.33 [-0.03, 0.69]	2017
Epstein 2018	9.09	2.35	61	9.48	2.23	61	4.3%	-0.17 [-0.52, 0.19]	2018
Hopkin 2019	14.59	15.04	122	23.13	20.34	136	6.4%	-0.47 [-0.72, -0.22]	2019
Witteman 2020 4b	35	28	350	42	27	422	9.2%	-0.25 [-0.40, -0.11]	2020
**Subtotal (95% CI)**			**1536**			**1634**	**62.5%**	**-0.20 [-0.35, -0.04]**	
Heterogeneity: Tau² = 0.04; Chi² = 36.98, df = 9 (P < 0.0001); I² = 76%									
Test for overall effect: Z = 2.53 (P = 0.01)									
**1.1.2 Remaining Witteman 2020**									
Witteman 2020 6a	35	27	363	42	27	422	9.3%	-0.26 [-0.40, -0.12]	2020
Witteman 2020 6b	39	27	395	42	27	422	9.4%	-0.11 [-0.25, 0.03]	2020
Witteman 2020 6c	36	26	440	42	27	422	9.5%	-0.23 [-0.36, -0.09]	2020
Witteman 2020 6b + 6c	36	27	413	42	27	422	9.4%	-0.22 [-0.36, -0.09]	2020
**Subtotal (95% CI)**			**1611**			**1688**	**37.5%**	**-0.20 [-0.27, -0.14]**	
Heterogeneity: Tau² = 0.00; Chi² = 2.52, df = 3 (P = 0.47); I² = 0%									
Test for overall effect: Z = 5.84 (P < 0.00001)									
**Total (95% CI)**			**3147**			**3322**	**100.0%**	**-0.20 [-0.29, -0.11]**	
Heterogeneity: Tau² = 0.02; Chi² = 39.50, df = 13 (P = 0.0002); I² = 67%									
Test for overall effect: Z = 4.35 (P < 0.0001)									
Test for subgroup differences: Chi² = 0.01, df = 1 (P = 0.93), I² = 0%									

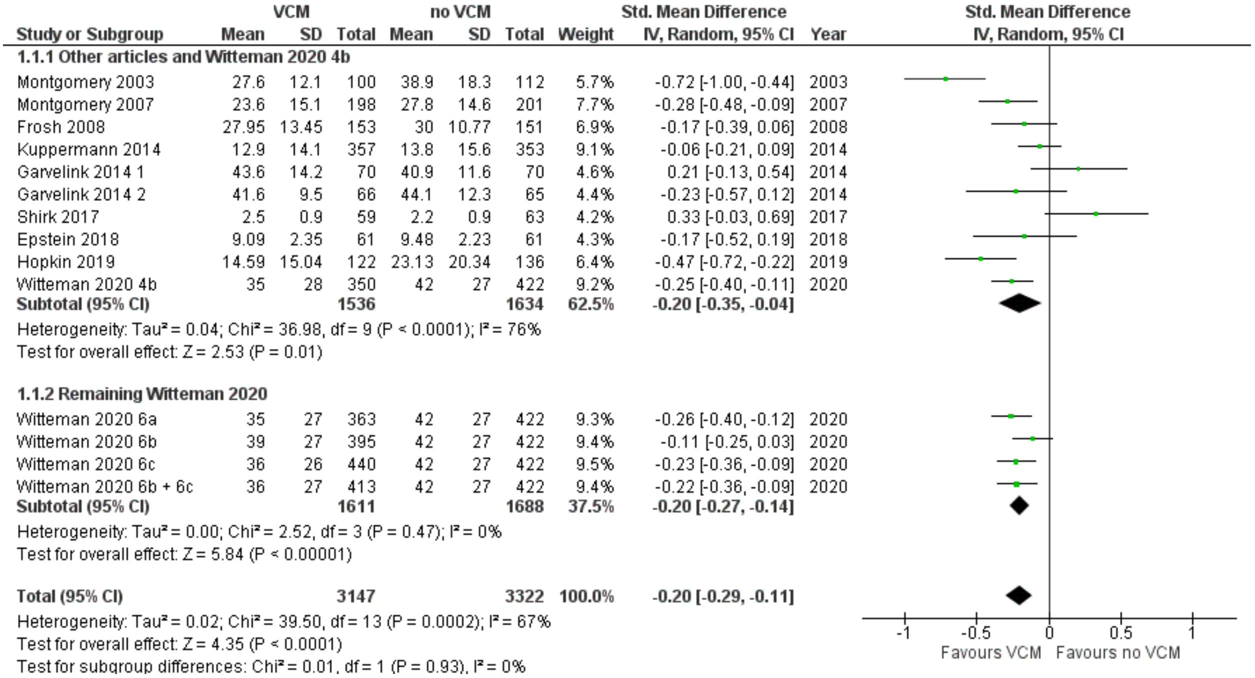

Figure S22. Decisional Conflict with Witteman 2020 6a

Study or Subgroup	VCM Mean	VCM SD	VCM Total	no VCM Mean	no VCM SD	no VCM Total	Weight	Std. Mean Difference IV, Random, 95% CI	Year
**1.1.1 Other articles and Witteman 2020 6a**									
Montgomery 2003	27.6	12.1	100	38.9	18.3	112	5.7%	-0.72 [-1.00, -0.44]	2003
Montgomery 2007	23.6	15.1	198	27.8	14.6	201	7.7%	-0.28 [-0.48, -0.09]	2007
Frosh 2008	27.95	13.45	153	30	10.77	151	6.9%	-0.17 [-0.39, 0.06]	2008
Kuppermann 2014	12.9	14.1	357	13.8	15.6	353	9.1%	-0.06 [-0.21, 0.09]	2014
Garvelink 2014 1	43.6	14.2	70	40.9	11.6	70	4.6%	0.21 [-0.13, 0.54]	2014
Garvelink 2014 2	41.6	9.5	66	44.1	12.3	65	4.4%	-0.23 [-0.57, 0.12]	2014
Shirk 2017	2.5	0.9	59	2.2	0.9	63	4.2%	0.33 [-0.03, 0.69]	2017
Epstein 2018	9.09	2.35	61	9.48	2.23	61	4.3%	-0.17 [-0.52, 0.19]	2018
Hopkin 2019	14.59	15.04	122	23.13	20.34	136	6.4%	-0.47 [-0.72, -0.22]	2019
Witteman 2020 6a	35	27	363	42	27	422	9.3%	-0.26 [-0.40, -0.12]	2020
**Subtotal (95% CI)**			**1549**			**1634**	**62.5%**	**-0.20 [-0.35, -0.05]**	
Heterogeneity: Tau² = 0.04; Chi² = 37.08, df = 9 (P < 0.0001); I² = 76%									
Test for overall effect: Z = 2.54 (P = 0.01)									
**1.1.2 Remaining Witteman 2020**									
Witteman 2020 6b	39	27	395	42	27	422	9.4%	-0.11 [-0.25, 0.03]	2020
Witteman 2020 6c	36	26	440	42	27	422	9.5%	-0.23 [-0.36, -0.09]	2020
Witteman 2020 6b + 6c	36	27	413	42	27	422	9.4%	-0.22 [-0.36, -0.09]	2020
Witteman 2020 4b	35	28	350	42	27	422	9.2%	-0.25 [-0.40, -0.11]	2020
**Subtotal (95% CI)**			**1598**			**1688**	**37.5%**	**-0.20 [-0.27, -0.13]**	
Heterogeneity: Tau² = 0.00; Chi² = 2.42, df = 3 (P = 0.49); I² = 0%									
Test for overall effect: Z = 5.80 (P < 0.00001)									
**Total (95% CI)**			**3147**			**3322**	**100.0%**	**-0.20 [-0.29, -0.11]**	
Heterogeneity: Tau² = 0.02; Chi² = 39.50, df = 13 (P = 0.0002); I² = 67%									
Test for overall effect: Z = 4.35 (P < 0.0001)									
Test for subgroup differences: Chi² = 0.01, df = 1 (P = 0.94), I² = 0%									

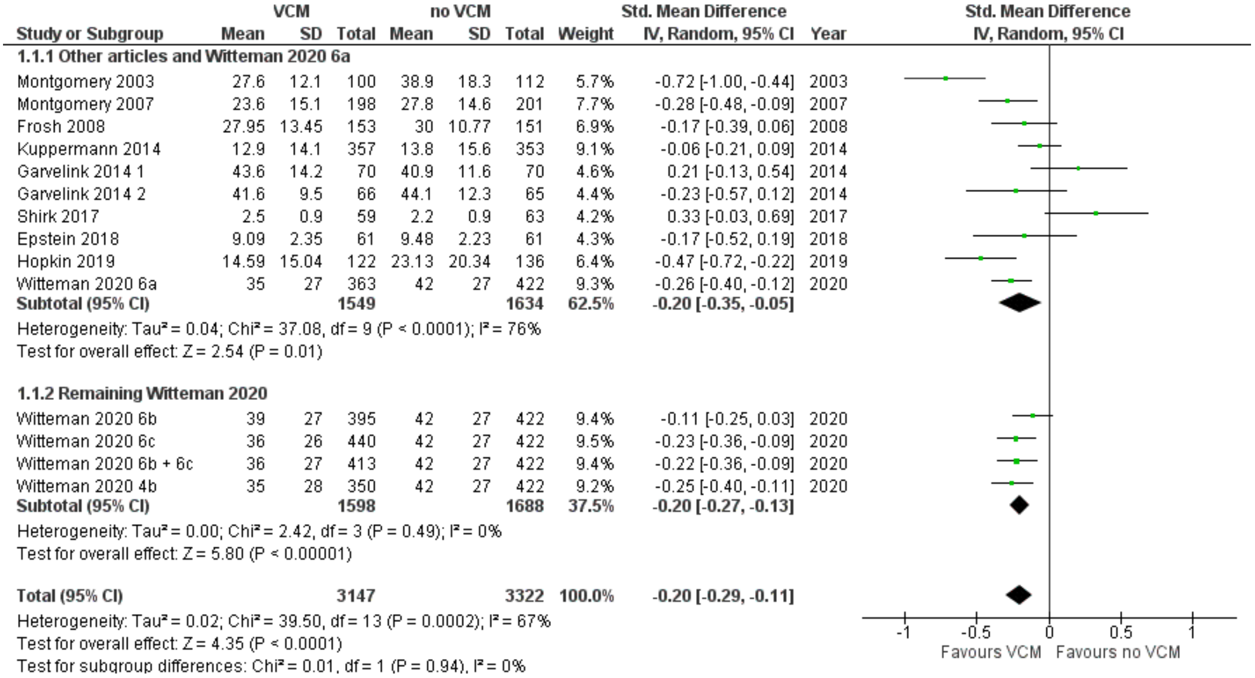

Figure S23. Decisional Conflict with Witteman 2020 6b

Study or Subgroup	VCM Mean	VCM SD	VCM Total	no VCM Mean	no VCM SD	no VCM Total	Weight	Std. Mean Difference IV, Random, 95% CI	Year
**1.1.1 Other articles and Witteman 2020 6b**									
Montgomery 2003	27.6	12.1	100	38.9	18.3	112	5.7%	-0.72 [-1.00, -0.44]	2003
Montgomery 2007	23.6	15.1	198	27.8	14.6	201	7.7%	-0.28 [-0.48, -0.09]	2007
Frosh 2008	27.95	13.45	153	30	10.77	151	6.9%	-0.17 [-0.39, 0.06]	2008
Kuppermann 2014	12.9	14.1	357	13.8	15.6	353	9.1%	-0.06 [-0.21, 0.09]	2014
Garvelink 2014 1	43.6	14.2	70	40.9	11.6	70	4.6%	0.21 [-0.13, 0.54]	2014
Garvelink 2014 2	41.6	9.5	66	44.1	12.3	65	4.4%	-0.23 [-0.57, 0.12]	2014
Shirk 2017	2.5	0.9	59	2.2	0.9	63	4.2%	0.33 [-0.03, 0.69]	2017
Epstein 2018	9.09	2.35	61	9.48	2.23	61	4.3%	-0.17 [-0.52, 0.19]	2018
Hopkin 2019	14.59	15.04	122	23.13	20.34	136	6.4%	-0.47 [-0.72, -0.22]	2019
Witteman 2020 6b	39	27	395	42	27	422	9.4%	-0.11 [-0.25, 0.03]	2020
**Subtotal (95% CI)**			**1581**			**1634**	**62.6%**	**-0.18 [-0.33, -0.03]**	
Heterogeneity: Tau² = 0.04; Chi² = 37.28, df = 9 (P < 0.0001); I² = 76%									
Test for overall effect: Z = 2.30 (P = 0.02)									
**1.1.2 Remaining Witteman 2020**									
Witteman 2020 6c	36	26	440	42	27	422	9.5%	-0.23 [-0.36, -0.09]	2020
Witteman 2020 6b + 6c	36	27	413	42	27	422	9.4%	-0.22 [-0.36, -0.09]	2020
Witteman 2020 4b	35	28	350	42	27	422	9.2%	-0.25 [-0.40, -0.11]	2020
Witteman 2020 6a	35	27	363	42	27	422	9.3%	-0.26 [-0.40, -0.12]	2020
**Subtotal (95% CI)**			**1566**			**1688**	**37.4%**	**-0.24 [-0.31, -0.17]**	
Heterogeneity: Tau² = 0.00; Chi² = 0.22, df = 3 (P = 0.97); I² = 0%									
Test for overall effect: Z = 6.80 (P < 0.00001)									
**Total (95% CI)**			**3147**			**3322**	**100.0%**	**-0.20 [-0.29, -0.11]**	
Heterogeneity: Tau² = 0.02; Chi² = 39.50, df = 13 (P = 0.0002); I² = 67%									
Test for overall effect: Z = 4.35 (P < 0.0001)									
Test for subgroup differences: Chi² = 0.53, df = 1 (P = 0.47), I² = 0%									

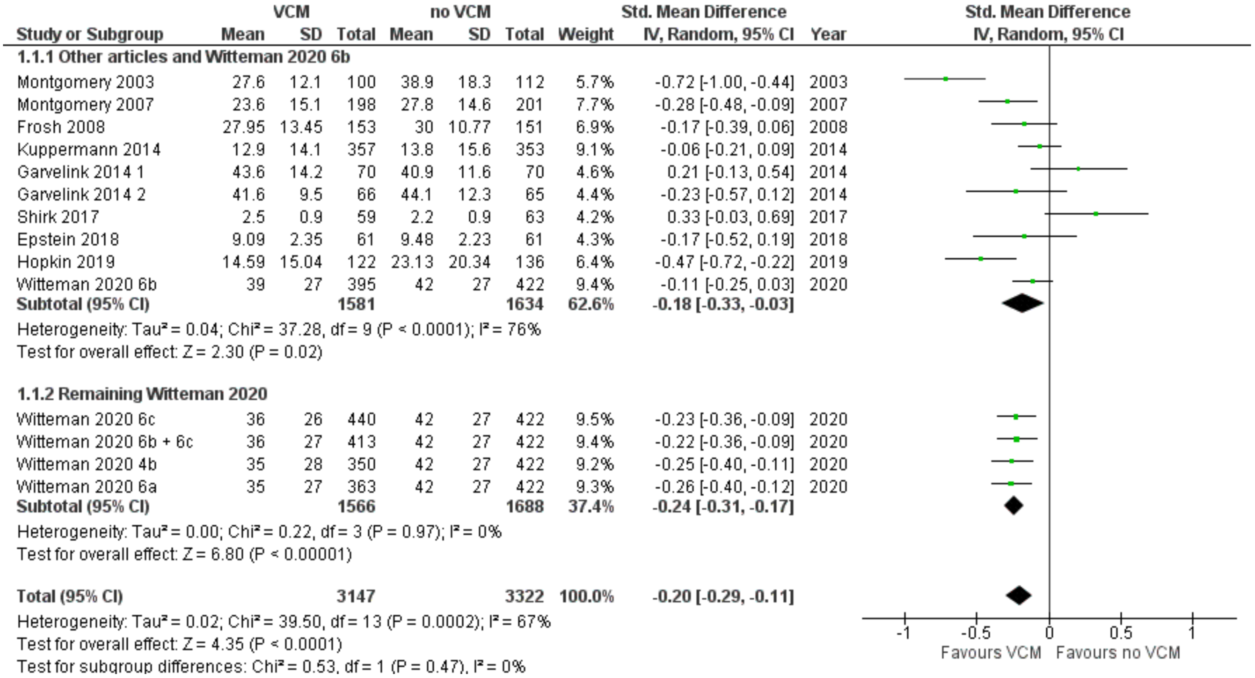

Figure S24. Decisional Conflict with Witteman 2020 6c

Study or Subgroup	VCM Mean	VCM SD	VCM Total	no VCM Mean	no VCM SD	no VCM Total	Weight	Std. Mean Difference IV, Random, 95% CI	Year
**1.1.1 Other articles and Witteman 2020 6c**									
Montgomery 2003	27.6	12.1	100	38.9	18.3	112	5.7%	-0.72 [-1.00, -0.44]	2003
Montgomery 2007	23.6	15.1	198	27.8	14.6	201	7.7%	-0.28 [-0.48, -0.09]	2007
Frosh 2008	27.95	13.45	153	30	10.77	151	6.9%	-0.17 [-0.39, 0.06]	2008
Kuppermann 2014	12.9	14.1	357	13.8	15.6	353	9.1%	-0.06 [-0.21, 0.09]	2014
Garvelink 2014 1	43.6	14.2	70	40.9	11.6	70	4.6%	0.21 [-0.13, 0.54]	2014
Garvelink 2014 2	41.6	9.5	66	44.1	12.3	65	4.4%	-0.23 [-0.57, 0.12]	2014
Shirk 2017	2.5	0.9	59	2.2	0.9	63	4.2%	0.33 [-0.03, 0.69]	2017
Epstein 2018	9.09	2.35	61	9.48	2.23	61	4.3%	-0.17 [-0.52, 0.19]	2018
Hopkin 2019	14.59	15.04	122	23.13	20.34	136	6.4%	-0.47 [-0.72, -0.22]	2019
Witteman 2020 6c	36	26	440	42	27	422	9.5%	-0.23 [-0.36, -0.09]	2020
**Subtotal (95% CI)**			**1626**			**1634**	**62.7%**	**-0.19 [-0.34, -0.04]**	
Heterogeneity: Tau² = 0.04; Chi² = 36.58, df = 9 (P < 0.0001); I² = 75%									
Test for overall effect: Z = 2.53 (P = 0.01)									
**1.1.2 Remaining Witteman 2020**									
Witteman 2020 6a	35	27	363	42	27	422	9.3%	-0.26 [-0.40, -0.12]	2020
Witteman 2020 6b	39	27	395	42	27	422	9.4%	-0.11 [-0.25, 0.03]	2020
Witteman 2020 6b + 6c	36	27	413	42	27	422	9.4%	-0.22 [-0.36, -0.09]	2020
Witteman 2020 4b	35	28	350	42	27	422	9.2%	-0.25 [-0.40, -0.11]	2020
**Subtotal (95% CI)**			**1521**			**1688**	**37.3%**	**-0.21 [-0.28, -0.14]**	
Heterogeneity: Tau² = 0.00; Chi² = 2.87, df = 3 (P = 0.41); I² = 0%									
Test for overall effect: Z = 5.93 (P < 0.00001)									
**Total (95% CI)**			**3147**			**3322**	**100.0%**	**-0.20 [-0.29, -0.11]**	
Heterogeneity: Tau² = 0.02; Chi² = 39.50, df = 13 (P = 0.0002); I² = 67%									
Test for overall effect: Z = 4.35 (P < 0.0001)									
Test for subgroup differences: Chi² = 0.04, df = 1 (P = 0.84), I² = 0%									

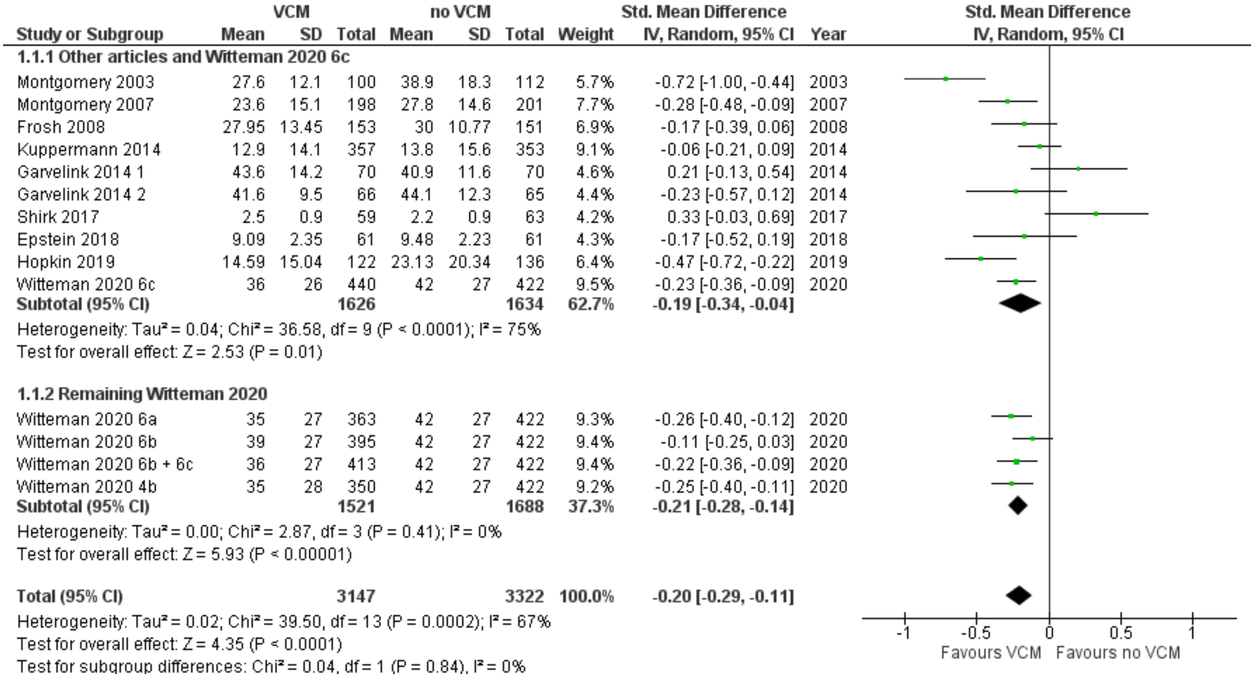

Figure S25. Decisional Conflict with Witteman 2020 6b + 6c

	VCM			no VCM				Std. Mean Difference		Std. Mean Difference
**Study or Subgroup**	**Mean**	**SD**	**Total**	**Mean**	**SD**	**Total**	**Weight**	**IV, Random, 95% CI**	**Year**	**IV, Random, 95% CI**
**1.1.1 Other articles and Witteman 2020 6b+6c**										
Montgomery 2003	27.6	12.1	100	38.9	18.3	112	5.7%	-0.72 [-1.00, -0.44]	2003	
Montgomery 2007	23.6	15.1	198	27.8	14.6	201	7.7%	-0.28 [-0.48, -0.09]	2007	
Frosh 2008	27.95	13.45	153	30	10.77	151	6.9%	-0.17 [-0.39, 0.06]	2008	
Kuppermann 2014	12.9	14.1	357	13.8	15.6	353	9.1%	-0.06 [-0.21, 0.09]	2014	
Garvelink 2014 1	43.6	14.2	70	40.9	11.6	70	4.6%	0.21 [-0.13, 0.54]	2014	
Garvelink 2014 2	41.6	9.5	66	44.1	12.3	65	4.4%	-0.23 [-0.57, 0.12]	2014	
Shirk 2017	2.5	0.9	59	2.2	0.9	63	4.2%	0.33 [-0.03, 0.69]	2017	
Epstein 2018	9.09	2.35	61	9.48	2.23	61	4.3%	-0.17 [-0.52, 0.19]	2018	
Hopkin 2019	14.59	15.04	122	23.13	20.34	136	6.4%	-0.47 [-0.72, -0.22]	2019	
Witteman 2020 6b + 6c	36	27	413	42	27	422	9.4%	-0.22 [-0.36, -0.09]	2020	
**Subtotal (95% CI)**			**1599**			**1634**	**62.7%**	**-0.19 [-0.34, -0.04]**		
Heterogeneity: Tau² = 0.04; Chi² = 36.53, df = 9 (P < 0.0001); I² = 75%										
Test for overall effect: Z = 2.52 (P = 0.01)										
**1.1.2 Remaining Witteman 2020**										
Witteman 2020 6a	35	27	363	42	27	422	9.3%	-0.26 [-0.40, -0.12]	2020	
Witteman 2020 6b	39	27	395	42	27	422	9.4%	-0.11 [-0.25, 0.03]	2020	
Witteman 2020 4b	35	28	350	42	27	422	9.2%	-0.25 [-0.40, -0.11]	2020	
Witteman 2020 6c	36	26	440	42	27	422	9.5%	-0.23 [-0.36, -0.09]	2020	
**Subtotal (95% CI)**			**1548**			**1688**	**37.3%**	**-0.21 [-0.28, -0.14]**		
Heterogeneity: Tau² = 0.00; Chi² = 2.89, df = 3 (P = 0.41); I² = 0%										
Test for overall effect: Z = 5.99 (P < 0.00001)										
**Total (95% CI)**			**3147**			**3322**	**100.0%**	**-0.20 [-0.29, -0.11]**		
Heterogeneity: Tau² = 0.02; Chi² = 39.50, df = 13 (P = 0.0002); I² = 67%										
Test for overall effect: Z = 4.35 (P < 0.0001)										
Test for subgroup differences: Chi² = 0.05, df = 1 (P = 0.82), I² = 0%										

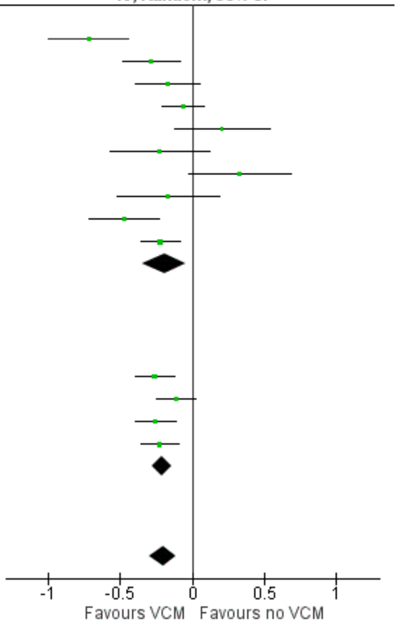

Figure S26. Decisional Conflict for Witteman 2020 Studies (Fixed Effects Model)

	VCM			No VCM				Mean Difference		Mean Difference
**Study or Subgroup**	**Mean**	**SD**	**Total**	**Mean**	**SD**	**Total**	**Weight**	**IV, Fixed, 95% CI**	**Year**	**IV, Fixed, 95% CI**
Witteman 2020 6b + 6c	36	27	413	42	27	422	20.6%	-6.00 [-9.66, -2.34]	2020	
Witteman 2020 6c	36	26	440	42	27	422	22.0%	-6.00 [-9.54, -2.46]	2020	
Witteman 2020 4b	35	28	350	42	27	422	18.1%	-7.00 [-10.90, -3.10]	2020	
Witteman 2020 6a	35	27	363	42	27	422	19.2%	-7.00 [-10.79, -3.21]	2020	
Witteman 2020 6b	39	27	395	42	27	422	20.1%	-3.00 [-6.70, 0.70]	2020	
**Total (95% CI)**			**1961**			**2110**	**100.0%**	**-5.77 [-7.43, -4.11]**		
Heterogeneity: Chi² = 2.97, df = 4 (P = 0.56); I² = 0%										
Test for overall effect: Z = 6.81 (P < 0.00001)										

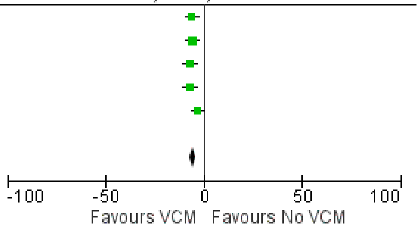

Supplement: sj-pdf-2-mdm-10.1177_0272989X211037946 – Supplemental material for Clarifying Values: An Updated and Expanded Systematic Review and Meta-Analysis [file sj-pdf-2-mdm-10.1177_0272989X211037946.pdf]
